# Investigation of Gene Regions Responsible for Drug Resistance in Clinical Isolates of *Mycobacterium tuberculosis* Complex Resistant to at Least Two First-Line Anti-Tuberculosis Drugs

**DOI:** 10.3390/pathogens15020222

**Published:** 2026-02-16

**Authors:** Mahmut Ulger, Nurcihan Biltekin, Seda Tezcan Ulger, Gonul Aslan

**Affiliations:** 1 Department of Pharmaceutical Microbiology, Faculty of Pharmacy, University of Mersin, 33169 Mersin, Türkiye; nur.biltekinn@hotmail.com; 2 Institute of Health Sciences, Department of Pharmaceutical Microbiology, University of Mersin, 33169 Mersin, Türkiye; 3 Department of Medical Microbiology, Faculty of Medicine, University of Mersin, 33343 Mersin, Türkiye; tezcanseda@mersin.edu.tr (S.T.U.); drgaslan@mersin.edu.tr (G.A.)

**Keywords:** *M. tuberculosis*, mutation, *rpsL* codon 70, *rpsL* codon 88, sequence analysis

## Abstract

Early and rapid diagnosis of drug resistance in tuberculosis (TB) plays a key role in reducing the spread of resistance and enabling effective treatment. The aim of this study was to investigate mutations in drug resistance-associated gene regions of *Mycobacterium tuberculosis* complex (MTBC) isolates resistant to at least two first-line anti-tuberculosis drugs through sequence analysis, in order to characterize the core molecular features of these strains in the region and to identify previously unreported, geographically distinct novel mutation sites. The drug susceptibility of 23 clinical isolates was assessed using the BACTEC MGIT 960 system, and resistance-associated point mutations were identified through DNA sequence analysis and comparison with GenBank reference sequences. AAG → AGG mutation was detected in the *rpsL* gene region at codon 43 (*n* = 7) and codon 88 (*n* = 1). Additionally, GAG → GCG point mutation was identified at codon 70 (*n* = 2), representing a new region not previously reported in the literature. The most frequent mutation was AGC → ACC at *katG* codon 315 (*n* = 10), followed by a C → T substitution at position −15 of the *inhA* promoter region (*n* = 4). Additionally, TCG → TTG at *rpoB* codon 531 (*n* = 4) and ATG → GTG at *embB* codon 306 (*n* = 1) were detected. The detection of resistance-associated mutations is essential for controlling drug-resistant tuberculosis. In this study, a novel *rpsL* mutation (GAG → GCG) at codon 70 and a previously unreported codon 88 mutation in our country were identified, contributing to the understanding of molecular resistance mechanisms and epidemiology.

## 1. Introduction

Tuberculosis (TB) is a bacterial disease caused by a single pathogen, *Mycobacterium tuberculosis* (*M. tuberculosis*). It is reported that approximately 10 million people worldwide contract TB each year, and despite being preventable and treatable, it causes the death of nearly two million people [1]. TB has a high mortality rate (50%) when left untreated. Approximately 88% of TB patients can be successfully treated with the recommended standard anti-TB drug regimens [1]. The first-line anti-TB drugs used in the treatment regimen are streptomycin (SM), isoniazid (INH), rifampicin (RIF), pyrazinamide (PZA), and ethambutol (EMB). The long-term and combined use of these drugs causes the emergence and spread of drug-resistant strains [2]. Mutations in the genome of *Mycobacterium tuberculosis* complex (MTBC) isolates are associated with resistance to widely used anti-TB drugs in TB treatment. The resulting changes at the genetic level caused by these mutations lead to the development of drug resistance [3]. Drug-resistant TB is a major problem worldwide, and negatively affects treatment and control programs [4]. Early and rapid diagnosis of drug resistance plays a key role in reducing its spread and enabling effective treatment [5]. SM, one of the first-line anti-TB drugs, is an aminoglycoside drug that inhibits protein synthesis. SM resistance is associated with mutations in the *rpsL*, *rrs*, and *gid* gene regions [5]. Mutations occurring in the *rrs* gene (which encodes the 16S rRNA of the ribosomal protein) and the *rpsL* gene (which encodes the ribosomal S12 protein) are primarily responsible for 60–70% of SM resistance [4]. INH is a prodrug that inhibits mycolic acid synthesis and is activated by the catalase/peroxidase enzyme encoded by the *katG* gene. In INH resistance, various gene regions such as *katG*, *inhA*, *kasA*, *ndh*, and *efpA* are responsible [3]. INH resistance most frequently occurs in the *katG* and *inhA* genes. Specifically, the S315T mutation in the *katG* gene is responsible for 50–90% of cases [3]. INH resistance reduces the likelihood of treatment success and increases the risk of resistance to other anti-TB drugs such as RIF and multidrug-resistant (MDR)-TB [6]. RIF is a drug commonly used in the treatment regimen, and the *rpoA*, *rpoB*, *rpoC*, and *rpoZ* gene regions are implicated in RIF resistance. Mutations occurring in the *rpoB* gene, which encodes the beta subunit of RNA polymerase, are responsible for approximately 90–100% of cases. The development of RIF resistance is a significant problem in the control of TB [7]. Furthermore, EMB is a drug that inhibits arabinosyl transferase in the cell wall. The arabinosyl transferase enzyme is encoded by the *embCAB* operon gene region and is associated with the biosynthesis of arabinogalactan and lipoarabinomannan. Mutations occur in the *embB* gene region in approximately 50–70% of *M. tuberculosis* isolates [8]. The early and rapid diagnosis of drug resistance plays a crucial role in reducing the spread of resistance and establishing effective treatment [9]. In recent years, it has become possible to conduct comprehensive research targeting specific resistance quickly using molecular methods [9,10,11].

This study aims to investigate, through sequence analysis, mutations in drug resistance-associated gene regions in *M. tuberculosis* complex (MTBC) isolates resistant to at least two first-line anti-TB drugs. The objectives are to characterize the core molecular features of these strains in the region, assess the coverage of routinely used targeted gene-based resistance testing for local isolates, and explore the presence of previously unreported, geographically distinct novel mutation sites. 

## 2. Materials and Methods

Ethical approval for this study was obtained from the Mersin University Rectorate, Clinical Research Ethics Committee Presidency, with approval number 2024/873 dated 18 September 2024. This study was conducted using archived clinical isolates of *M. tuberculosis*. All procedures involving pathogenic microorganisms were performed in compliance with national and international biosafety regulations under appropriate biosafety level (BSL) conditions and institutional laboratory safety guidelines.

### 2.1. Clinical Isolates 

The study included clinical isolates with MTBC growth detected in the culture of clinical specimens sent to the Mycobacteriology Laboratory of Mersin University Hospital between 2016 and 2024 with a suspicion of TB. Löwenstein–Jensen (LJ) medium and the BACTEC MGIT 960 (Becton Dickinson, Franklin Lakes, NJ, USA) liquid automated system were used for the culture process. According to the culture results, 616 MTBC clinical isolates were detected. The susceptibility of the clinical isolates to the first-line anti-TB drugs SM, INH, RIF, and EMB was determined using the BACTEC MGIT 960 system [12]. Of the 616 MTBC clinical isolates, 87 were resistant to at least one of the first-line anti-TB drugs. Twenty-three clinical isolates determined to be resistant to at least two of the first-line anti-TB drugs were included in the study.

### 2.2. DNA Extraction of M. tuberculosis

The passages from the culture collection of the clinical isolates included in the study, which were stored at 4 °C, were subcultured onto LJ medium and incubated at 37 °C for four weeks. The rapid DNA extraction protocol was applied to the *M. tuberculosis* colonies grown on LJ medium. One loopful of colony was suspended in 1 mL of sterile distilled water, and the suspension was held at 80 °C for 20 min to ensure bacterial lysis. The suspension was then centrifuged at 12,000× *g* for 10 min, and the supernatant was discarded. Following this, 200 μL of chloroform and 200 μL of sterile distilled water were added to the pellet, and the sample was centrifuged again at 12,000× *g* for 10 min. The supernatant portion was transferred to a new sterile microcentrifuge tube and stored at −20 °C until it was used for molecular studies [13].

### 2.3. Investigation of Gene Regions Responsible for Drug Resistance

#### 2.3.1. First-Line Anti-TB Primer Design

Nucleotide sequences of the primer used in this study are given in Table 1.

#### 2.3.2. Determining SM Resistance

The *rpsL* gene region was amplified with specific primers [14]. The standard PCR reaction for the gene region was performed in a final volume of 50 µL, containing 5 µL of 10× PCR buffer, 3 µL of 25 mM MgCl_2_, 1 µL of 10 mM dNTP mix, 0.5 µL of each primer (100 pmol/µL), 0.25 µL of Taq DNA polymerase (5 U/µL), and 3 µL of extracted template DNA, with nuclease-free water added to reach the final volume. The conditions used for the amplification of this region were initial denaturation at 95 °C for 5 min, followed by 42 cycles of denaturation at 94°C for 30 s, primer annealing at 62 °C for 30 s, chain elongation at 72 °C for 45 s, and holding at 4°C. The amplicons were then held at 4 °C for subsequent electrophoresis [14].

#### 2.3.3. Determining INH Resistance

The *katG* and *inhA* gene regions were amplified using the primer pairs TB86-TB87 and TB92-TB93, respectively [15]. The standard PCR reaction for the gene region was performed in a final volume of 50 µL, containing 5 µL of 10× PCR buffer, 3 µL of 25 mM MgCl_2_, 1 µL of 10 mM dNTP mix, 0.5 µL of each primer (100 pmol/µL), 0.25 µL of Taq DNA polymerase (5 U/µL), and 3 µL of extracted template DNA, with nuclease-free water added to reach the final volume. The conditions used for the amplification of both regions were initial denaturation at 95 °C for 5 min, followed by 35 cycles of denaturation at 94 °C for 30 s, primer annealing at 54 °C (*katG*) and 64 °C (*inhA*) for 30 s, chain elongation at 72 °C for 45 s, and holding at 4 °C. The amplicons were then held at 4 °C for subsequent electrophoresis.

#### 2.3.4. Determining RIF Resistance

The *rpoB* gene region was amplified using the primer pair TR9-TR8 [15]. The standard PCR reaction for the gene region was performed in a final volume of 50 µL, containing 5 µL of 10× PCR buffer, 3 µL of 25 mM MgCl_2_, 1 µL of 10 mM dNTP mix, 0.5 µL of each primer (100 pmol/µL), 0.25 µL of Taq DNA polymerase (5 U/µL), and 3 µL of extracted template DNA, with nuclease-free water added to reach the final volume. The conditions used for the amplification of the gene region were initial denaturation at 95 °C for 5 min, followed by 35 cycles of denaturation at 94 °C for 30 s, primer annealing at 62 °C for 30 s, chain elongation at 72 °C for 45 s, and holding at 4 °C. The amplicons were then held at 4 °C for subsequent electrophoresis. 

#### 2.3.5. Determining EMB Resistance

The *embB* gene region was amplified using the embB1 primer [16]. The standard PCR reaction for the gene region was performed in a final volume of 50 µL, containing 5 µL of 10× PCR buffer, 3 µL of 25 mM MgCl_2_, 1 µL of 10 mM dNTP mix, 0.5 µL of each primer (100 pmol/µL), 0.25 µL of Taq DNA polymerase (5 U/µL), and 5 µL of extracted template DNA, with nuclease-free water added to reach the final volume. The conditions used for the amplification of the gene regions were initial denaturation at 95 °C for 7 min, followed by 35 cycles of denaturation at 95 °C for 1 min, primer annealing at 63 °C for 1 min, chain elongation at 72 °C for 5 min, and holding at 4 °C. The amplicons were then held at 4 °C for subsequent electrophoresis.

#### 2.3.6. Agarose Gel Electrophoresis

The amplicons obtained after PCR were subjected to electrophoresis on a 1% agarose gel with ethidium bromide at 120 volts for 30 min. After electrophoresis, the gel was imaged with an imaging system (Vilber Lourmat Marne La Vallée, Collegien, France). A 100 bp marker (Thermo Scientific™ Gene Ruler 100 bp DNA Ladder, Thermo Fisher Scientific Inc., 168 Third Avenue, Waltham, MA, USA) was used to determine the band size. The visualization of DNA bands of the lengths presented in Table 1 was considered a positive result (Table 1).

#### 2.3.7. DNA Sequence Analysis

The specific sequences belonging to the relevant gene regions obtained were examined through DNA sequence analysis. After the gene regions responsible for drug resistance were amplified using PCR, the resulting specific sequences were subjected to “Cycle Sequence” PCR using appropriate primers for the sense and antisense strands, utilizing the “BigDye Terminator v3.1 Cycle Sequencing Kit” (Applied Biosystems, Foster City, CA, USA), which contains labeled dideoxynucleotides.

#### 2.3.8. Analysis of Sequence Analysis Data

In the CLUSTAL X (Version 1.83) program, both strands that had undergone sequence analysis were aligned by matching them against each other. Subsequently, the final consensus sequence was recorded in a DNA sequence analysis program such as GENDOC (version 2.6.002) after the unaligned sequences at the ends were trimmed. These sequenced gene regions from each clinical isolate were compared with the reference *M. tuberculosis* H37Rv sequence data published in the PubMed GenBank database to determine specific nucleotide changes and other possible polymorphic regions in the gene region responsible for drug resistance.

## 3. Results

Twenty three MTBC isolates that were found to be resistant to at least two first-line anti-TB drugs using the BACTEC MGIT 960 system were included in this study. Of these isolates, seven (30.5%) were obtained from female patients and sixteen (69.5%) were obtained from male patients. A total of Twenty-two (95.6%) of the cases were pulmonary TB, while one (4.3%) was extrapulmonary. The number of male and female patients, the average age of the patients, and the site of disease involvement are provided in Table 2.

Of the 23 clinical isolates, 18 (78.3%) were resistant to two drugs and five (21.7%) were resistant to three drugs. Furthermore, five (21.7%) of these clinical isolates were identified as MDR-TB (Table 3). 

**Figure 1:**
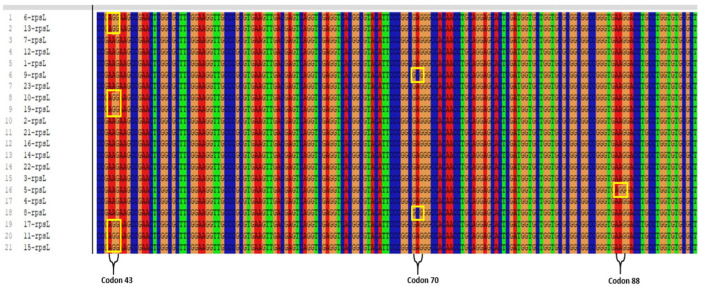
Image of polymorphic regions showing AAG → AGG conversion at codon 43, GAG → GCG at codon 70, and AAG → AGG conversion at codon 88 of the *rpsL* gene region responsible for SM resistance.

Genetic resistance was detected in 10 of the 21 clinical isolates phenotypically resistant to SM. Of the ten patients in whom resistance was detected, six were male and four were female, and all were diagnosed with pulmonary TB. Eight of the patients were of Turkish nationality, while two were of Russian nationality. Phenotypic drug susceptibility testing detected resistance to SM+INH in seven patients, SM+INH+RIF in two patients, and SM+RIF+EMB in one patient. In seven of these isolates, an AAG → AGG (Lysine [Lys] → Arginine [Arg]) substitution was detected at codon 43 of the *rpsL* gene region, which is the most frequently mutated site, and in one isolate an AAG → AGG (Lys → Arg) mutation was detected at codon 88 of the same gene region. Furthermore, a point mutation in the form of a GAG → GCG (Glutamate [Glu] → Alanine [Ala]) substitution was determined at codon 70 of the *rpsL* gene in two isolates, which is a new region not previously reported in the literature [17] (Figure 1) (see Supplementary Materials, Figure S1). Both patients were of Turkish nationality, and it was determined that they were diagnosed with TB within the same year. Phenotypic drug susceptibility testing detected resistance to SM+INH in both patients. While both isolates harbored mutations within the *inhA* promoter region, the *katG* gene remained wild-type. No mutation was detected in the *rpsL* gene region in the remaining 11 clinical isolates. The *rpsL* gene sequences analyzed in this study are available in GenBank (accession no: PX905871–PX905891). The accession numbers for the novel mutation at codon 70 of the *rpsL* gene are PX905878 and PX905879.

**Figure 2:**
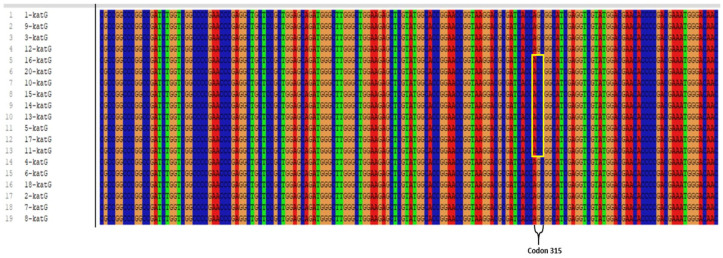
Image of polymorphic regions showing AGC → ACC conversion at codon 315 of the *katG* gene region responsible for INH resistance.

**Figure 3:**
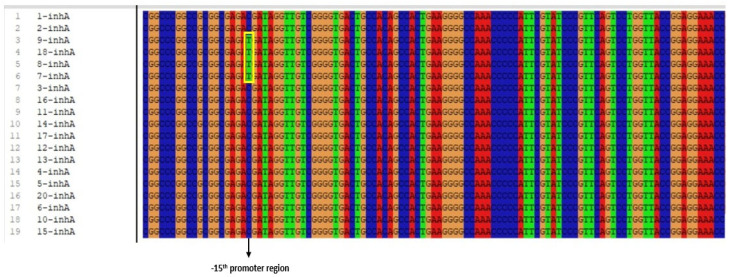
Polymorphic image of the promoter region containing the C → T conversion mutation at position −15 in the *inhA* gene region responsible for INH resistance.

Among the 19 clinical isolates showing INH resistance, mutations were identified in 13 isolates. Of these 13 patients, seven were male and six were female; 12 were diagnosed with pulmonary TB and one with extrapulmonary TB (abscess material). Eleven of the patients were of Turkish nationality, while two were of Russian nationality. Phenotypic drug susceptibility testing detected resistance to SM+INH in nine patients, SM+INH+RIF in three patients, and INH+RIF in one patient. Of the 13 mutation-positive isolates, an AGC → ACC (Serine [Ser] → Threonine [Thr]) substitution was detected at codon 315 of the *katG* gene in 9 isolates (Figure 2) (see Supplementary Materials, Figure S1), which is the most frequently mutated site, and a C → T mutation was detected at the −15 position of the *inhA* gene region in four isolates (Figure 3) (see Supplementary Materials, Figure S1). No mutations were detected in the *katG* and *inhA* gene regions in the remaining six clinical isolates. The gene sequence accession numbers are PX905833–PX905851 for *inhA* and PX905852–PX905870 for *katG*.

**Figure 4:**
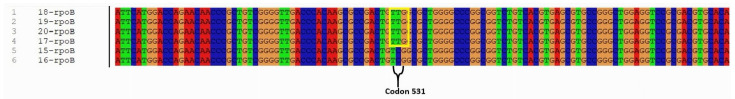
Image of polymorphic regions showing TCG → TTG conversion at codon 531 of the *rpoB* gene region responsible for RIF resistance.

Mutations were detected in four out of six clinical isolates that showed phenotypic RIF resistance. Of these four patients, one was male and three were female; all were diagnosed with pulmonary TB. Three of the patients were of Turkish nationality, while one was of Russian nationality. Phenotypic drug susceptibility testing detected resistance to SM+INH+RIF in two patients, SM+RIF+EMB in one patient, and INH+RIF in one patient. A mutation in the form of a TCG → TTG (Ser → Leucine [Leu]) substitution was detected at codon 531 of the *rpoB* gene region, which is the most frequently encountered site, in four of the six clinical isolates resistant to RIF (Figure 4) (see Supplementary Materials, Figure S1). No mutation was detected in the *rpoB* gene region in the remaining two clinical isolates. The gene sequence accession numbers for *rpoB* are PX905827–PX905832.

**Figure 5:**
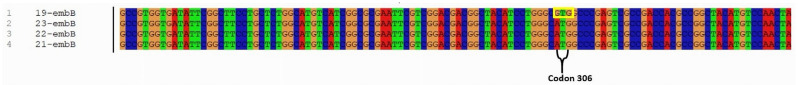
Image of polymorphic regions showing ATG → GTG conversion at codon 306 of the *embB* gene region responsible for EMB resistance.

In one of the four clinical isolates resistant to EMB, a mutation in the form of an ATG → GTG (Methionine [Met] → Valine [Val]) substitution was detected at codon 306 of the *embB* gene region, which is the most frequently mutated site (Figure 5) (see Supplementary Materials, Figure S1). This patient was a female of Russian nationality and was diagnosed with pulmonary TB. Phenotypic drug susceptibility testing detected resistance to SM+RIF+EMB in the patient. No mutation was detected in the *embB* gene region in the remaining three clinical isolates. The gene sequence accession numbers for *embB* are PX905892–PX905895.

According to the resistance patterns of the clinical isolates included in the study, mutations were detected in the *katG* gene region in five, the *rpsL* gene region in seven, and the *inhA* gene region in three of the fourteen clinical isolates resistant to SM+INH. No mutations were detected in any of the three clinical isolates resistant to SM+EMB. Mutations were determined in the *katG* and *rpoB* gene regions in two clinical isolates resistant to INH+RIF. Furthermore, mutations were detected in the *rpsL* gene region in two, the *inhA* gene region in one, the *katG* gene region in three, and the *rpoB* gene region in two of the four clinical isolates resistant to SM+INH+RIF. In one clinical isolate resistant to SM+INH+EMB, mutations were detected only in the *rpsL* and *embB1* gene regions. Additionally, genotypic resistance was detected in three of the five MDR-TB isolates (Table 4).

## 4. Discussion

The emergence and spread of drug-resistant *M. tuberculosis* strains cause serious difficulties for both treatment regimens and disease control. Specifically, resistance that develops against first-line anti-TB drugs increases the spread of MDR-TB isolates, complicates treatment, and necessitates the use of more toxic, long-term drugs. Early and rapid diagnosis of drug resistance is critical for reducing the spread of resistant isolates, establishing effective treatment regimens, and ensuring global TB control [1,5,18].

This study investigated the gene regions responsible for drug resistance in MTBC clinical isolates resistant to at least two first-line anti-TB drugs and found that the most frequent mutations were located in the *katG* and *rpsL* gene regions. A rare mutation in the *rpsL* gene was detected in one isolate at codon 88. Studies in Türkiye have not found any mutations in codon 88 of the *rpsL* gene. Furthermore, a previously unreported GAG → GCG (Glu → Ala) substitution at codon 70 in the *rpsL* gene region was detected in two clinical isolates. This unique mutation provides a significant contribution to the current knowledge and the literature regarding a potential new molecular mechanism that may contribute to SM resistance. 

Studies conducted in different countries reveal variable mutation rates in the *rpsL* gene. These variations are thought to be due to differences in study application methods, the number of samples examined, regional isolates, and antibiotic use profiles. The literature reports that *rpsL*-mediated resistance is most commonly caused by mutations in codon 43 (Lys → Arg), while mutations in codon 88 are generally less common [19].

Studies conducted in Türkiye and around the world have reported that SM resistance is most commonly associated with mutations in the *rpsL* gene region [20,21,22,23]. Studies in various global populations have shown that the mutation frequency at codon 43 of the *rpsL* gene varies widely, ranging from 89.1% to 13.2% [24,25,26,27,28,29]. At codon 88, which shows fewer mutations, mutation rates have been reported to range from 16.7% to 6.6% [26,27,28,29]. Among the 21 clinical isolates examined, 7 exhibited a mutation in the most frequently mutated region of the *rpsL* gene, with an AAG → AGG (Lys → Arg) substitution at codon 43, whereas 1 isolate exhibited a mutation in the same gene region with an AAG → AGG (Lys → Arg) substitution at codon 88. In seven clinical isolates with mutations in codon 43 of the *rpsL* gene region, phenotypic resistance to SM+INH was detected in four, to SM+RIF+EMB in one, and to SM+INH+RIF in two isolates. 

Similarly, only one clinical isolate was found to be resistant at codon 88 of the *rpsL* gene, exhibiting phenotypic resistance to SM+INH. These findings suggest that mutations at codons 43 and 88 of the *rpsL* gene are strongly correlated with phenotypic resistance patterns, implicating this region as a pivotal component in the molecular basis of SM resistance. Our results align with the existing literature, which predominantly identifies codon 43 substitutions as primary drivers of such resistance. 

Since the *rpsL* gene encodes the ribosomal protein S12, amino acid alterations in this domain are hypothesized to cause functional impairment in drug ribosome binding, thereby facilitating resistance. Consequently, these specific codons represent high-priority targets for the molecular diagnosis of SM resistance [27]. 

Furthermore, while the L43R mutation in the *rpsL* gene emerged as the dominant variant, the L88R mutation was observed only sporadically. The prevalence of L43R may reflect a selective advantage that enhances bacterial survival and adaptation under SM-induced pressure; conversely, the rarity of the L88R mutation suggests a more constrained contribution to the overall resistance profile. These observations indicate that certain *rpsL* mutations may have acquired an evolutionary edge due to their specific impact on drug–target affinity, allowing for resistance without compromising biological fitness [5].

The discovery of two isolates harboring the GAG → GCG (Glu → Ala) mutation at codon 70 of the *rpsL* gene, both submitted to the TB laboratory within just a month of each other, strongly points toward a potential epidemiological link rather than a mere coincidence. While a clonal relationship between these isolates remains a distinct possibility, such a connection must be validated through advanced molecular typing techniques (such as MIRU-VNTR, spoligotyping, or whole-genome sequencing) [30]. Although no direct molecular correlation has been established between *inhA* mutations and *rpsL* resistance, the co-existence of *inhA*-mediated INH resistance in strains carrying the *rpsL* codon 70 mutation suggests a cumulative acquisition of multiple resistance mechanisms within these specific isolates. One possible explanation for the observed mutations in the *rpsL* gene is the frequent use of SM in local TB treatment regimens, which may exert selective pressure and contribute to the emergence of these resistance-associated mutations [3].

In MDR-TB isolates and extensively drug-resistant MTBC clinical isolates, it was emphasized that a rare AAG → AGG change was observed in codon 88 of the *rpsL* gene in all clinical isolates and that this change created a mutation on the surface of the protein via Lys → Arg conversion [31]. In our study, phenotypic resistance to the SM+INH+RIF combination was identified in two of the ten clinical isolates harboring mutations in the *rpsL* gene region. The isolate with a mutation at codon 88 of the *rpsL* gene was not classified as MDR, but instead displayed an SM+INH resistance profile. Codon 88 mutation is not always associated with MDR and in some cases can lead to resistance to different drugs. This suggests that resistance profiles are influenced not only by the presence of the mutation but also by local treatment practices (selective imprinting) or co-occurring mutations [3]. 

According to the literature data, the Met → Val substitution at codon 306 in the *embB* gene, which is the region where mutations are most frequently reported, is a common target in research [32,33,34]. Although EMB aims to limit the development of resistance as part of multidrug therapy regimens, *embB* mutations can also be found in multidrug-resistant strains. In our study, a Met → Val mutation at codon 306 of the *embB* gene was detected in one of the four clinical isolates resistant to EMB (Table 4). This co-occurrence (though observed in only a single sample) is noteworthy. However, whether it indicates a general association between the *embB* M306V mutation and broader multidrug resistance profiles requires validation in larger-scale studies.

Codon 306 of the *embB* gene lies within a functionally critical region of the arabinosyltransferase involved in arabinogalactan biosynthesis of the mycobacterial cell wall. Mutations in the *embB* gene decrease the binding affinity of EMB to arabinosyltransferase, enabling continued cell wall biosynthesis and thereby constituting the molecular basis of EMB resistance. Furthermore, these changes can occur through differences in a single or limited number of nucleotides; this could lead to evolutionary selection of codon 306 of the *embB* gene under therapeutic pressure, resulting in its frequent appearance in clinical isolates [8,35].

Different studies have reported that the most commonly observed point mutations associated with INH resistance are a Ser → Thr substitution at codon 315 of the *katG* gene and a C → T substitutions at position −15 of the *inhA* gene [36,37,38,39,40]. The S315T mutation in the *katG* gene has been reported to be responsible for 50–95% of INH resistance [3]. The findings in our study are consistent with the literature. Of the 19 phenotypically INH-resistant clinical isolates, 9 had the AGC → ACC (Ser315Thr) mutation at codon 315 of *katG*, and 4 had the C → T mutation at position −15 of the *inhA* gene. These results once again demonstrate that the most common mutations in INH resistance are concentrated particularly in the *katG* and *inhA* gene regions. The most frequently detected mutations associated with INH resistance, namely alterations at codon 315 of the *katG* gene and the C → T transition at position −15 in the promoter region of the *inhA* gene, reduce the efficacy of INH by decreasing its activation and increasing the expression of the target enzyme, respectively [35]. These mutations allow the bacterium to largely preserve its essential metabolic functions and virulence, confer a selective advantage under drug pressure, and consequently facilitate its persistence and widespread dissemination among clinical isolates [41].

In other studies including clinical isolates resistant to INH and RIF, it has been reported that the most frequent point mutation associated with INH resistance is an AGC → ACC substitution at codon 315 of the *katG* gene, followed by a C → T transition at position −15 of the *inhA* gene. For RIF resistance, the most common point mutation has been identified as a TCG → TTG substitution at codon 531 of the *rpoB* gene [15,42,43,44,45,46]. In our study, mutations were observed in 13 of the 19 INH-resistant clinical isolates, while a Ser→Leu mutation was detected at codon 531 of the *rpoB* gene in four of the six RIF-resistant clinical isolates (Table 4). These findings are similar to the results reported in the literature, and similar mutation profiles were identified. In the present study, the most prevalent mutations among RIF- and INH-resistant isolates were identified as S531L in the *rpoB* gene and S315T in the *katG* gene, in agreement with previous findings. In addition, the C15T mutation in the *inhA* gene represents one of the most frequently reported alterations associated with INH resistance. Surveillance of these key resistance-associated mutations (S531L, S315T, and C15T) in local MTBC isolates is therefore essential for strengthening molecular diagnostic approaches and regional epidemiological monitoring. Such efforts may enable the earlier detection of MDR-TB and support the optimization of treatment regimens, thereby limiting the emergence and spread of multidrug- and extensively drug-resistant TB [3,35].

Resistance at the genotypic level was observed in three out of five MDR-TB isolates included in this study. This finding demonstrates that the concordance between phenotypically determined resistance and genotypic results was partially achieved. Of the two INH+RIF-resistant clinical isolates, one had mutations in the *katG* and *rpoB* gene regions, and the other had mutations in the *inhA* and *rpoB* gene regions. Furthermore, of the three SM+INH+RIF-resistant clinical isolates, two had mutations in the *rpsL* gene, three in the *katG* gene, and one in the *rpoB* gene (Table 4). These findings suggest that the development of resistance in MDR-TB isolates is associated with multigenic mutations. The presence of multiple resistance-associated mutations across different gene regions suggests that diagnostic tests targeting only a single gene region may fail to detect certain resistant isolates, potentially reducing test sensitivity and specificity. Identifying gene regions that harbor mutations predominant in the local population may support faster and more accurate clinical decision-making by incorporating these regions into rapid molecular diagnostic assays, thereby facilitating timely and appropriate treatment [47].

In our study, mutations in the most frequently observed resistance genes were investigated in clinical isolates resistant to at least two anti-TB drugs; while this approach provides valuable insights into the molecular basis of multidrug resistance, the focus on only the most common mutations does not capture the potential contribution of rare or previously uncharacterized mutations, nor the possible interactions between different genes.

This study is the first in Türkiye and worldwide to report a previously unidentified mutation (GAG → GCG, G70A) at codon 70 of the *rpsL* gene in clinical isolates of *M. tuberculosis*. The codon 70 mutation in the *rpsL* gene, which encodes the ribosomal protein S12, may induce conformational changes in the 30S ribosomal subunit, potentially affecting SM binding. These changes could indirectly influence ribosomal fidelity, protein synthesis, and drug susceptibility. However, this position has not been functionally characterized previously, and the structural and functional effects of the mutation have not yet been experimentally validated. Furthermore, we identified the rare L88A (AAG → AGG) mutation at codon 88 of the *rpsL* gene, which has not been previously reported in Türkiye. This finding provides novel insights and represents an important contribution to the understanding of the genetic mechanisms underlying drug resistance in *M. tuberculosis*.

The main limitations include the relatively small sample size and the inability to examine all gene regions responsible for the drug resistance. The absence of detectable mutations in the investigated gene regions does not rule out the presence of resistance in other loci; thus, further studies are warranted to examine alternative mutation patterns that may contribute to drug resistance. Our study provides novel information by identifying the previously unreported *rpsL* mutation G70A and characterizing mutational patterns in drug-resistant isolates in this region. These findings highlight the importance of our study and provide a foundation for future research.

## 5. Conclusions

In conclusion, DNA sequencing and genotypic analyses yielded results that are consistent with the previously reported mutation data for first-line anti-TB drugs on a global scale. This study is the first to identify a previously unidentified mutation (GAG → GCG, G70A) at codon 70 of the *rpsL* gene in clinical isolates from Turkey and worldwide. This finding provides a more detailed understanding of the molecular mechanisms of SM resistance, points to the existence of potential novel resistance sites, and offers a potential target for future functional studies and diagnostic marker development. DNA sequencing is utilized not only for the confirmation of known drug resistance-associated gene regions but also for the identification of novel genetic loci that may contribute to resistance, thereby providing a foundation for future molecular studies.

## Figures and Tables

**Table 1:** Gene regions, nucleotide sequences, and amplification characteristics of primers used for PCR amplification and detection of drug-resistant *M. tuberculosis* strains.

Drug	Gene Regions	Nucleotide Sequence (5′-3′)	Annealing Temp (°C)	PCR Product Size (bp)	Reference
SM	*rpsL*	Forward: CCAACCATCCAGCAGCTGGT Reverse: ATCCAGCGAACCGCGGATGA	62	306	[14]
	*rrs* (530)	Forward: GATGACGGCCTTCGGGTTGT Reverse: TCTAGCTGCCCGTATCGCC	62	238	
	*rrs* (912)	Forward: GTAGTCCACGCCGTAAACGG Reverse: AGGCCACAAGGAACGCCTA	62	240	
INH	*katG*	Forward: GAAACAGCGGCGCTGATCGT Reverse: GTTGTCCCATTTCGTCGGGG	54	209	[15]
	*inhA*	Forward: CCTCGCTGCCCAGAAAGGGA Reverse: ATCCCCCGGTTTCCTCCGGT	64	248	
RIF	*rpoB*	Forward: TCGCCGCGATCAAGGAGT Reverse: GTGCACGTCGCGGGACCTCCA	62	158	[15]
EMB	*embB* (*B*1)	Forward: CCGACCACGCTGAAACTGC Reverse: GTAATACCAGCCGAAGGGATCCT	63	364	[16]

SM: Streptomycin; INH: Isoniazid; RIF: Rifampicin; EMB: Ethambutol.

**Table 2:** Number of male and female patients, average ages, and disease location information of the 23 clinical isolates included in the study.

		Disease Location	
	Average Ages	Pulmonary	Extrapulmonary
Male (*n* = 16)	57 (min: 34, max: 79)	15 (65.2%)	1 (4.3%)
Female (*n* = 7)	43 (min: 25, max: 57)	7 (30.5%)	---

**Table 3:** The resistance profiles of 23 isolates determined to be resistant to at least two first-line anti-TB drugs.

First-Line Anti-TB Drug	Resistant
SM+INH	14 (60.9%)
INH+RIF	2 (8.7%)
SM+EMB	3 (13.1%)
SM+RIF+EMB	1 (4.3%)
SM+INH+RIF	3 (13%)
Total	23 (100%)

SM: Streptomycin; INH: Isoniazid; RIF: Rifampicin; EMB: Ethambutol.

**Table 4:** Genetic mutations observed in clinical isolates resistant to at least two of the first-line anti-TB drugs (*rpsL*, *inhA*, *katG*, *rpoB*, and *embB*).

		Nucleotide and Amino Acid Changes				
Resistance Pattern	Number of Isolates	*rpsL*	*inhA*	*katG*	*rpoB*	*embB (B1)*
SM+INH	4 (17.4%)	Wild-type	Wild-type	Wild-type	-	-
	2 (8.7%)	Wild-type	Wild-type	S315T (AGC → ACC)	-	-
	2 (8.7%)	L43A (AAG → AGG)	Wild-type	S315T (AGC → ACC)	-	-
	2 (8.7%)	G70A (GAG → GCG)	−15 C → T	Wild-type	-	-
	2 (8.7%)	L43A (AAG → AGG)	Wild-type	Wild-type	-	-
	1 (4.4%)	Sokak tipi	−15 C → T	Wild-type	-	-
	1 (4.4%)	L88A (AAG → AGG)	Wild-type	S315T (AGC → ACC)	-	-
SM+EMB	3 (13%)	Wild-type	-	-	-	Wild-type
INH+RIF	1 (4.4%)	-	Wild-type	S315T (AGC → ACC)	S531L (TCG → TTG)	-
	1 (4.4%)	-	−15 C → T	Wild-type	S531L (TCG → TTG)	-
SM+INH+RIF	1 (4.4%)	L43A (AAG → AGG)	Wild-type	S315T (AGC → ACC)	Wild-type	-
	1 (4.4%)	Wild-type	Wild-type	S315T (AGC → ACC)	Wild-type	-
	1 (4.4%)	L43A (AAG → AGG)	Wild-type	S315T (AGC → ACC)	S531L (TCG → TTG)	-
SM+RIF+EMB	1 (4.4%)	L43A (AAG → AGG)	-	-	S531L (TCG → TTG)	M306V (ATG → GTG)

SM: Streptomycin; INH: Isoniazid; RIF: Rifampicin; EMB: Ethambutol; S: Serine (Ser); T: Threonine (Thr); L: Lysine (Lys); A: Arginine (Arg); G: Glutamate (Glu); A: Alanine (Ala); L: Leucine (Leu).

## Data Availability

The original contributions presented in this study are included in the article. Further inquiries can be directed to the corresponding author.

## Supplementary Materials

The following supporting information can be downloaded at: https://www.mdpi.com/article/10.3390/pathogens15020222/s1, Figure S1: Chromatogram images of the *rpsL*, *katG*, *inhA*, *rpoB* and *embB* gene regions from the isolates.
