# Supplementary material for: Investigation of Gene Regions Responsible for Drug Resistance in Clinical Isolates of Mycobacterium tuberculosis Complex Resistant to at Least Two First-Line Anti-Tuberculosis Drugs
Source: Pathogens. 2026 Feb 16;15(2):222. doi: 10.3390/pathogens15020222 (PMC12943113; doi:10.3390/pathogens15020222)
Supplement: Supplementary file 1 [file pathogens-15-00222-s001.zip › pathogens-4092661-supplementary.pdf]

Figure S1: Chromatogram images of the *rpsL*, *katG*, *inhA*, *rpoB* and *embB* gene regions from the isolates.

Chromatogram images of the *rpsL* gene region from 21 isolates (A1-A21).

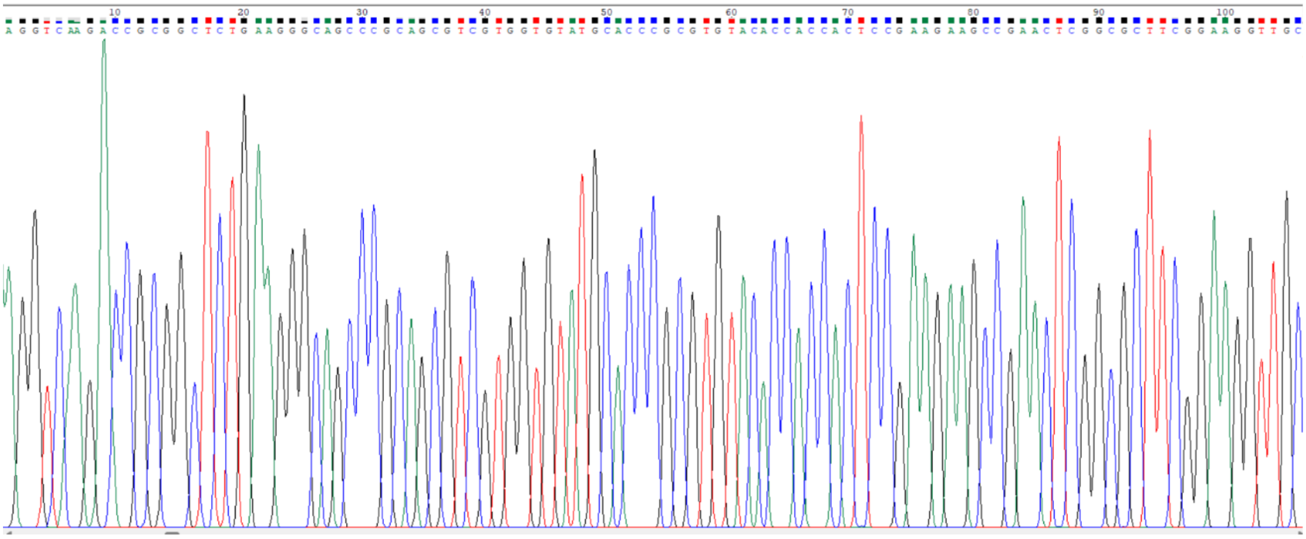

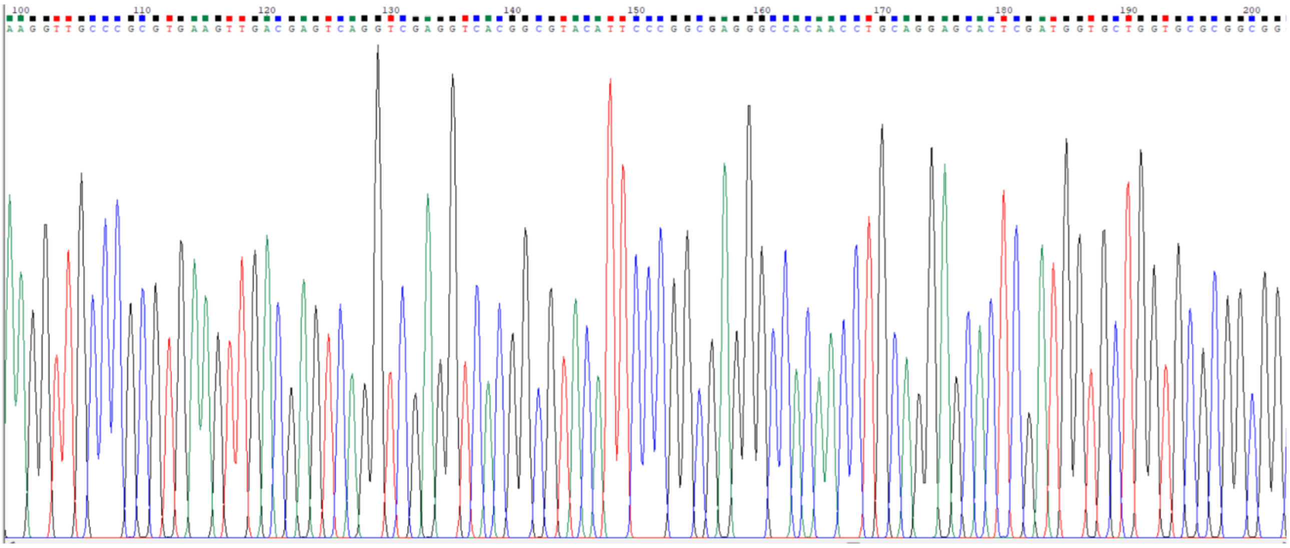

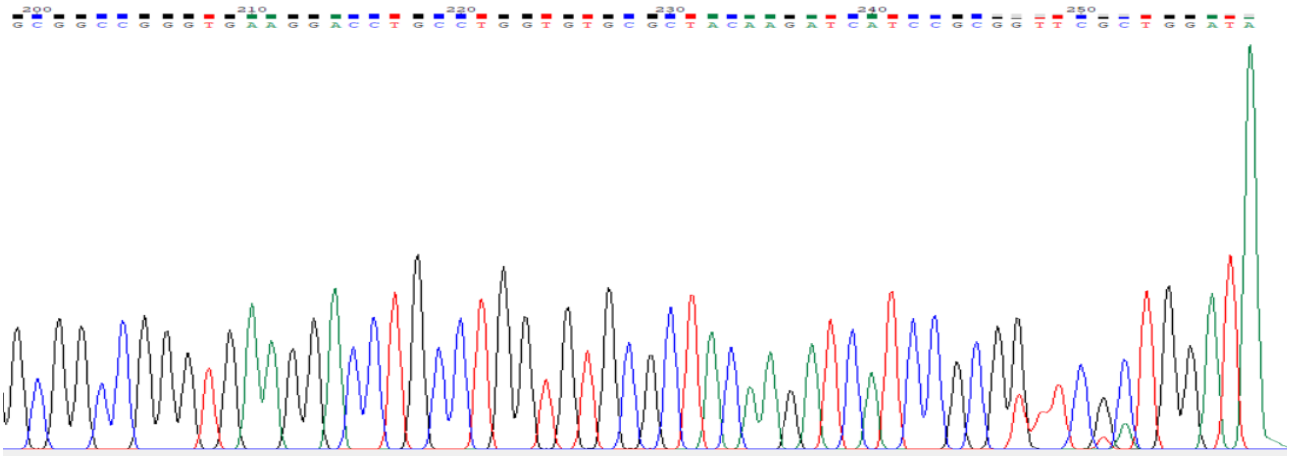

A1. Sequencing chromatograms of the *rpsL* gene, covering nucleotide positions 10–250, show a region with no detected mutations.

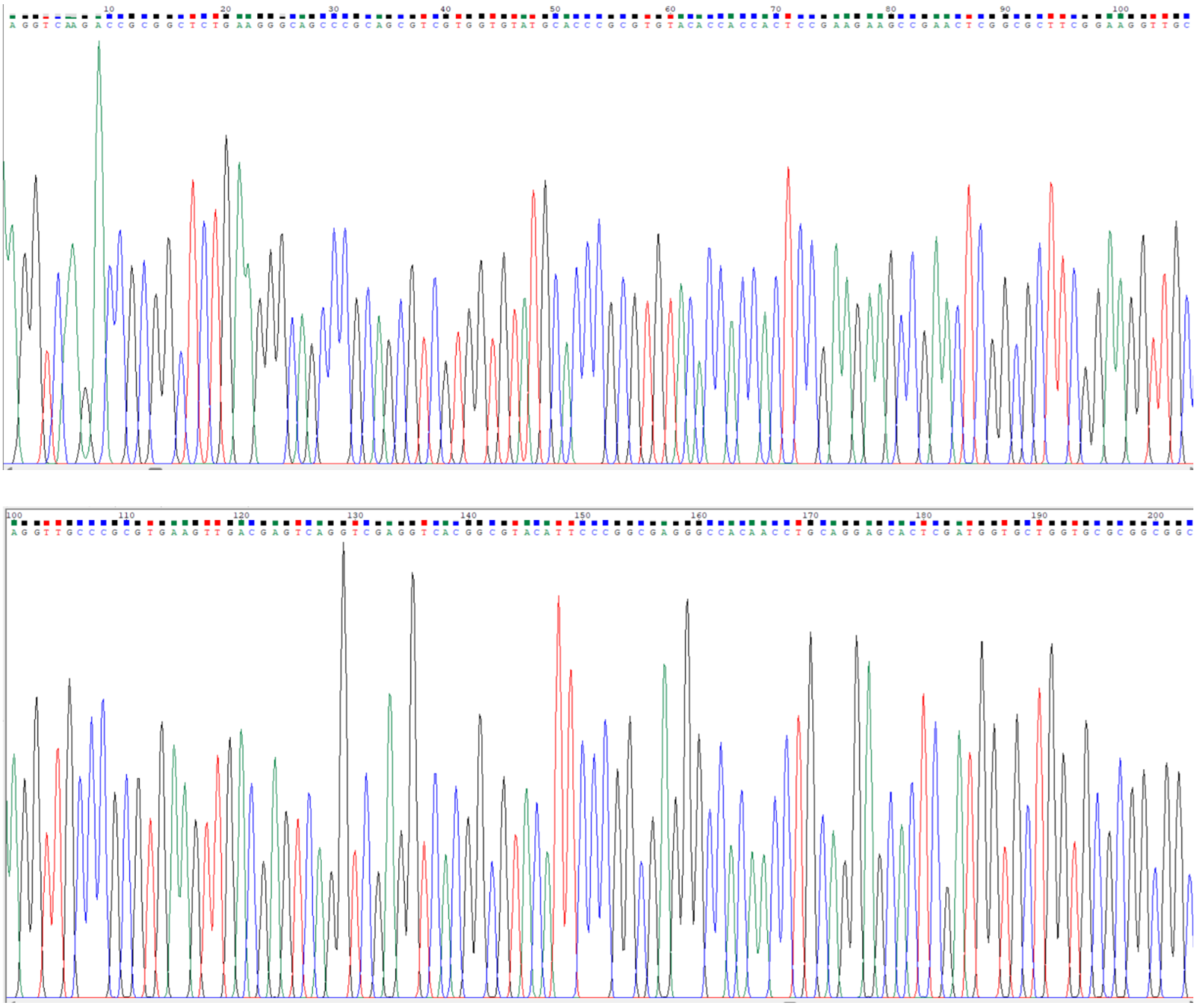

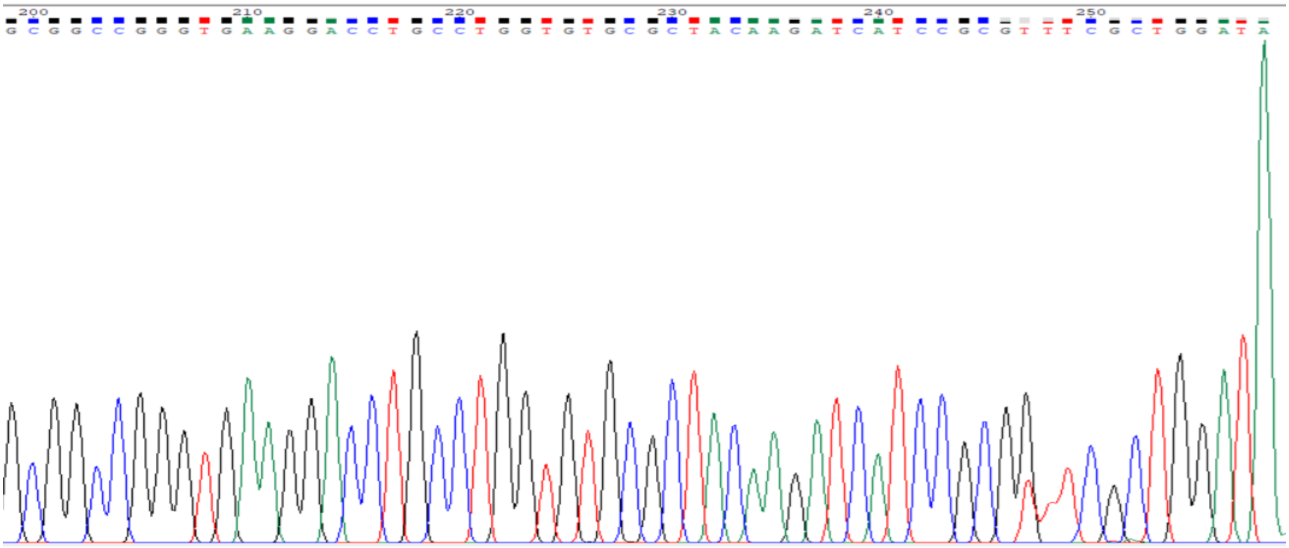

A2. Sequencing chromatograms of the *rpsL* gene, covering nucleotide positions 10–250, show a region with no detected mutations

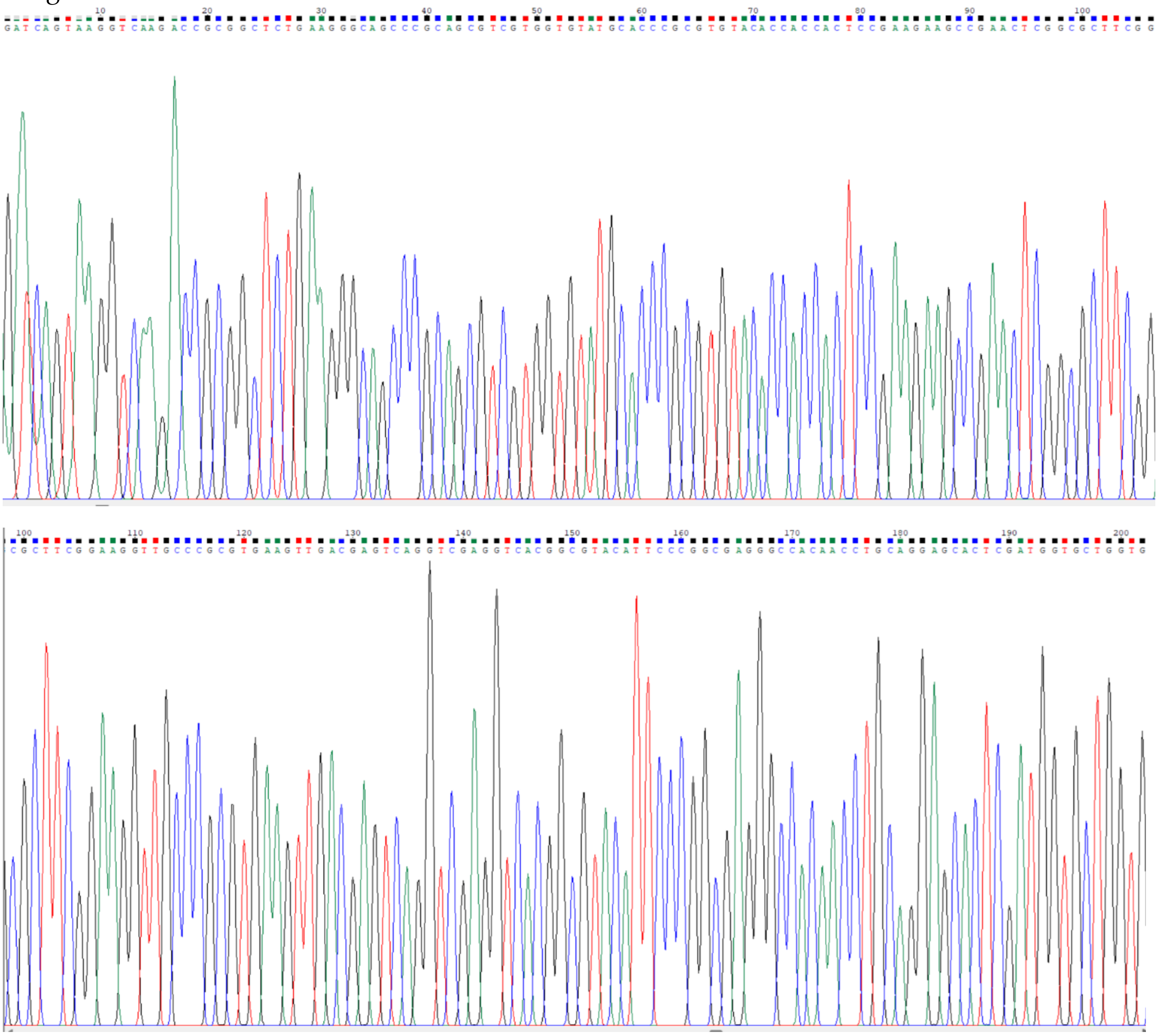

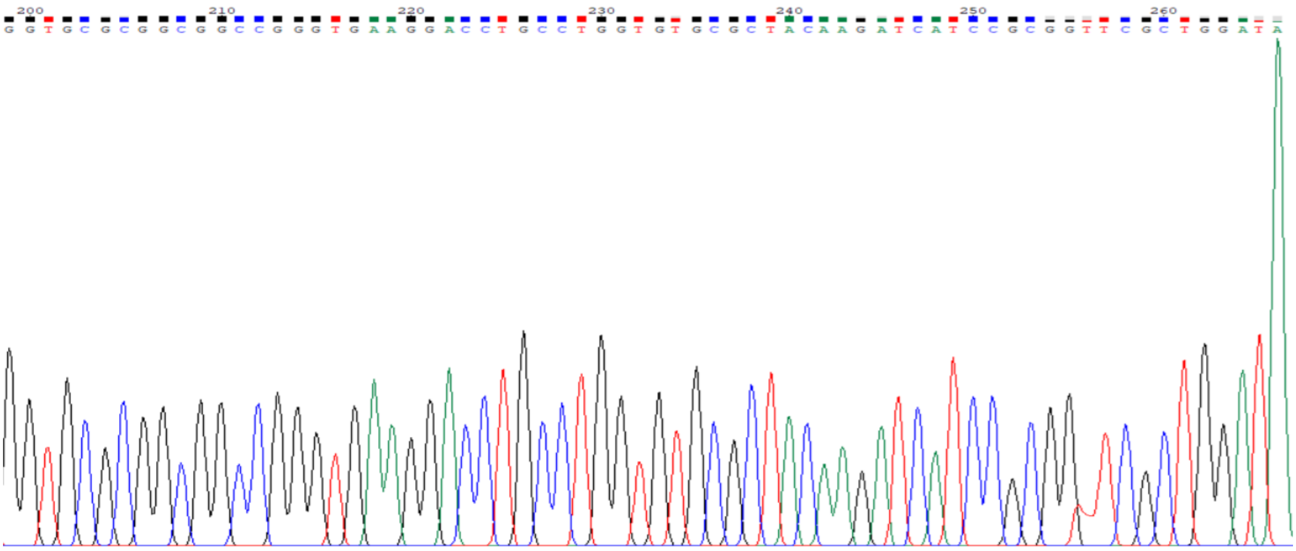

A3. Sequencing chromatograms of the *rpsL* gene, covering nucleotide positions 10–260, show a region with no detected mutations.

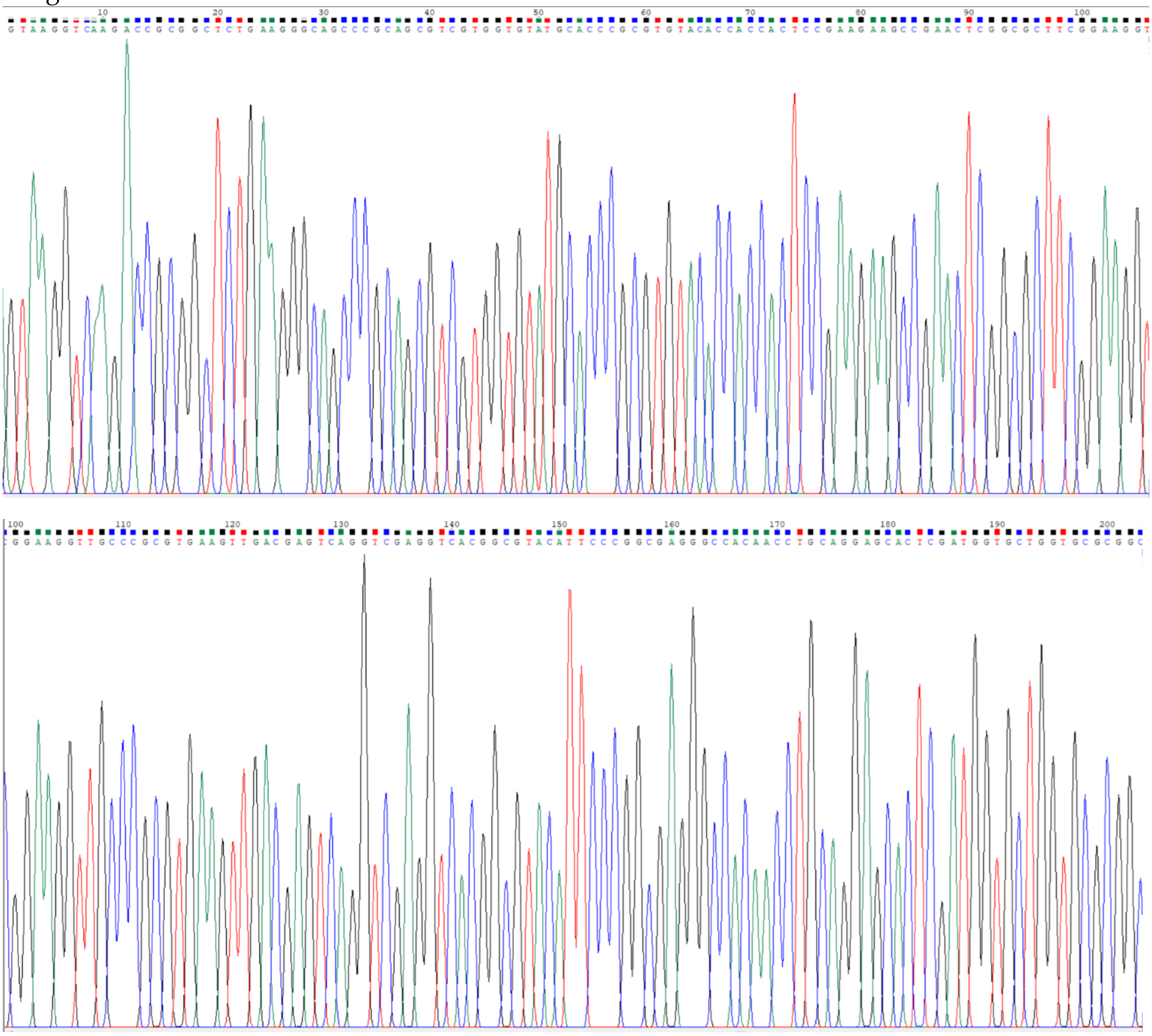

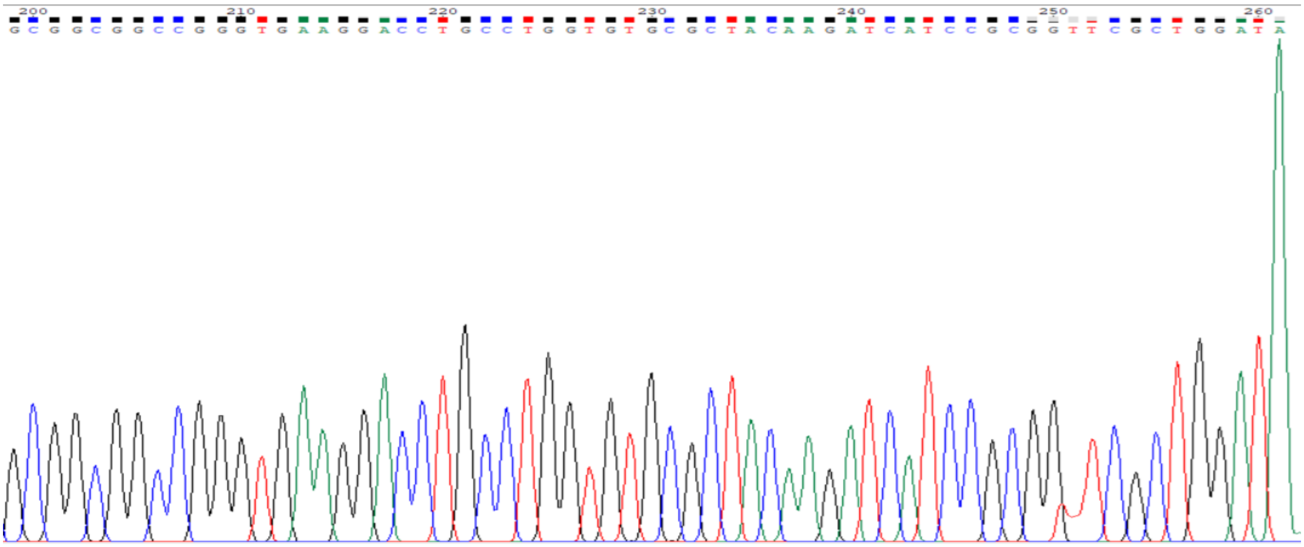

A4. Sequencing chromatograms of the *rpsL* gene, covering nucleotide positions 10–260, show a region with no detected mutations.

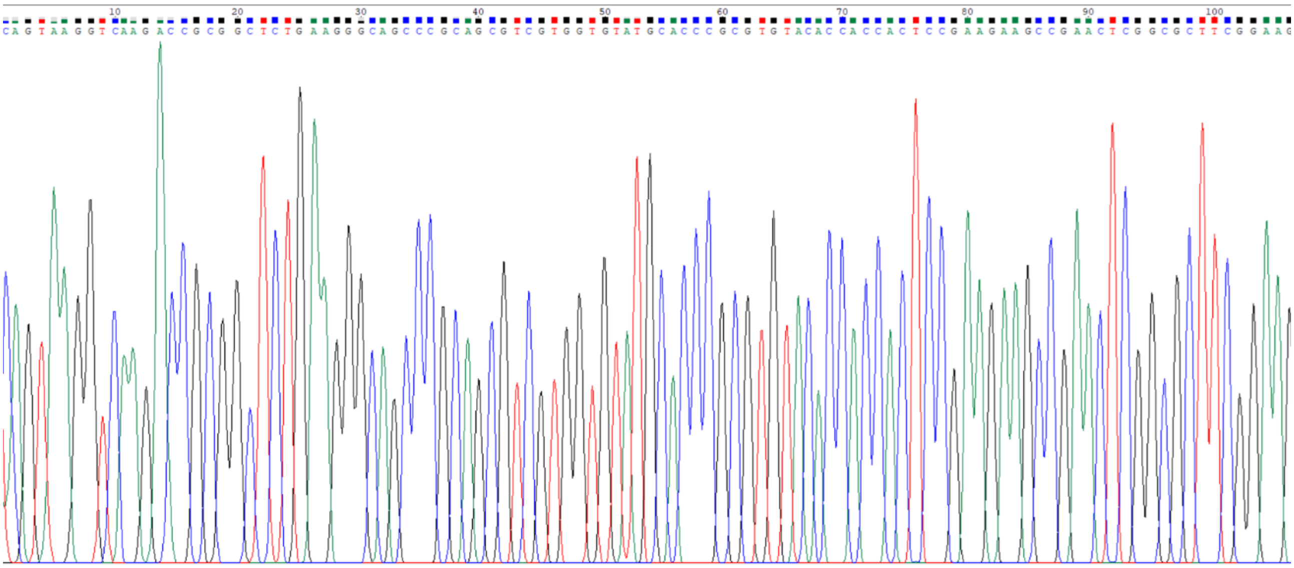

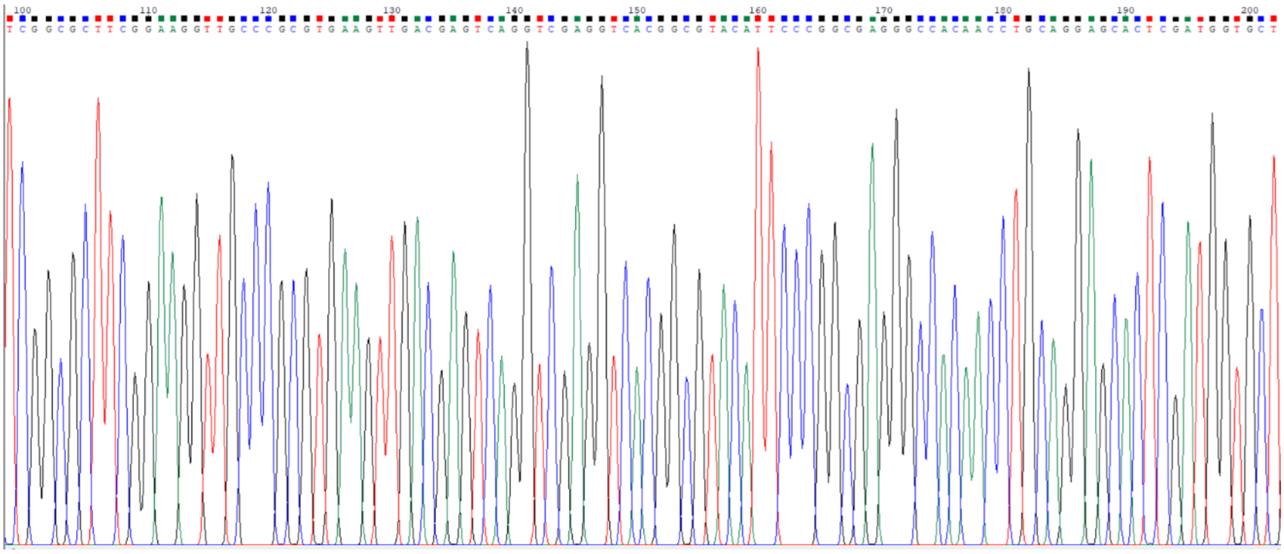

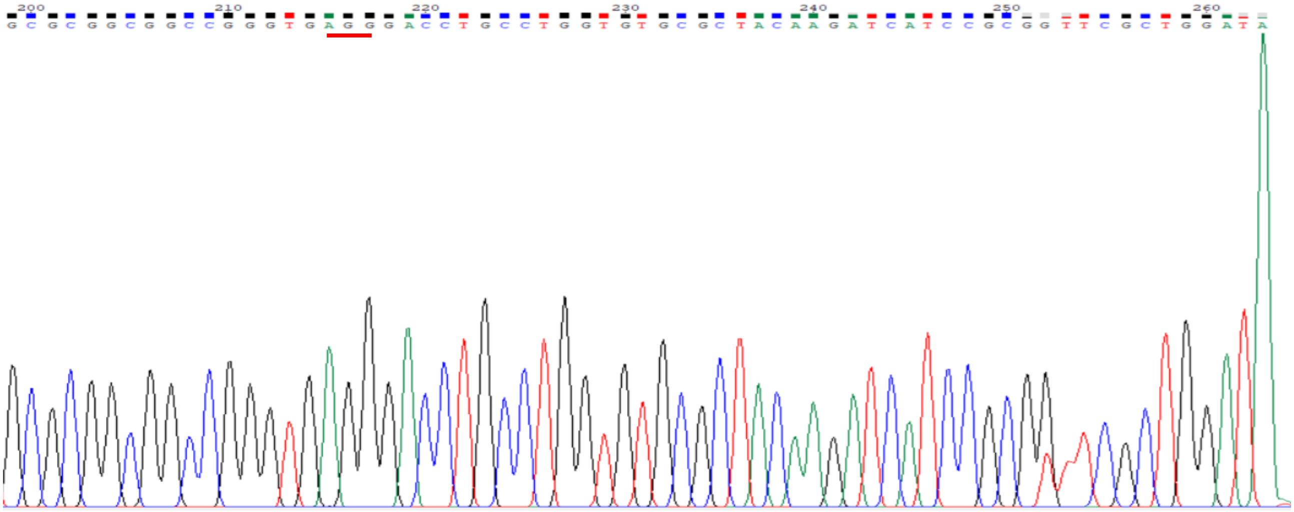

A5. Sequencing chromatograms of the *rpsL* gene, covering nucleotide positions 10–260, show an **AAG→AGG** substitution between nucleotide positions 210–220.

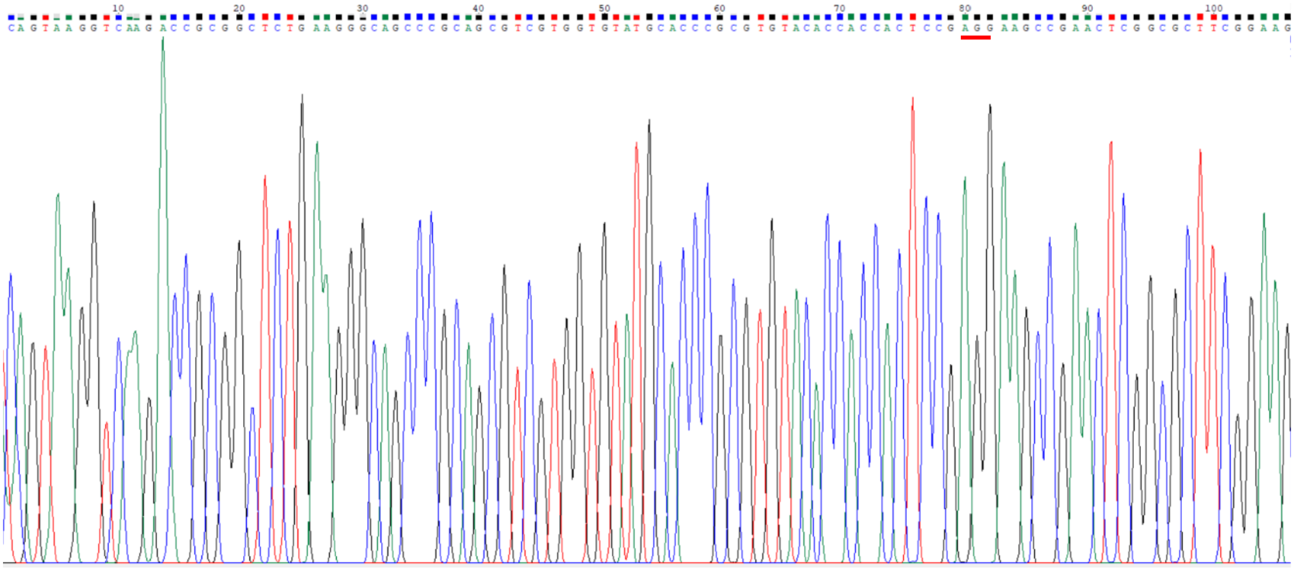

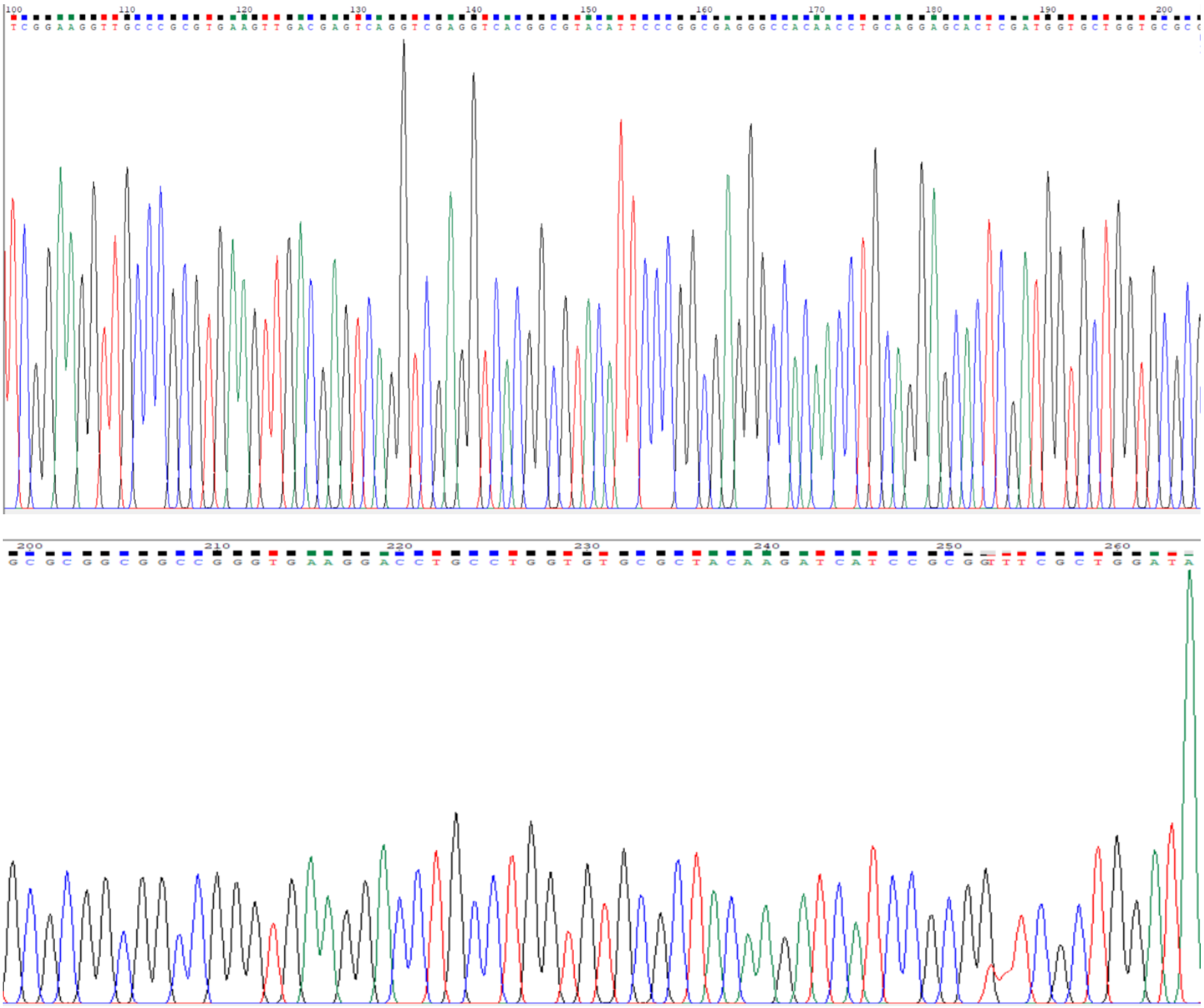

A6. Sequencing chromatograms of the *rpsL* gene, covering nucleotide positions 10–260, show an AAG→AGG substitution between nucleotide positions 80–90.

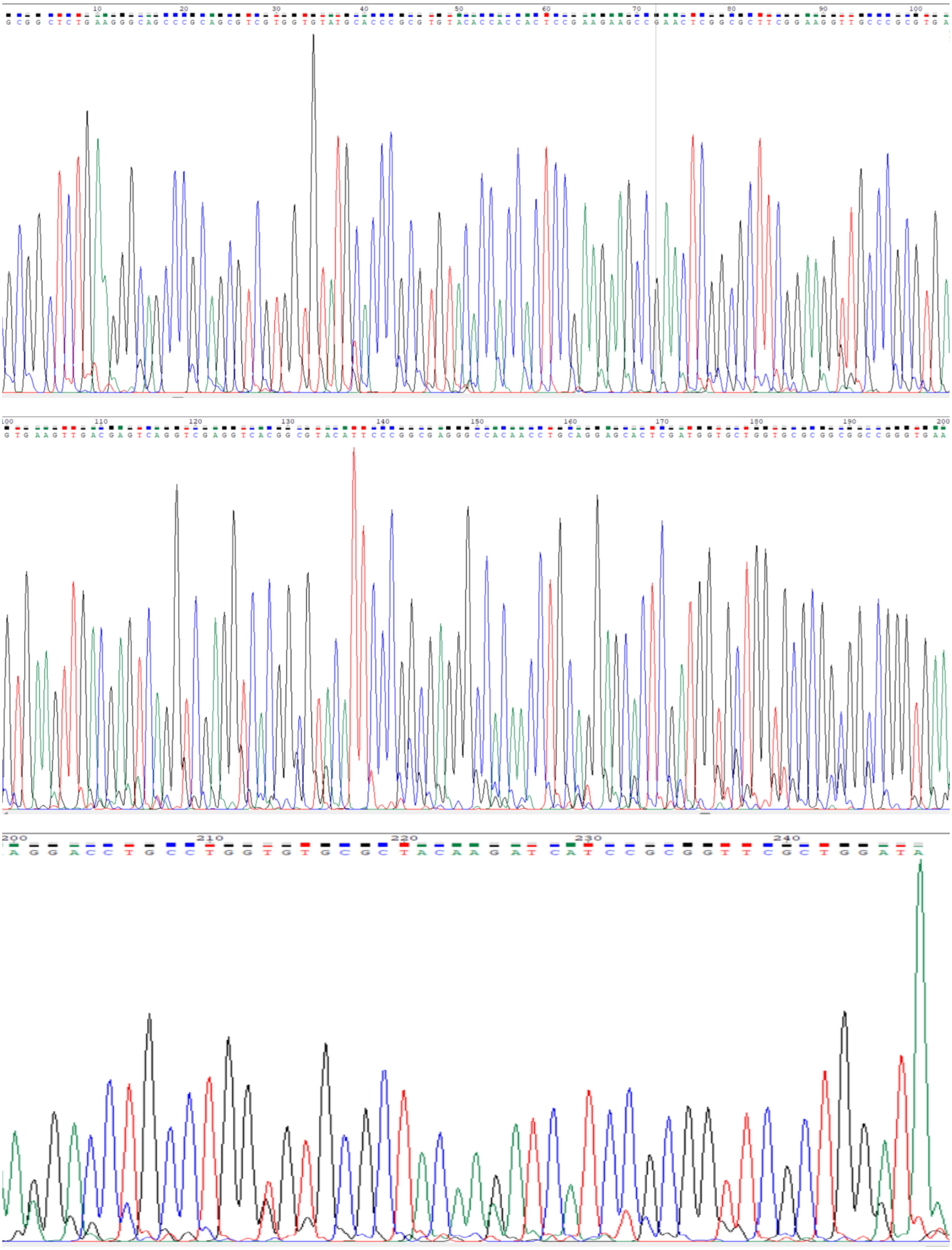

A7. Sequencing chromatograms of the *rpsL* gene, covering nucleotide positions 10–240, show a region with no detected mutations.

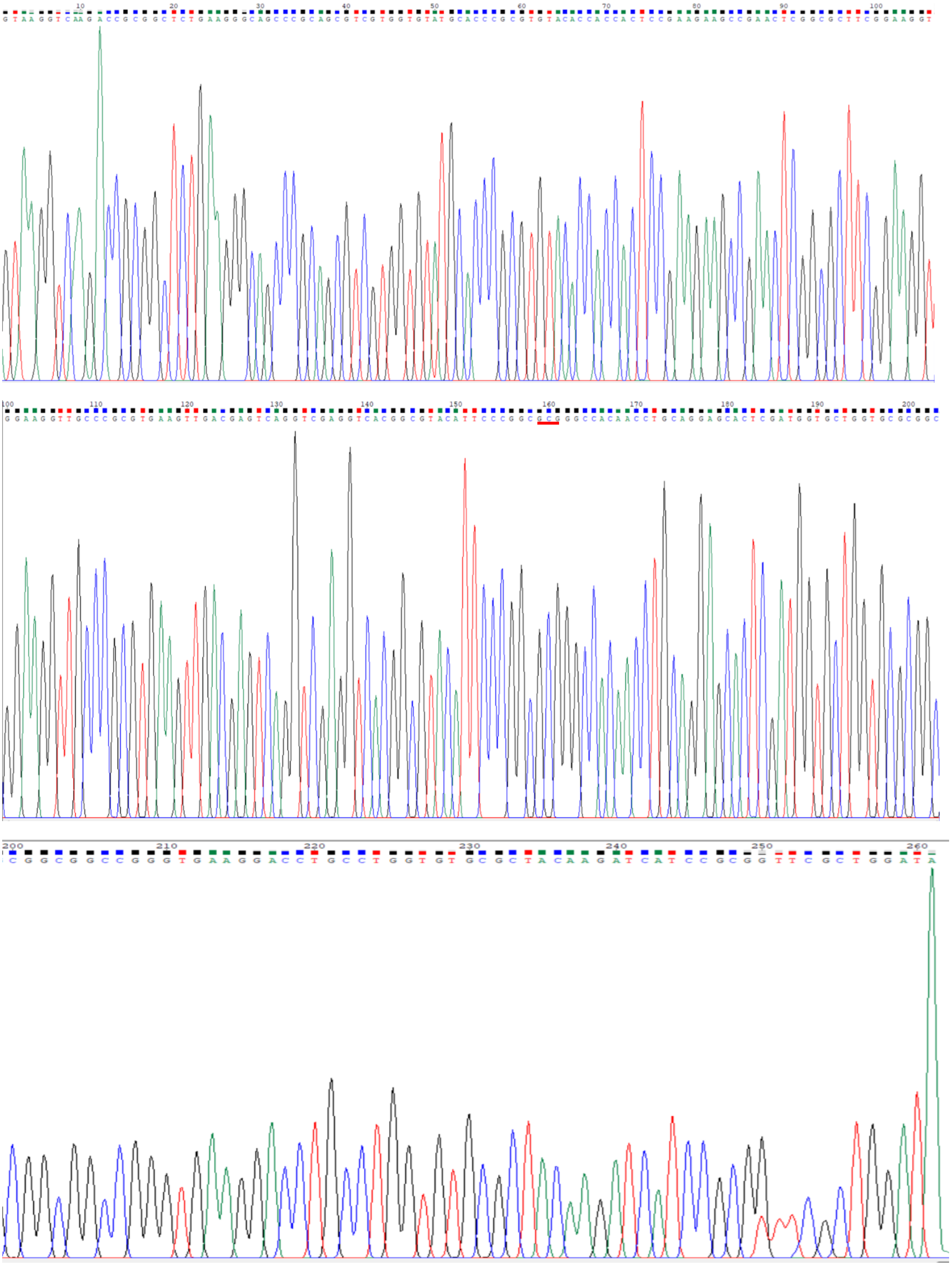

A8. Sequencing chromatograms of the *rpsL* gene, covering nucleotide positions 10–260, show an **GAG→GCG** substitution between nucleotide positions 160.

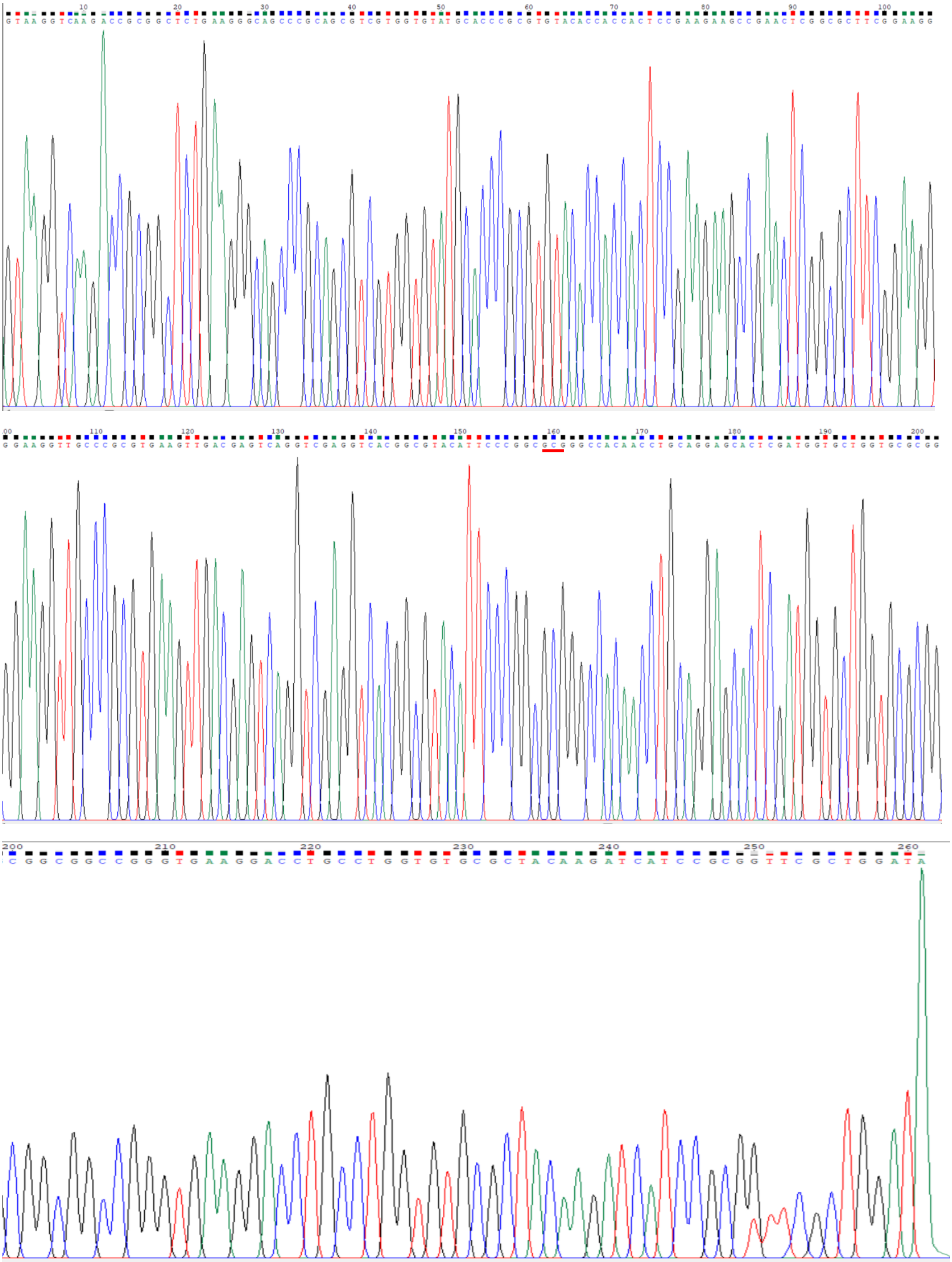

A9. Sequencing chromatograms of the *rpsL* gene, covering nucleotide positions 10–260, show an **GAG→GCG** substitution between nucleotide positions 160.

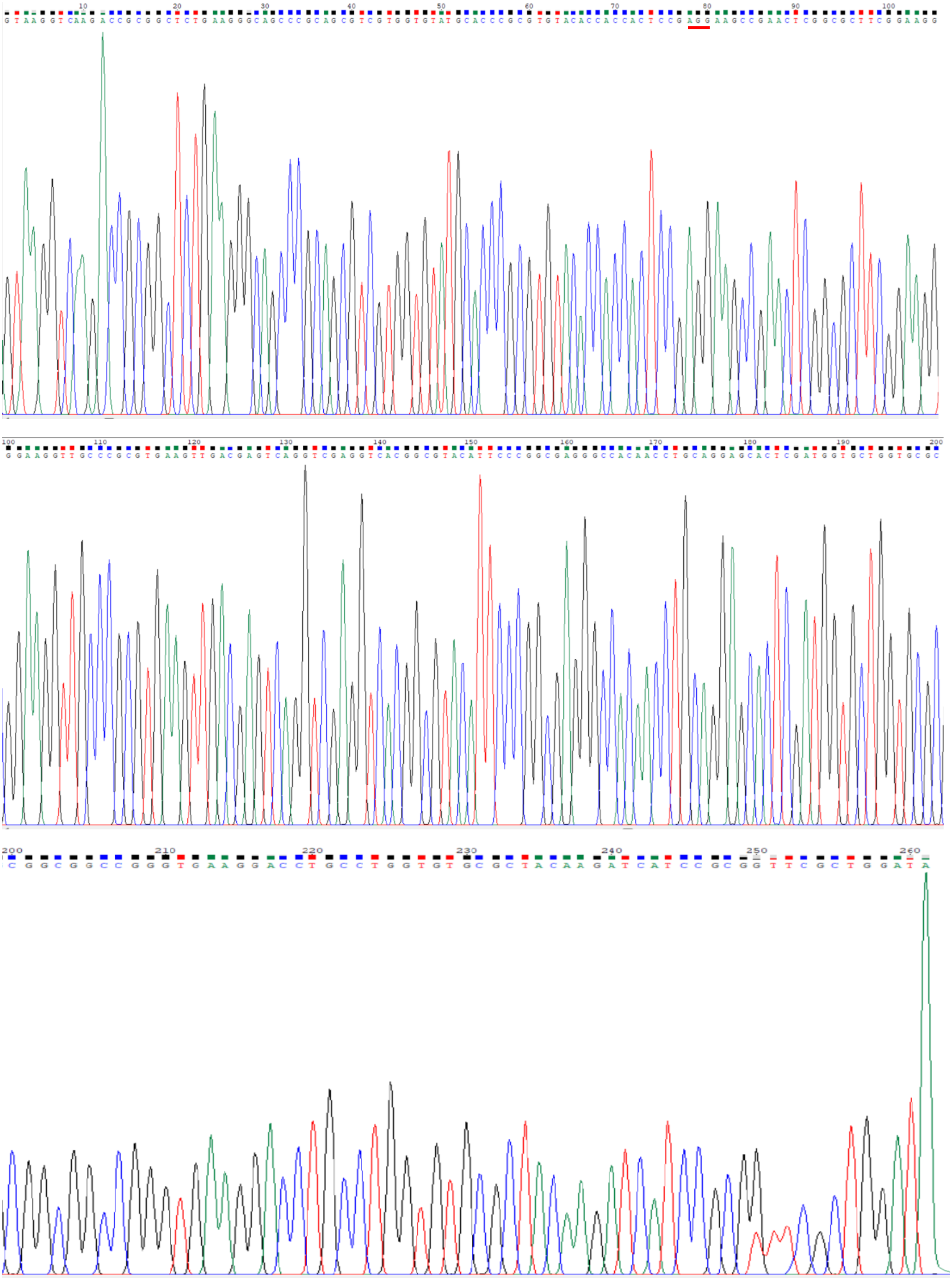

A10. Sequencing chromatograms of the *rpsL* gene, covering nucleotide positions 10–260, show an **AAG→AGG** substitution between nucleotide positions 70–80.

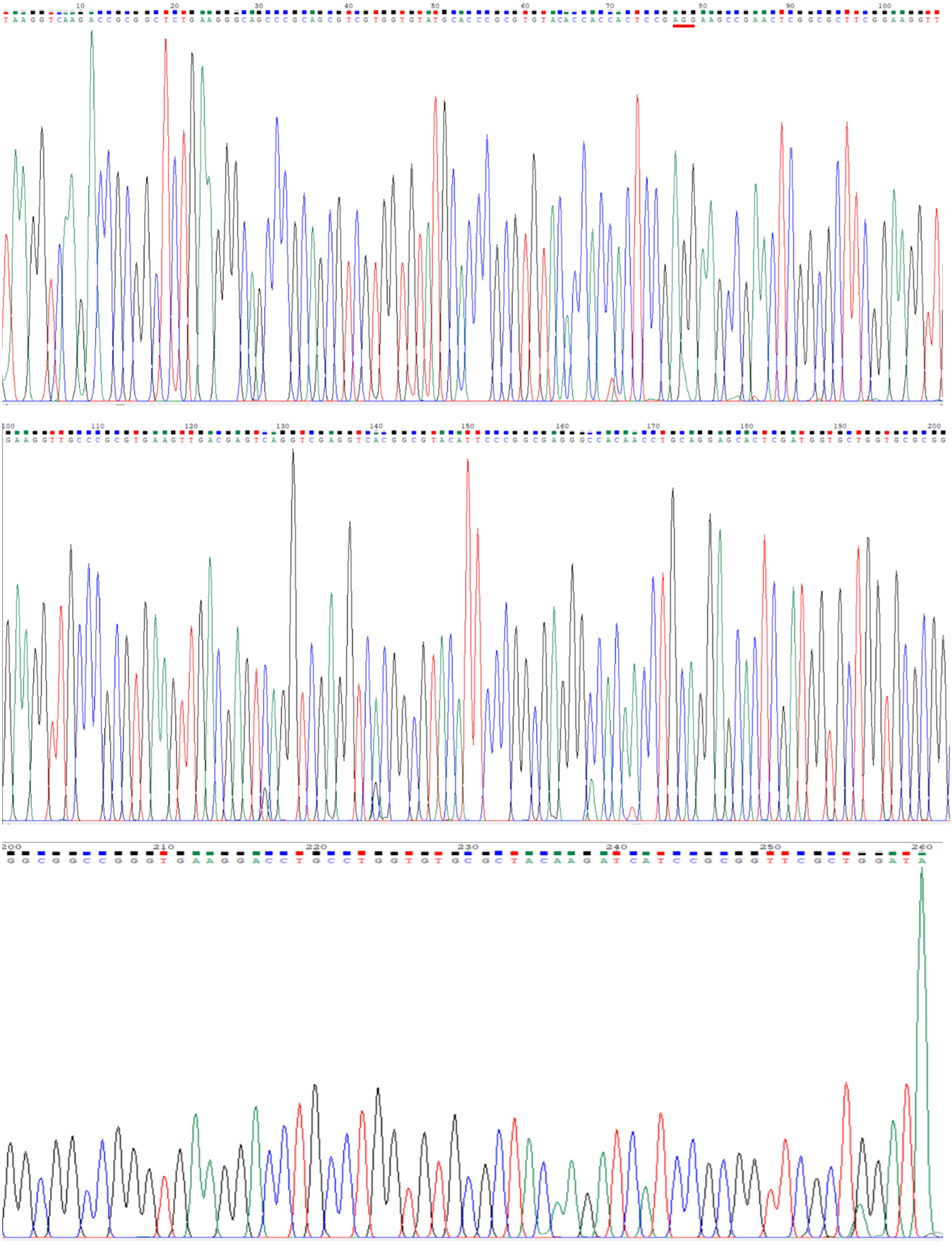

A11. Sequencing chromatograms of the *rpsL* gene, covering nucleotide positions 10–260, show an **AAG→AGG** substitution between nucleotide positions 70–80.

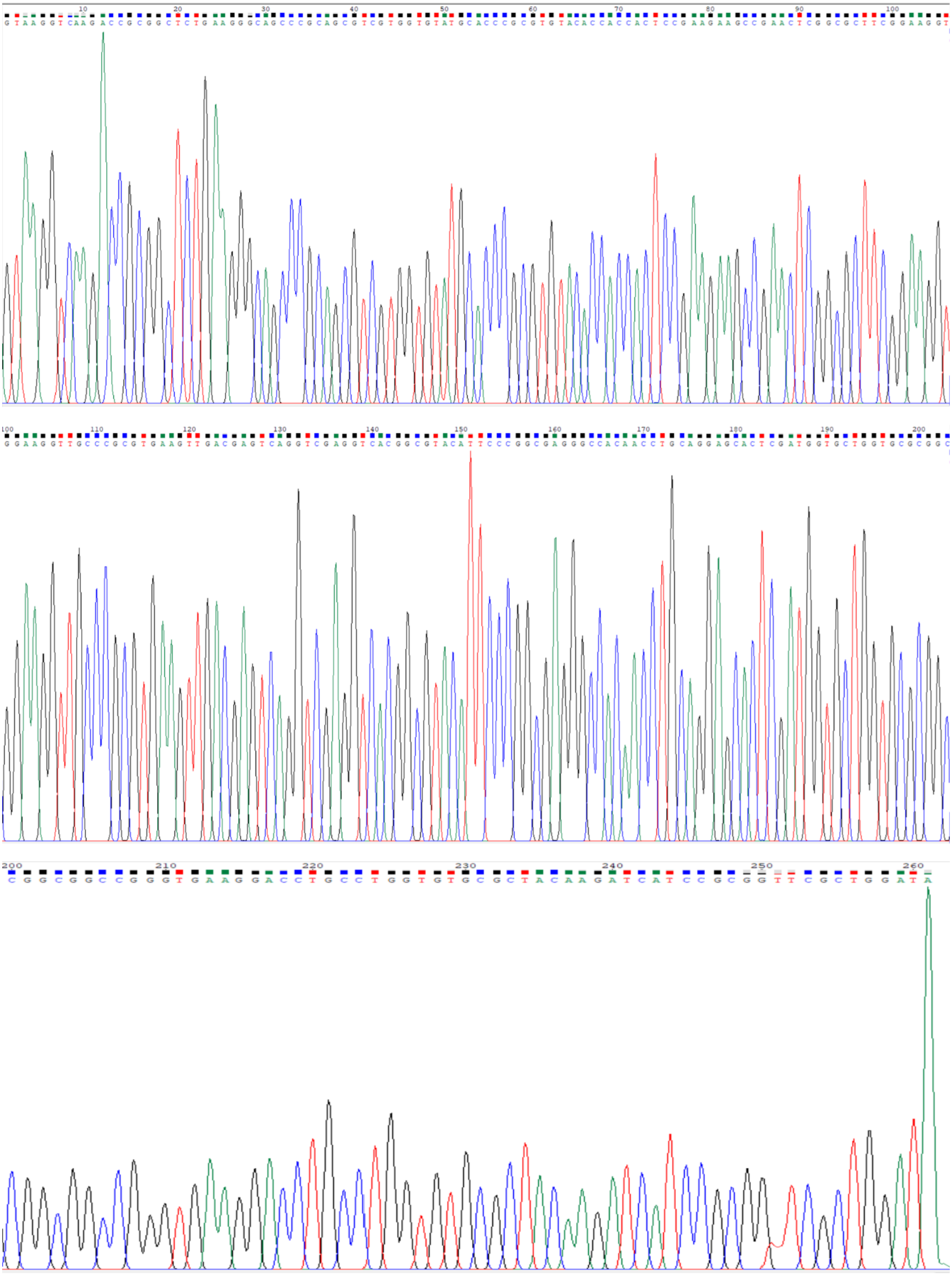

A12. Sequencing chromatograms of the *rpsL* gene, covering nucleotide positions 10–260, show a region with no detected mutations.

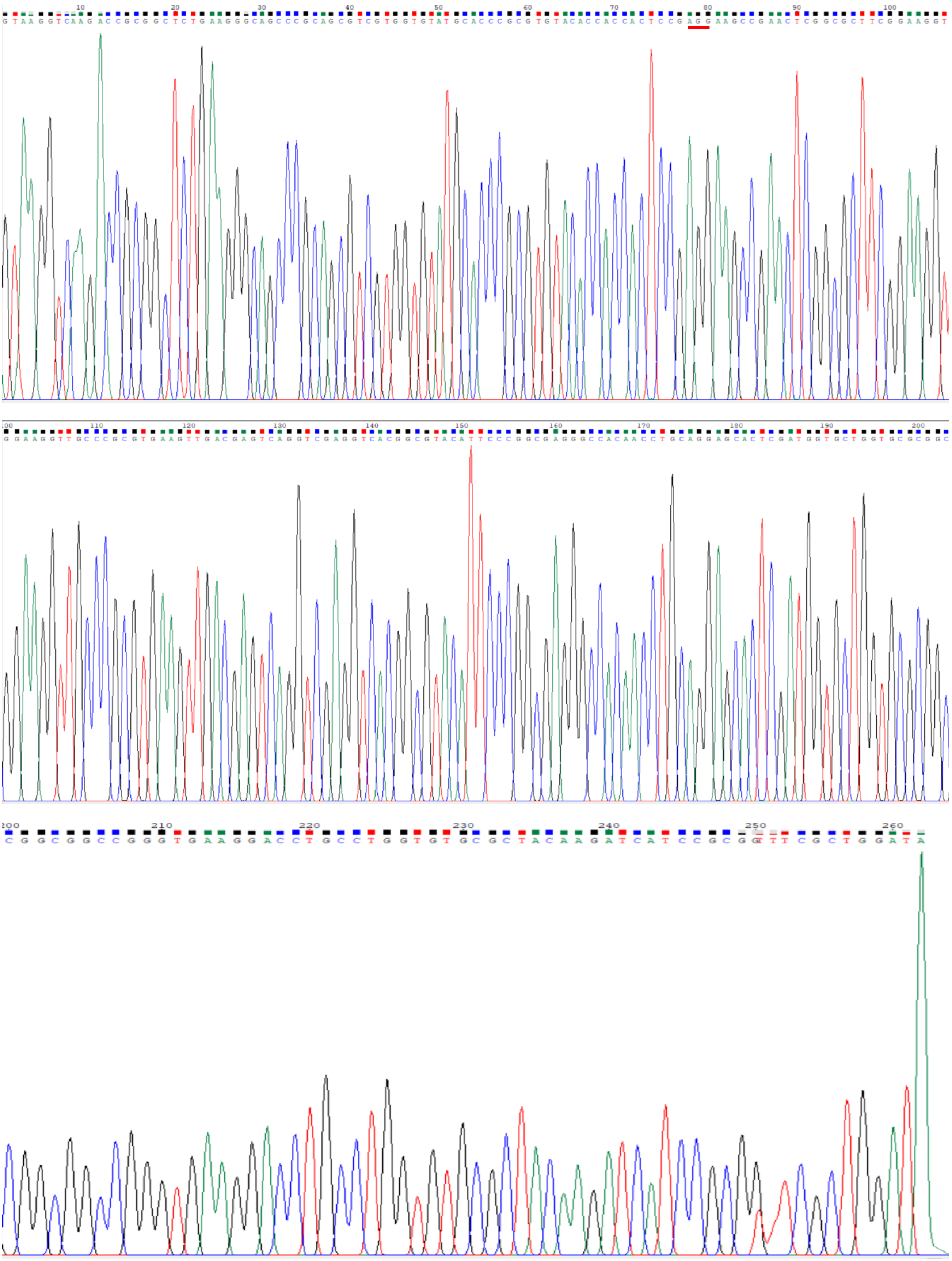

A13. Sequencing chromatograms of the *rpsL* gene, covering nucleotide positions 10–260, show an **AAG→AGG** substitution between nucleotide positions 70–80.

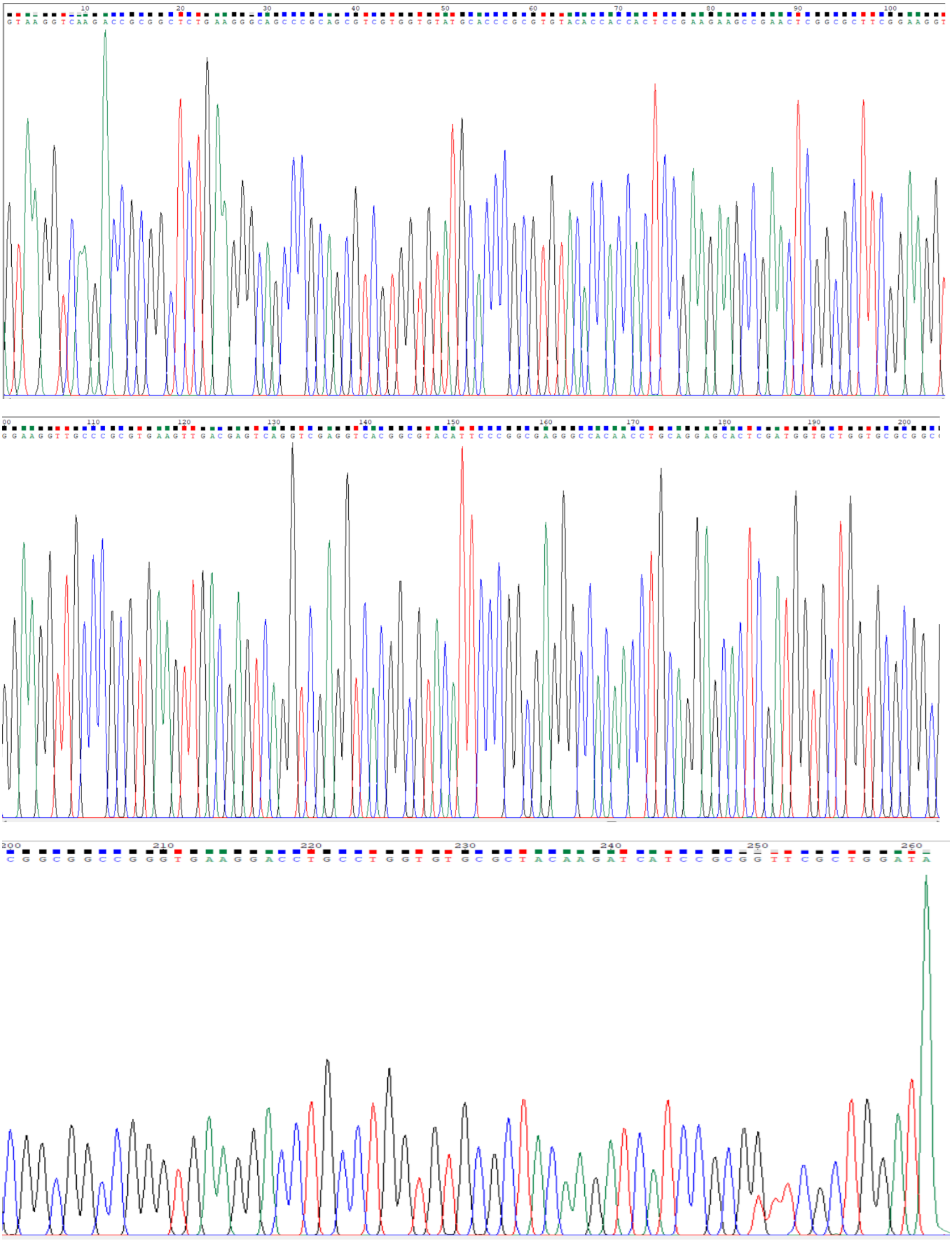

A14. Sequencing chromatograms of the *rpsL* gene, covering nucleotide positions 10–260, show a region with no detected mutations.

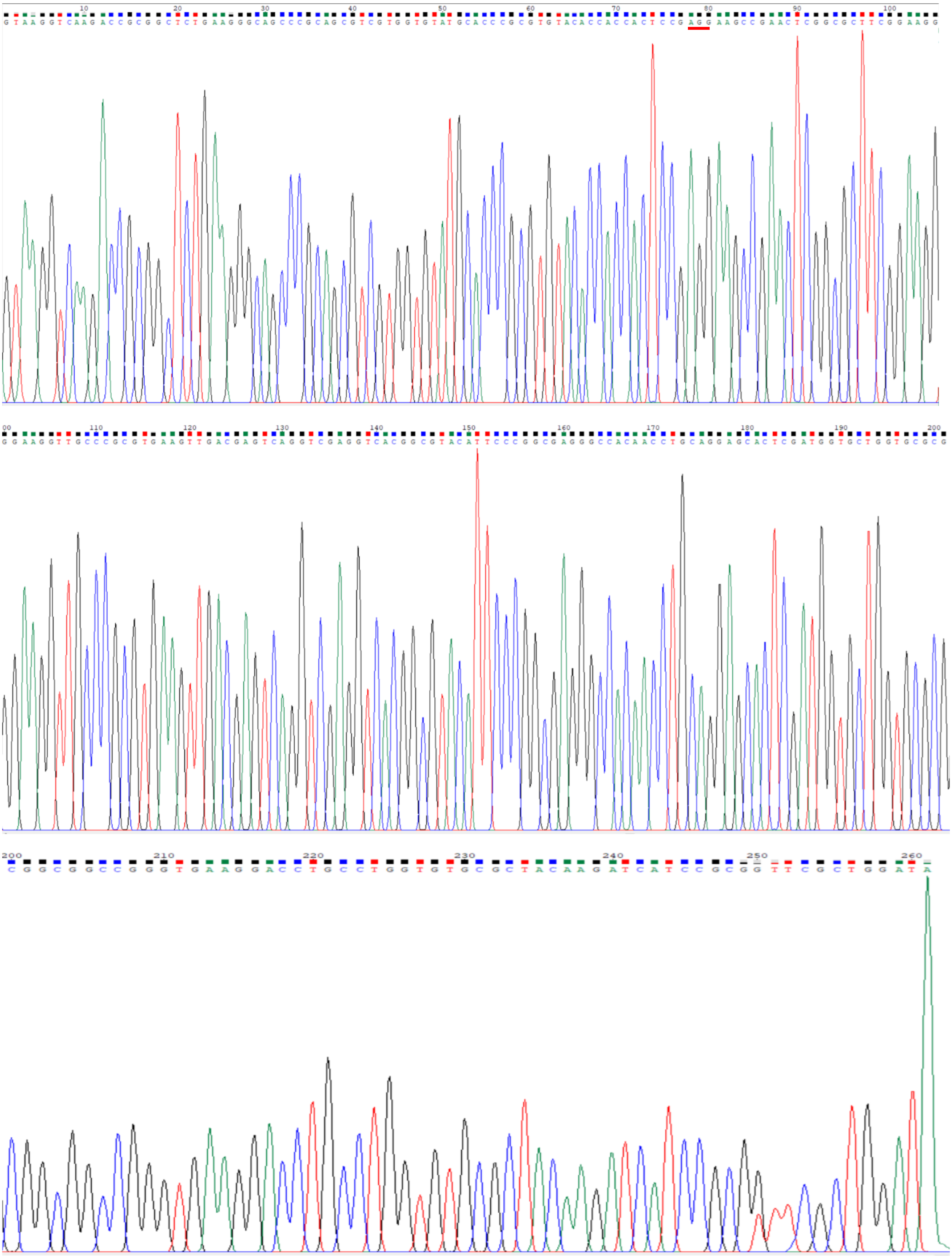

A15. Sequencing chromatograms of the *rpsL* gene, covering nucleotide positions 10–260, show an **AAG→AGG** substitution between nucleotide positions 70–80.

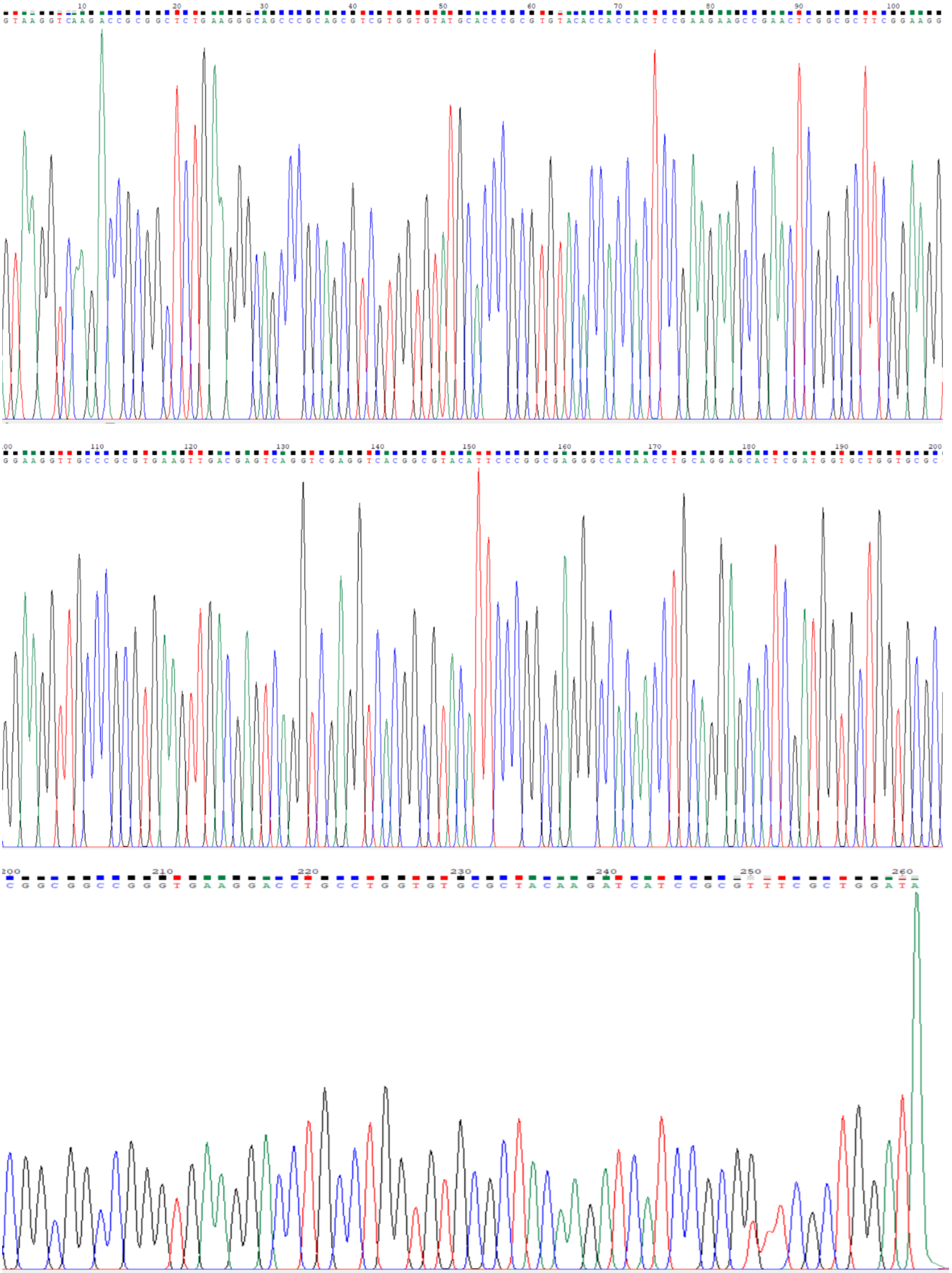

A16. Sequencing chromatograms of the *rpsL* gene, covering nucleotide positions 10–260, show a region with no detected mutations.

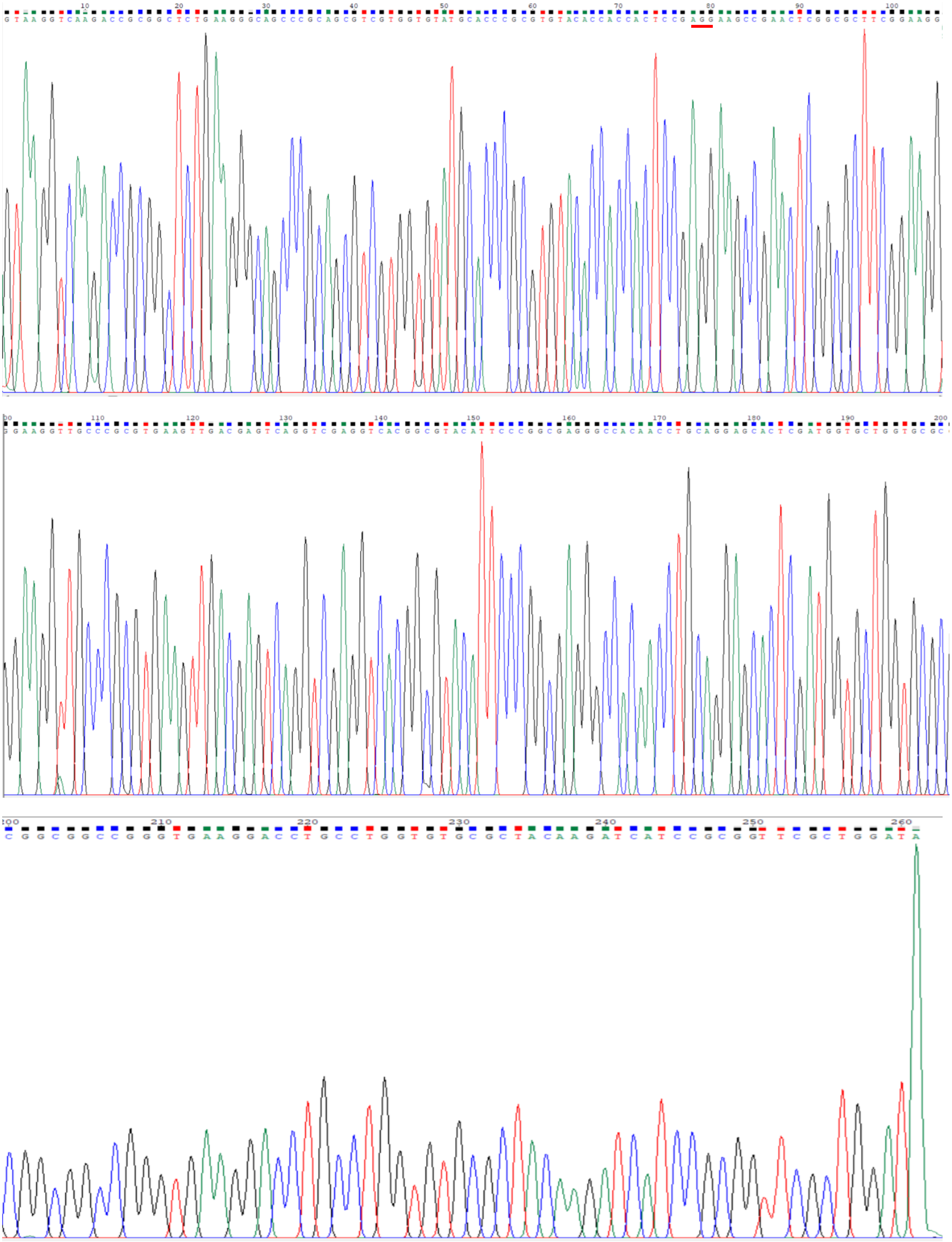

A17. Sequencing chromatograms of the *rpsL* gene, covering nucleotide positions 10–260, show an **AAG→AGG** substitution between nucleotide positions 70–80.

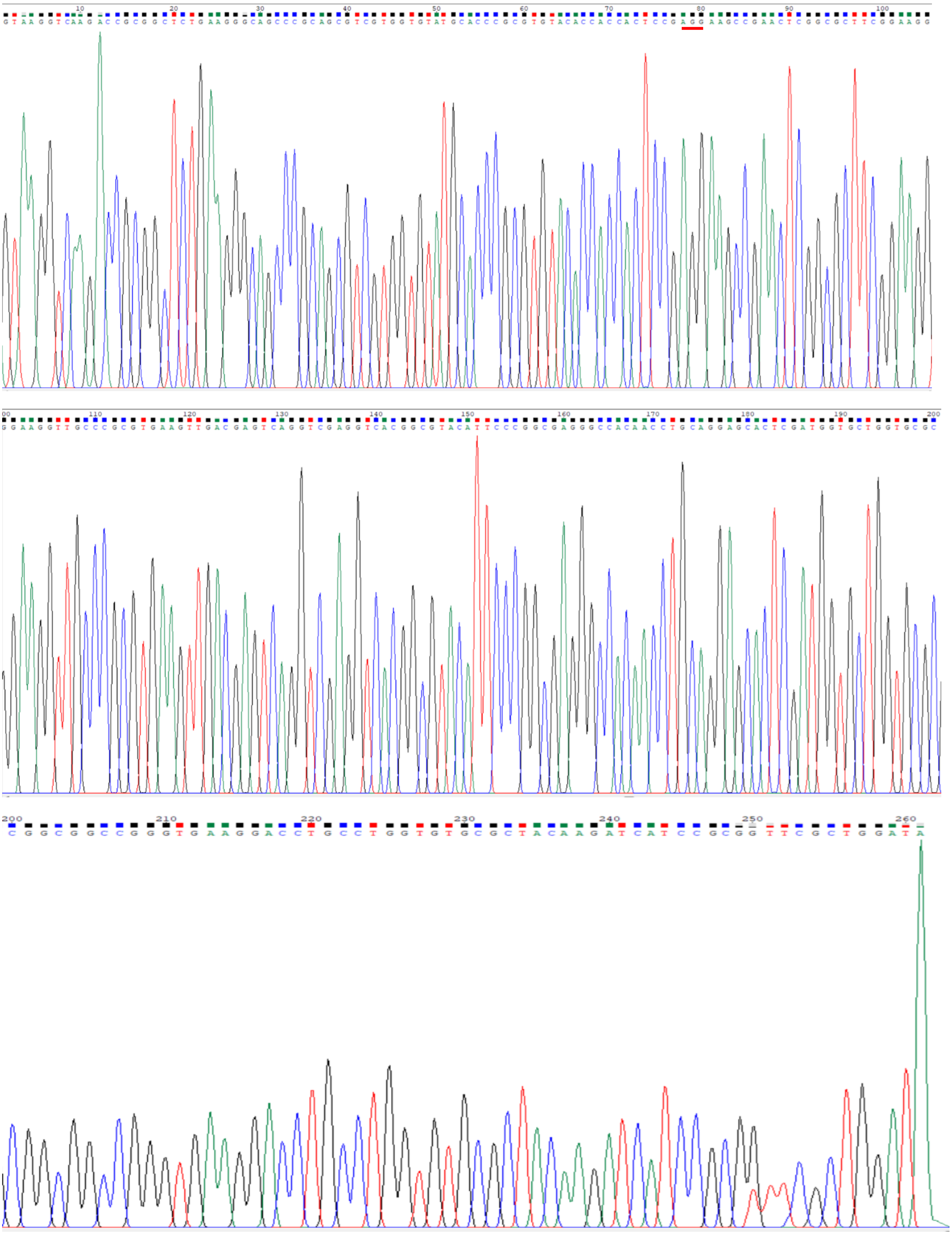

A18. Sequencing chromatograms of the *rpsL* gene, covering nucleotide positions 10–260, show an **AAG→AGG** substitution between nucleotide positions 70–80.

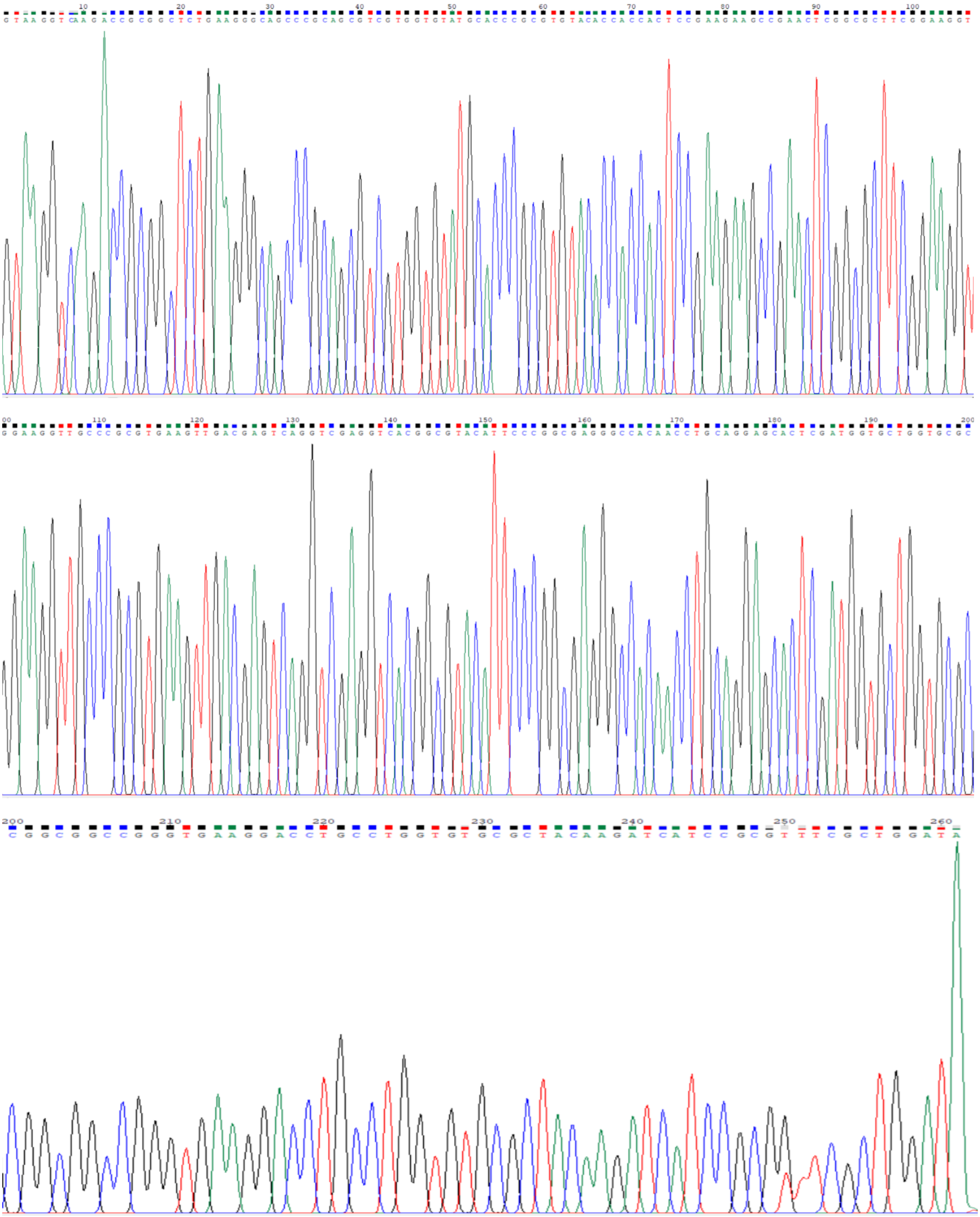

A19. Sequencing chromatograms of the *rpsL* gene, covering nucleotide positions 10–260, show a region with no detected mutations.

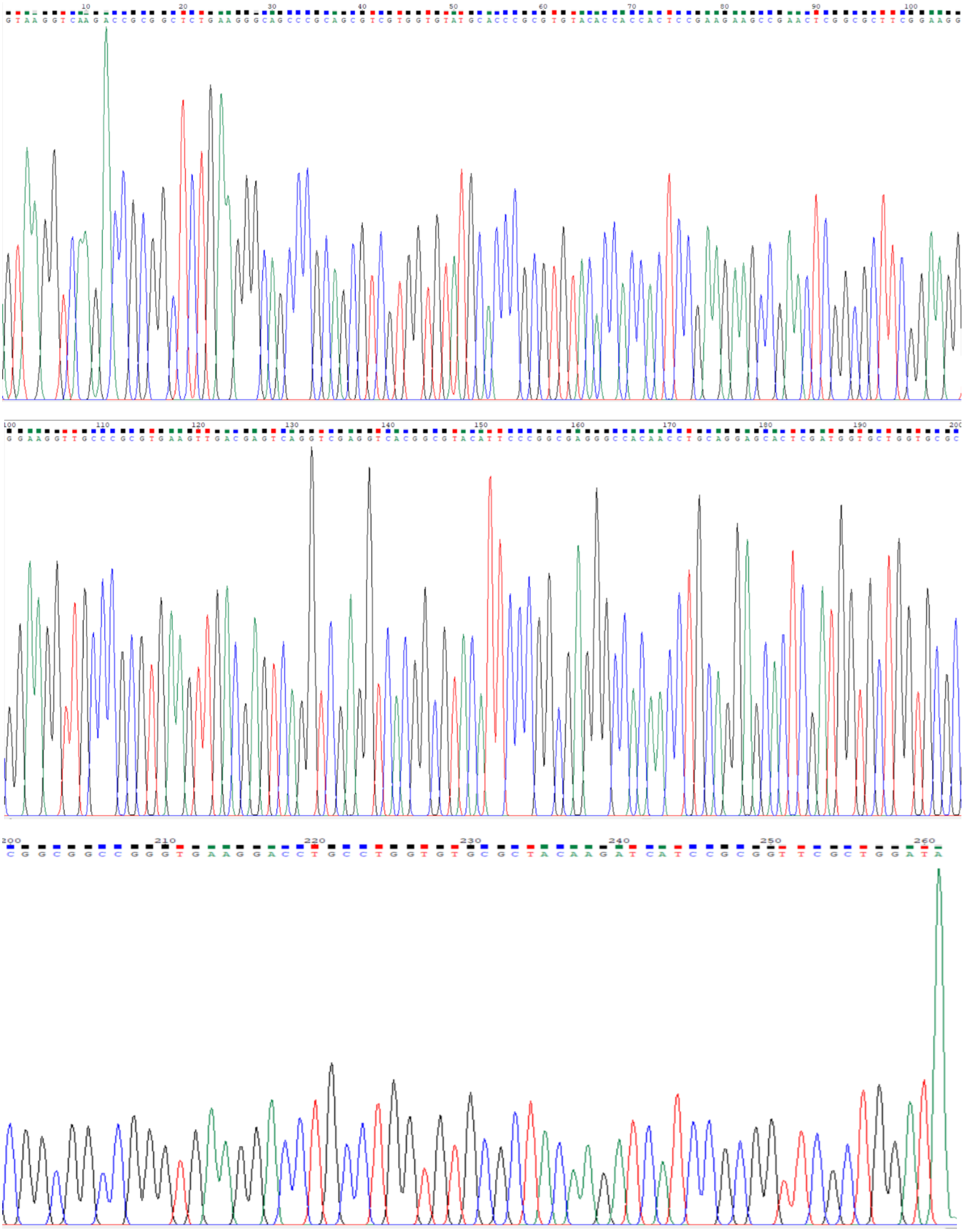

A20. Sequencing chromatograms of the *rpsL* gene, covering nucleotide positions 10–260, show a region with no detected mutations.

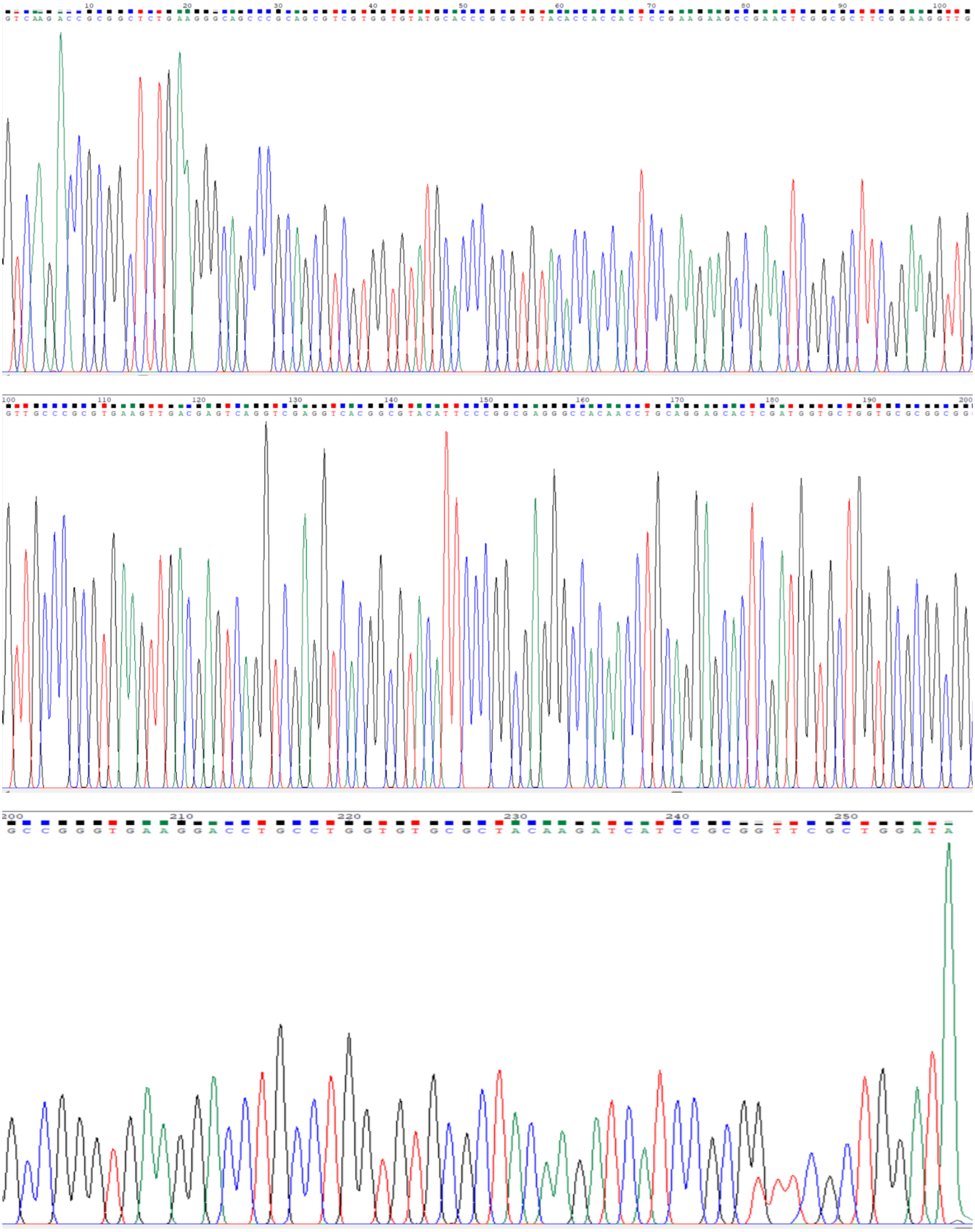

A21. Sequencing chromatograms of the *rpsL* gene, covering nucleotide positions 10–260, show a region with no detected mutations.

Chromatogram images of the *katG* gene region from 19 isolates (B1-B19).

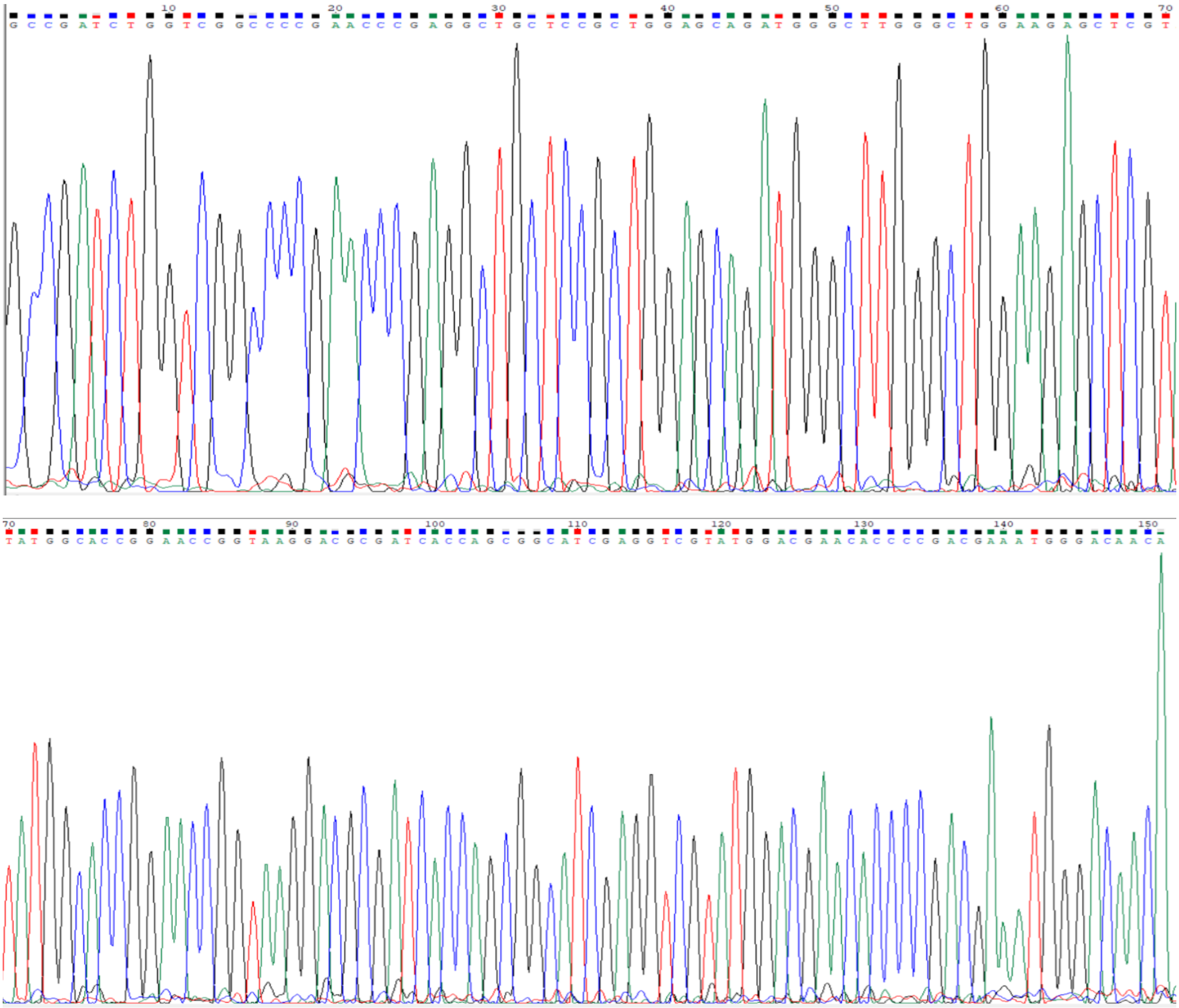

B1. Sequencing chromatograms of the *katG* gene, covering nucleotide positions 10–150, show a region with no detected mutations.

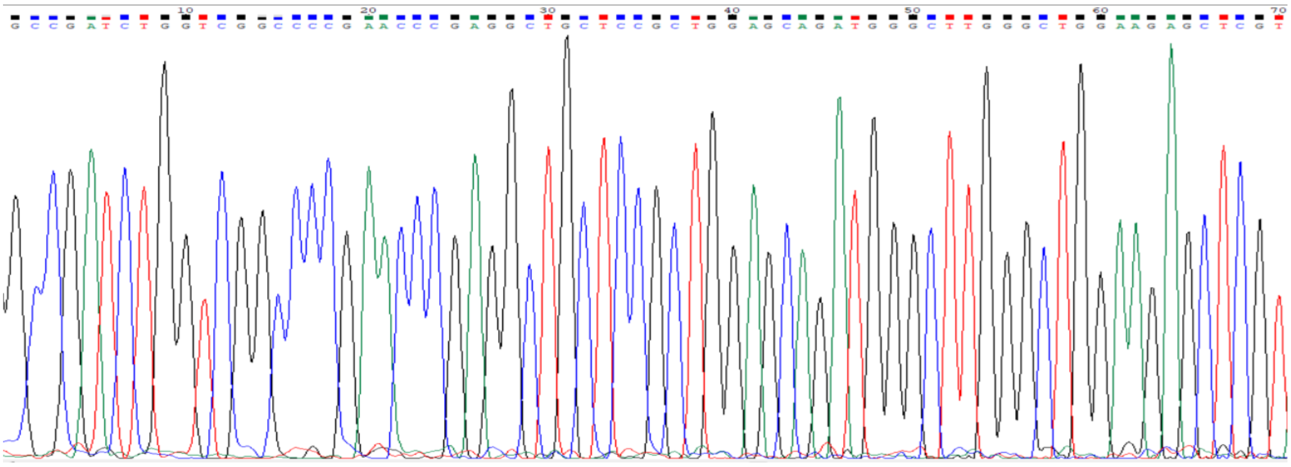

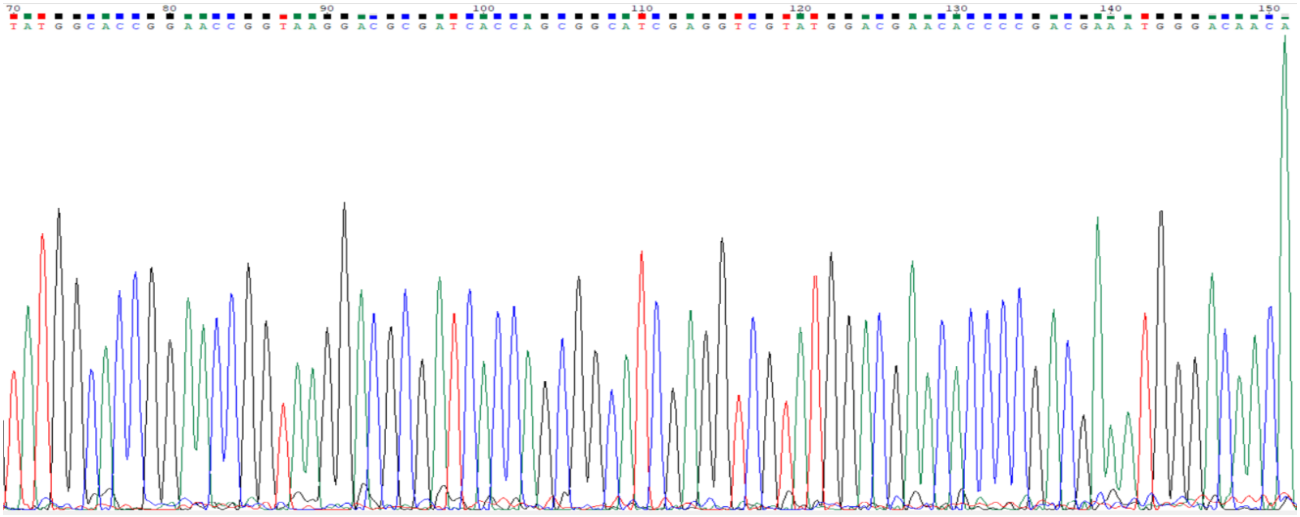

B2. Sequencing chromatograms of the *katG* gene, covering nucleotide positions 10–150, show a region with no detected mutations.

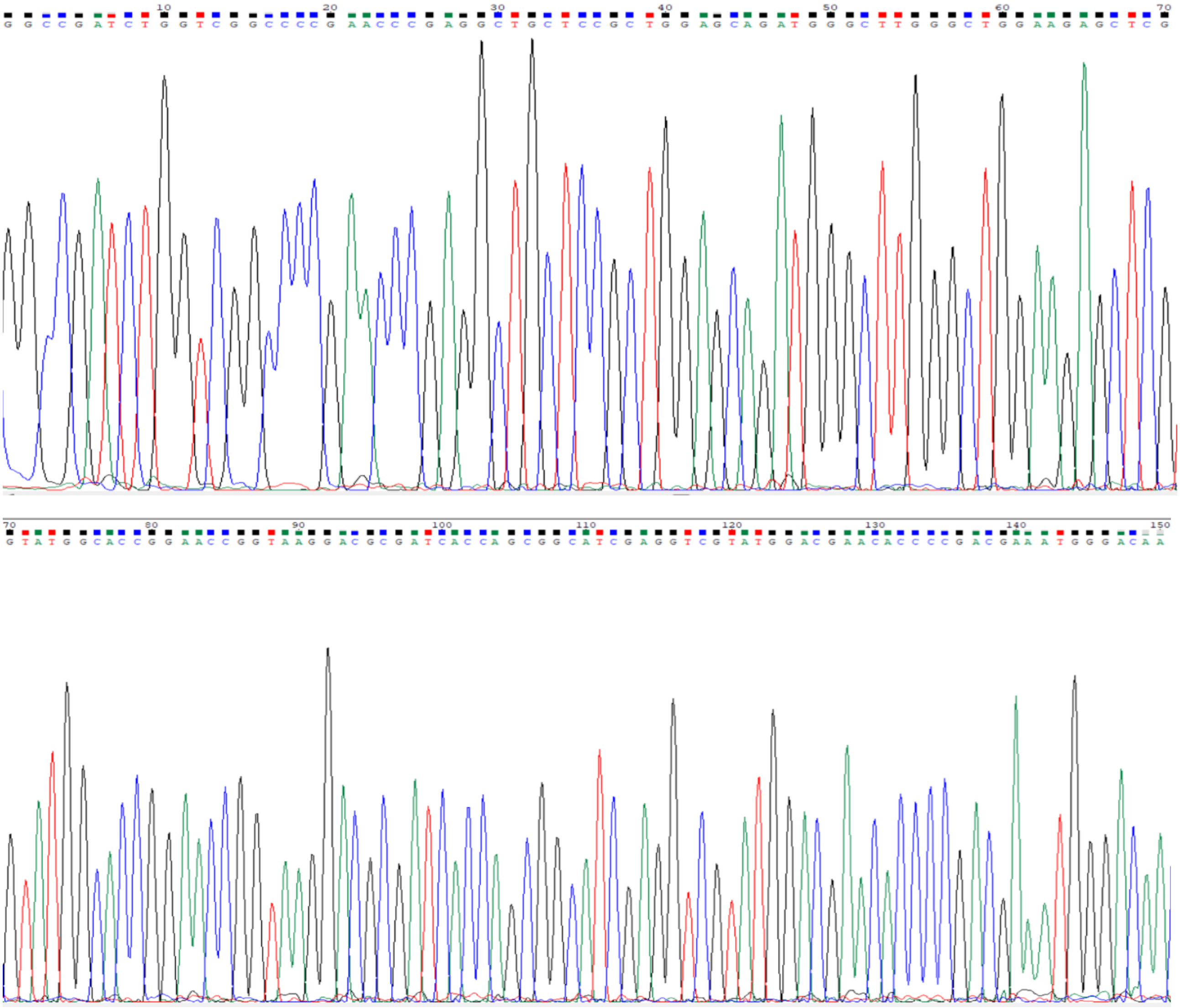

B3. Sequencing chromatograms of the *katG* gene, covering nucleotide positions 10–150, show a region with no detected mutations.

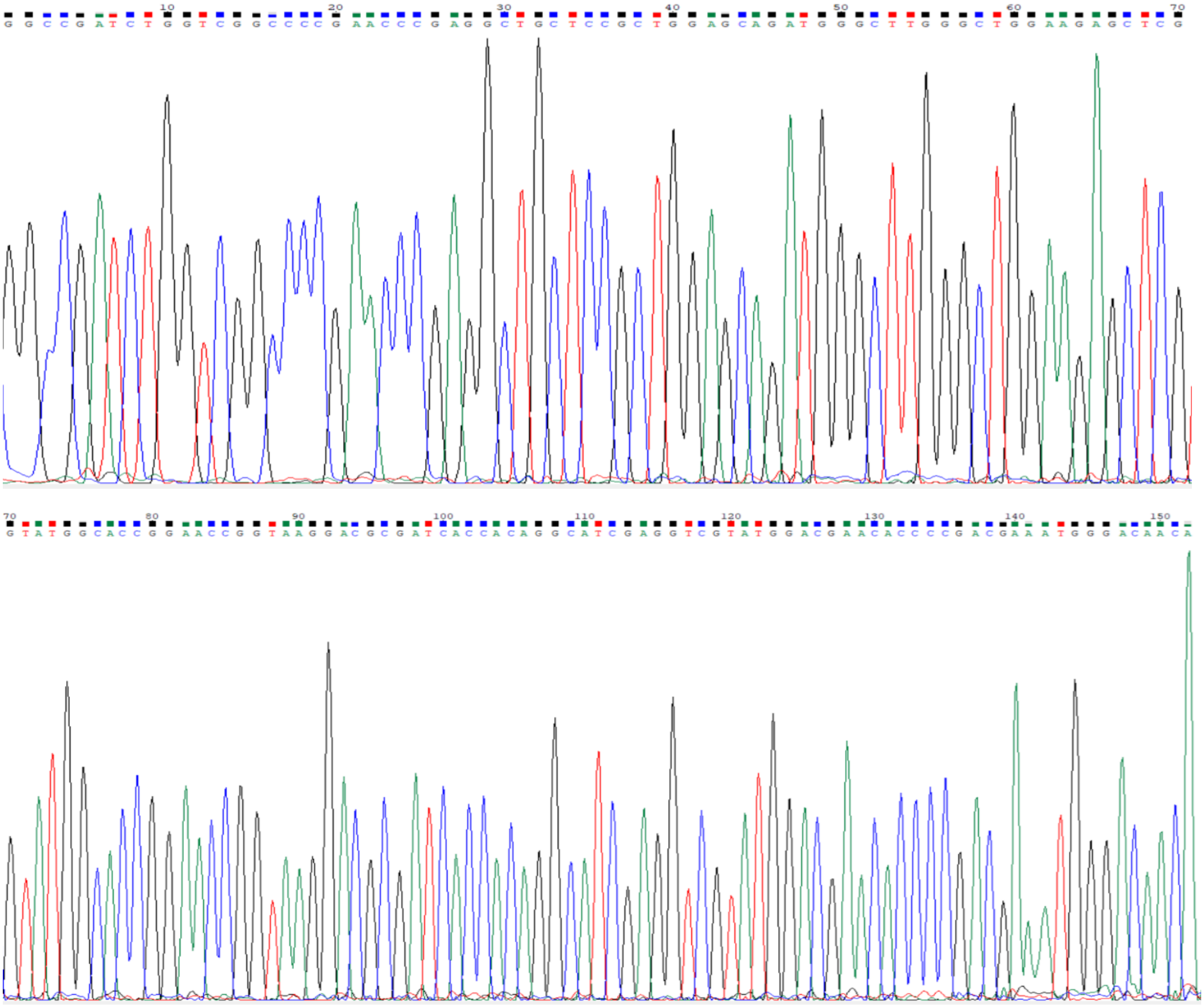

B4. Sequencing chromatograms of the *katG* gene, covering nucleotide positions 10–150, show a region with no detected mutations.

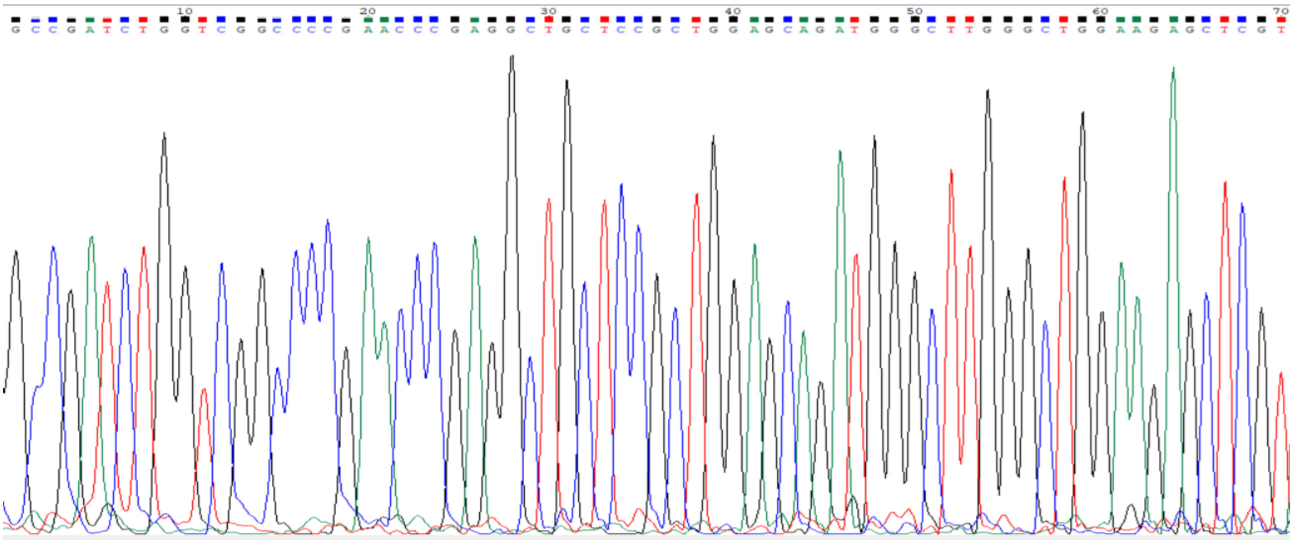

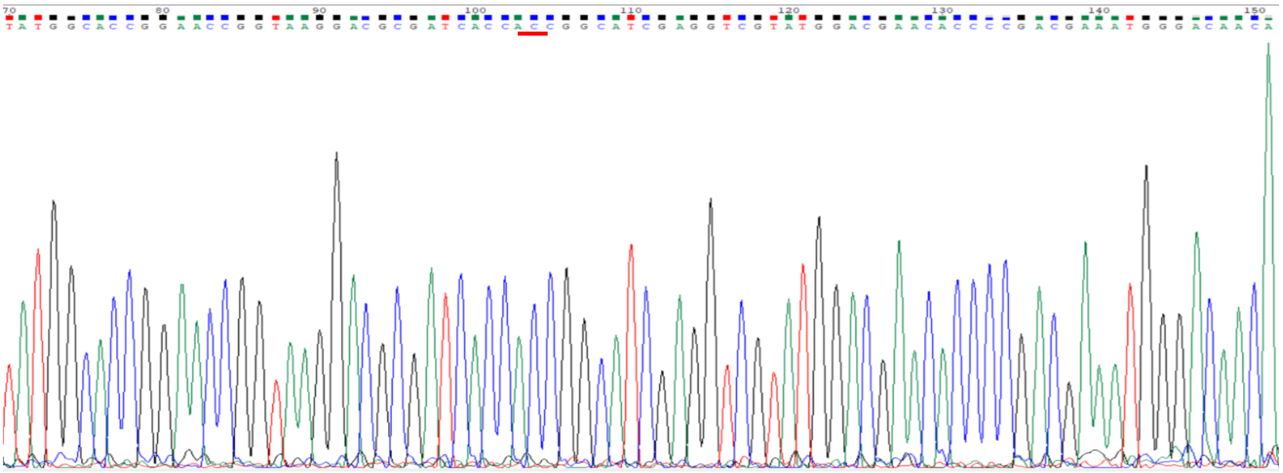

B5. Sequencing chromatograms of the *katG* gene, covering nucleotide positions 10–150, show an **AGC→ACC** substitution between nucleotide positions 100–110.

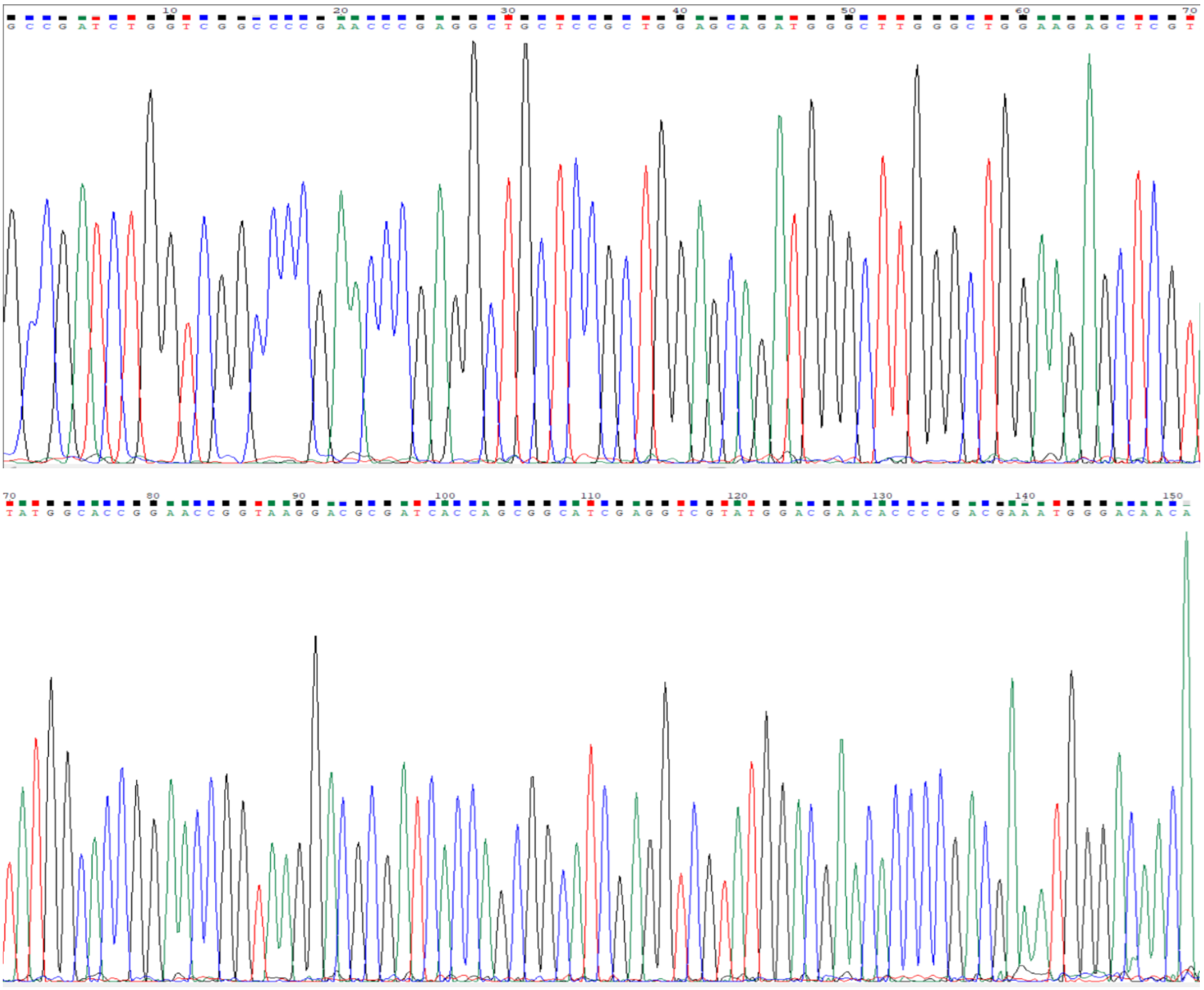

B6. Sequencing chromatograms of the *katG* gene, covering nucleotide positions 10–150, show a region with no detected mutations.

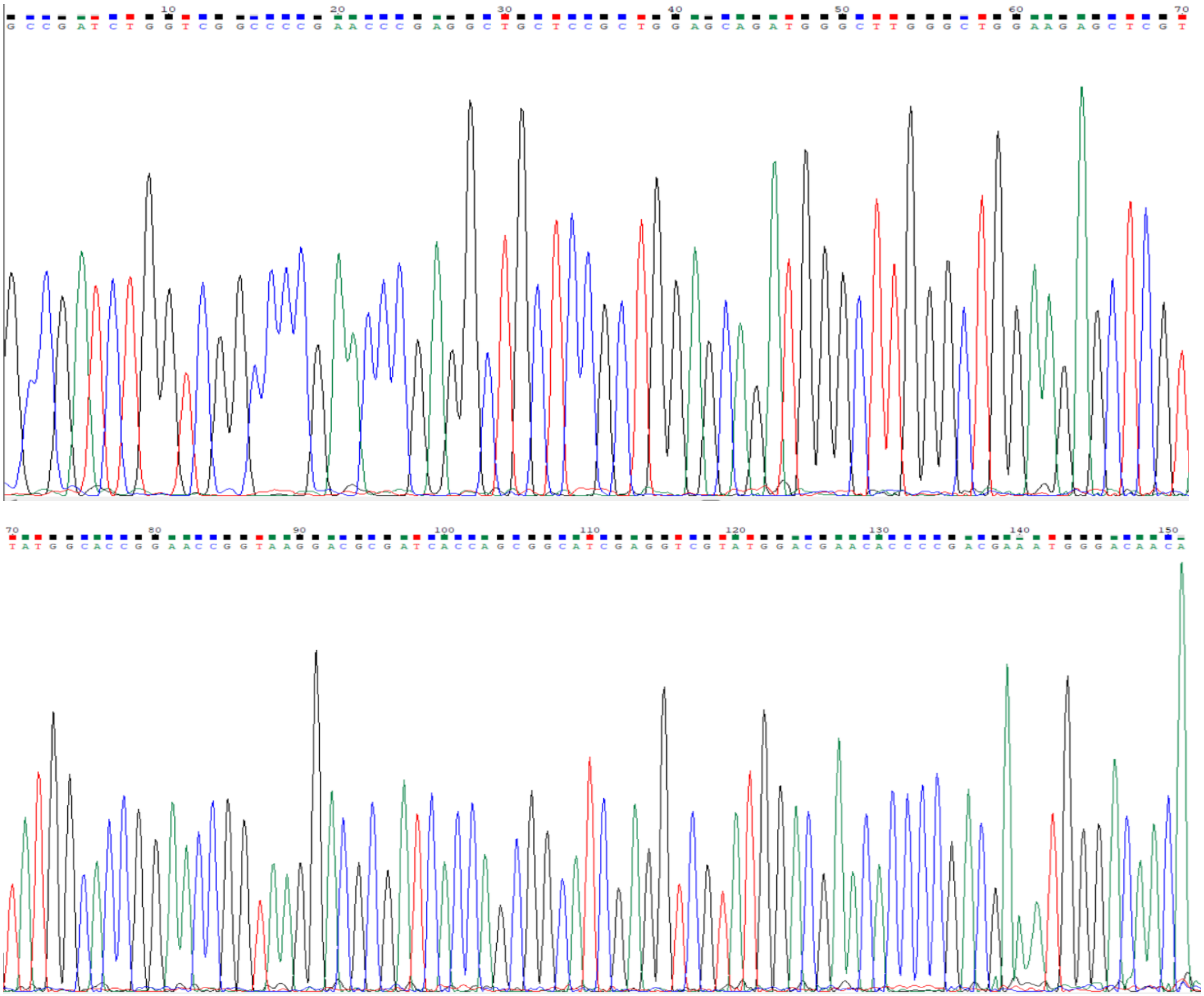

B7. Sequencing chromatograms of the *katG* gene, covering nucleotide positions 10–150, show a region with no detected mutations.

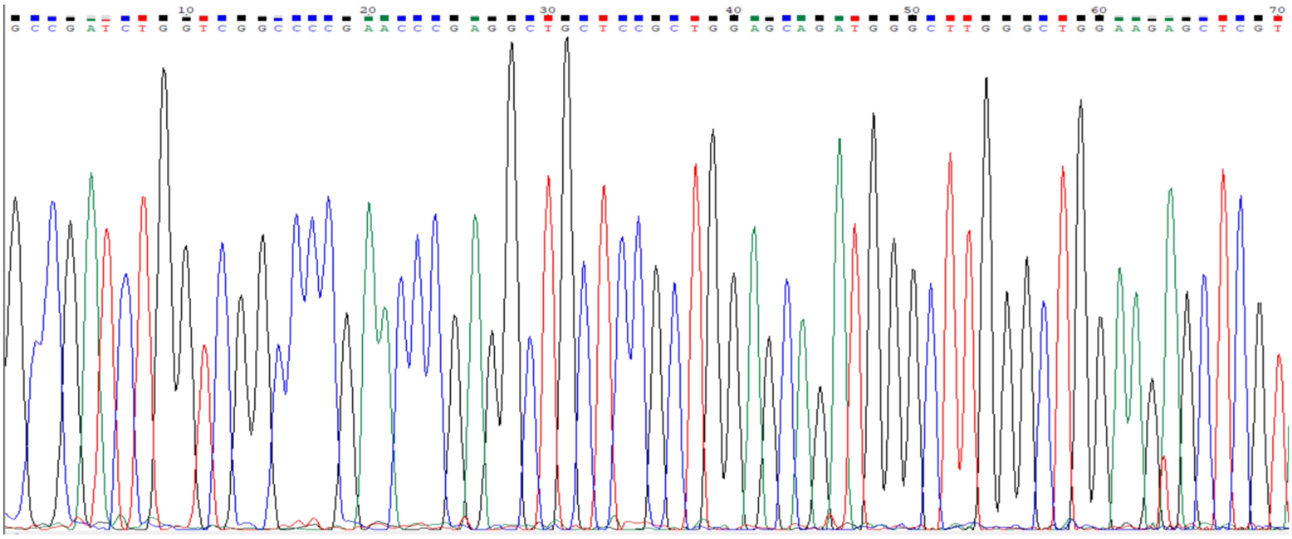

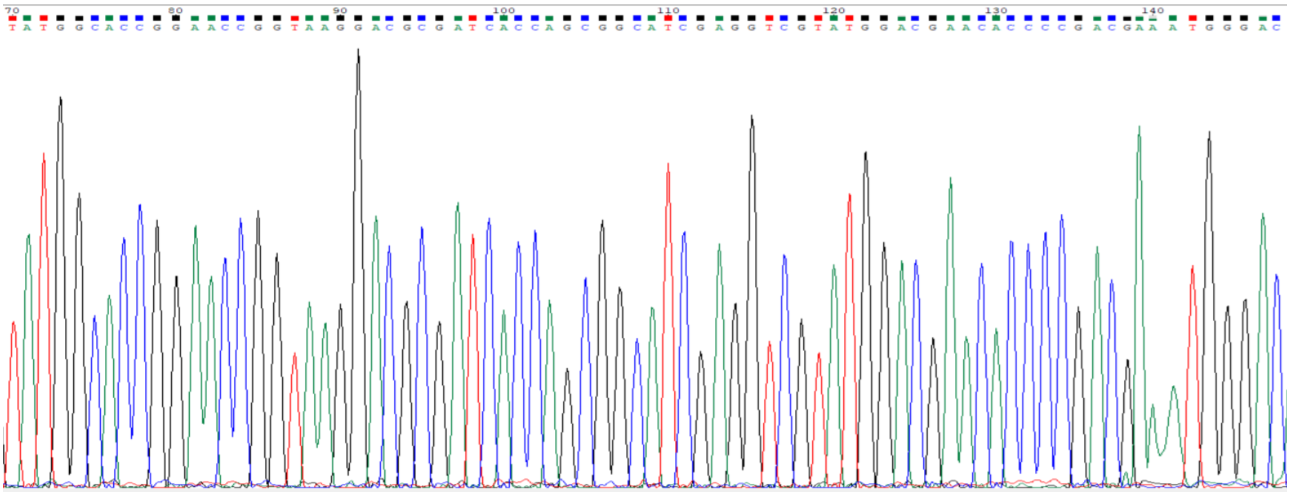

B8. Sequencing chromatograms of the *katG* gene, covering nucleotide positions 10–140, show a region with no detected mutations.

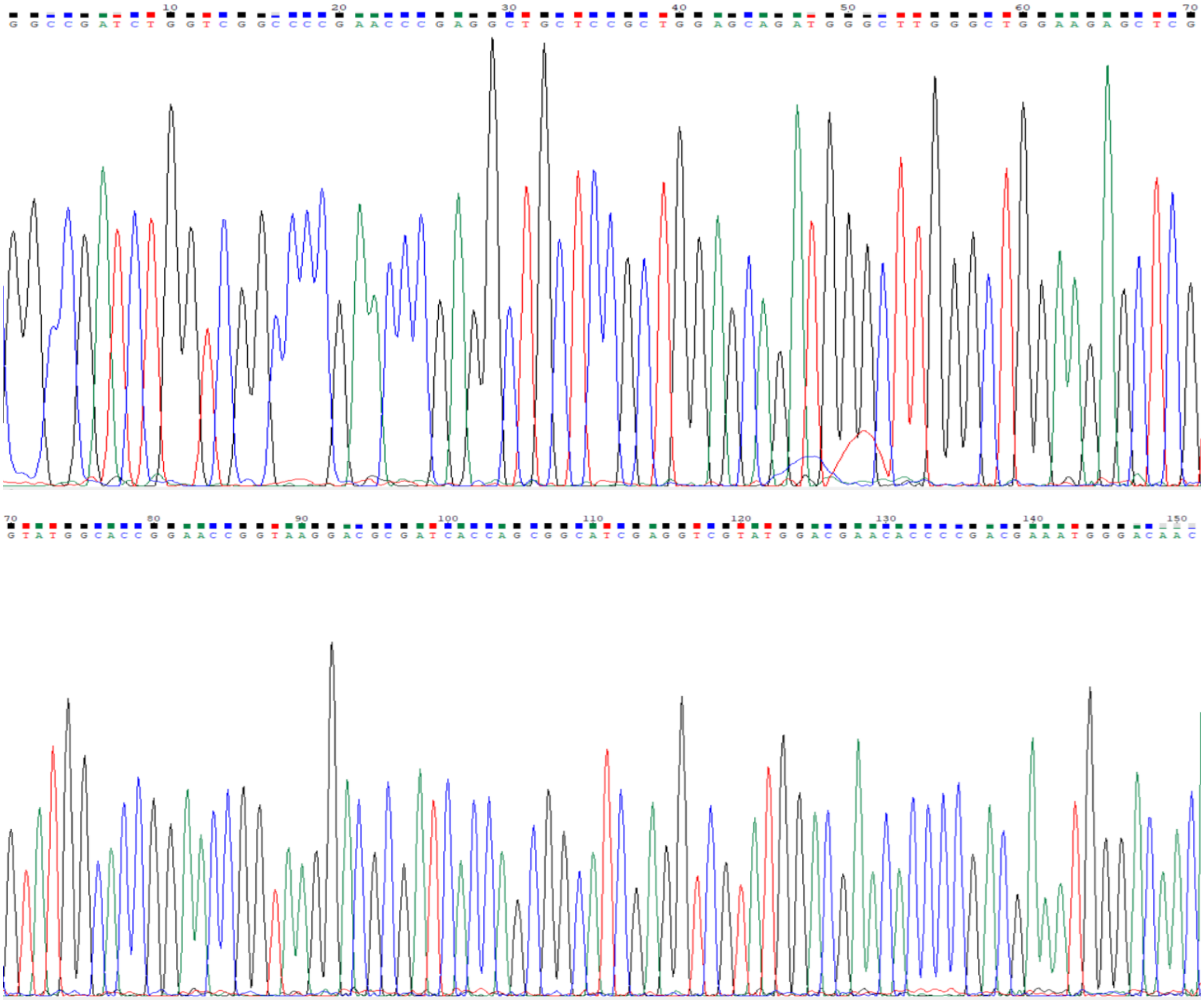

B9. Sequencing chromatograms of the *katG* gene, covering nucleotide positions 10–150, show a region with no detected mutations.

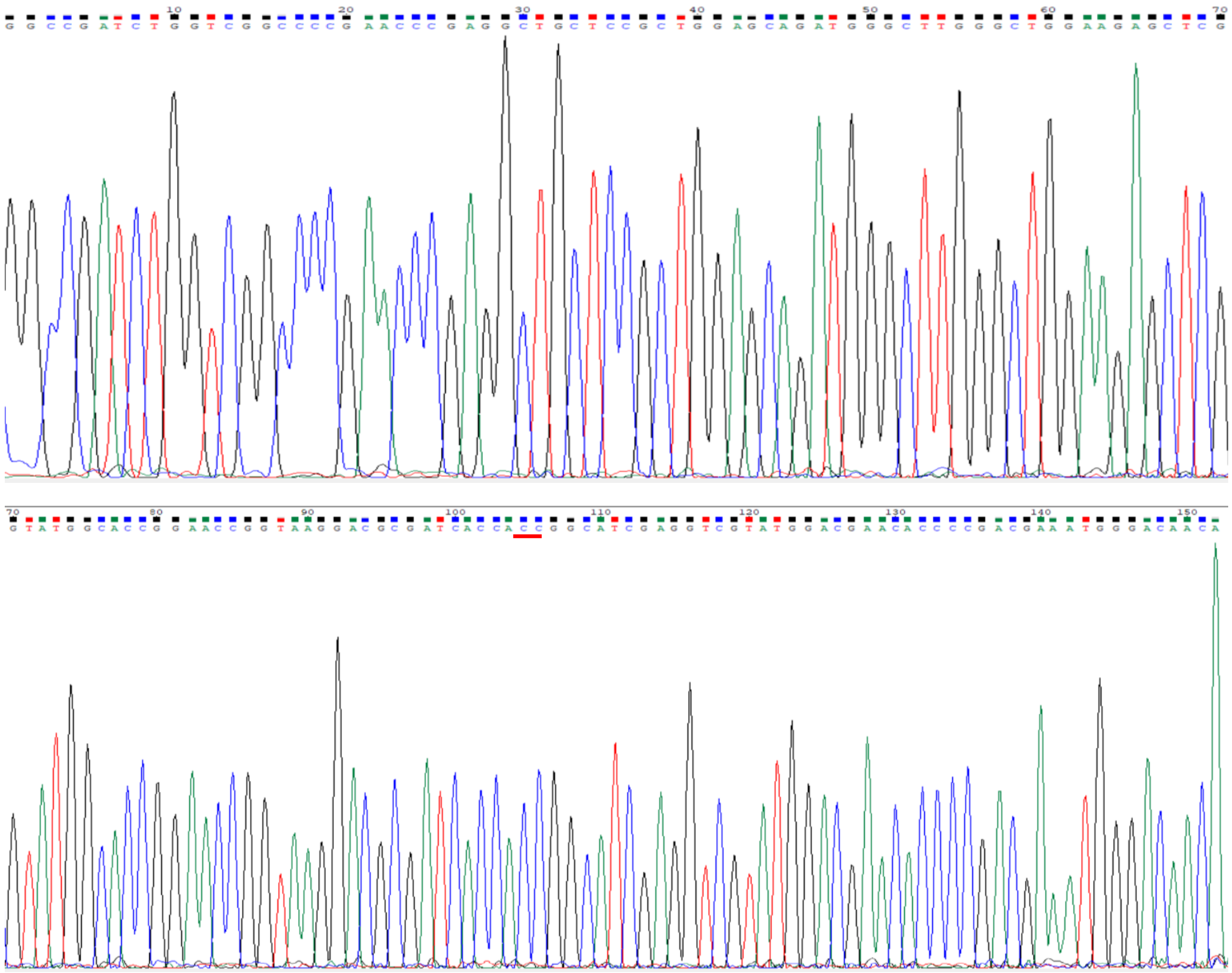

B10. Sequencing chromatograms of the *katG* gene, covering nucleotide positions 10–150, show an **AGC→ACC** substitution between nucleotide positions 100–110.

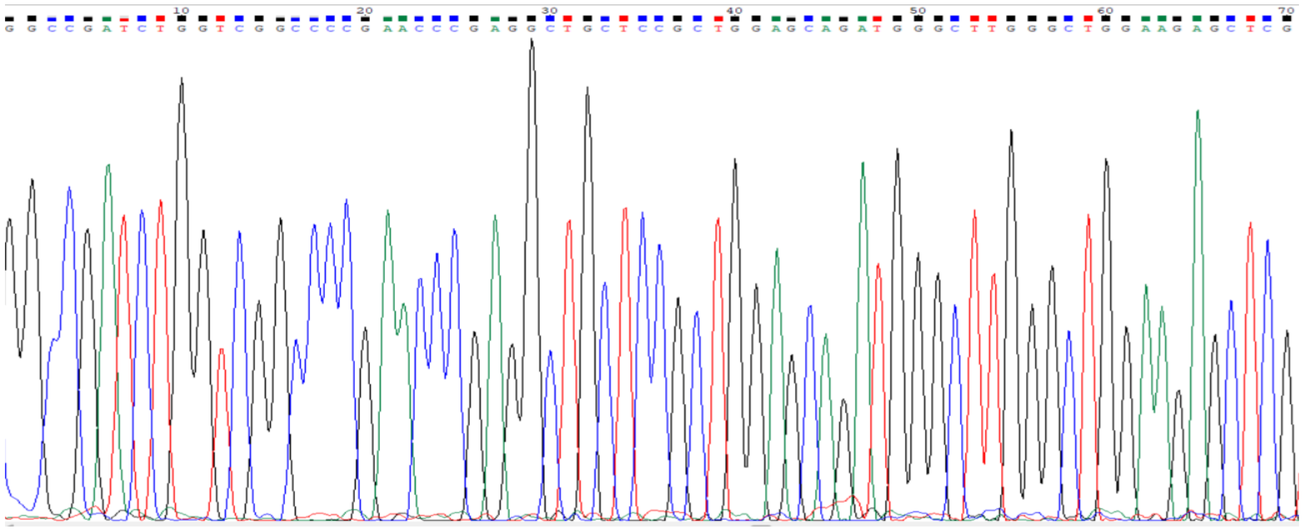

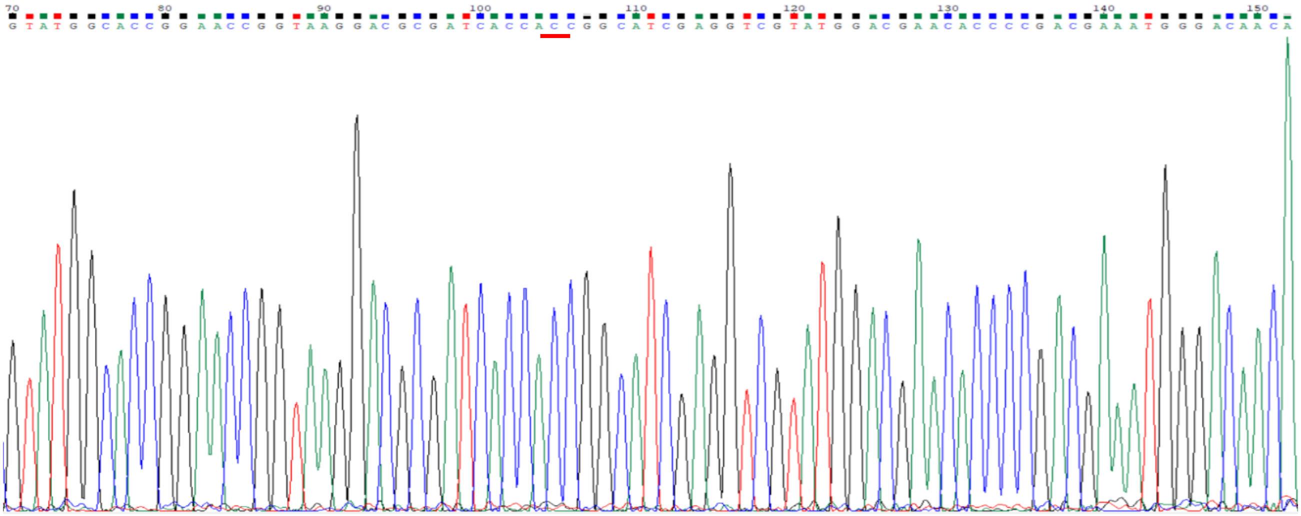

B11. Sequencing chromatograms of the *katG* gene, covering nucleotide positions 10–150, show an **AGC→ACC** substitution between nucleotide positions 100–110.

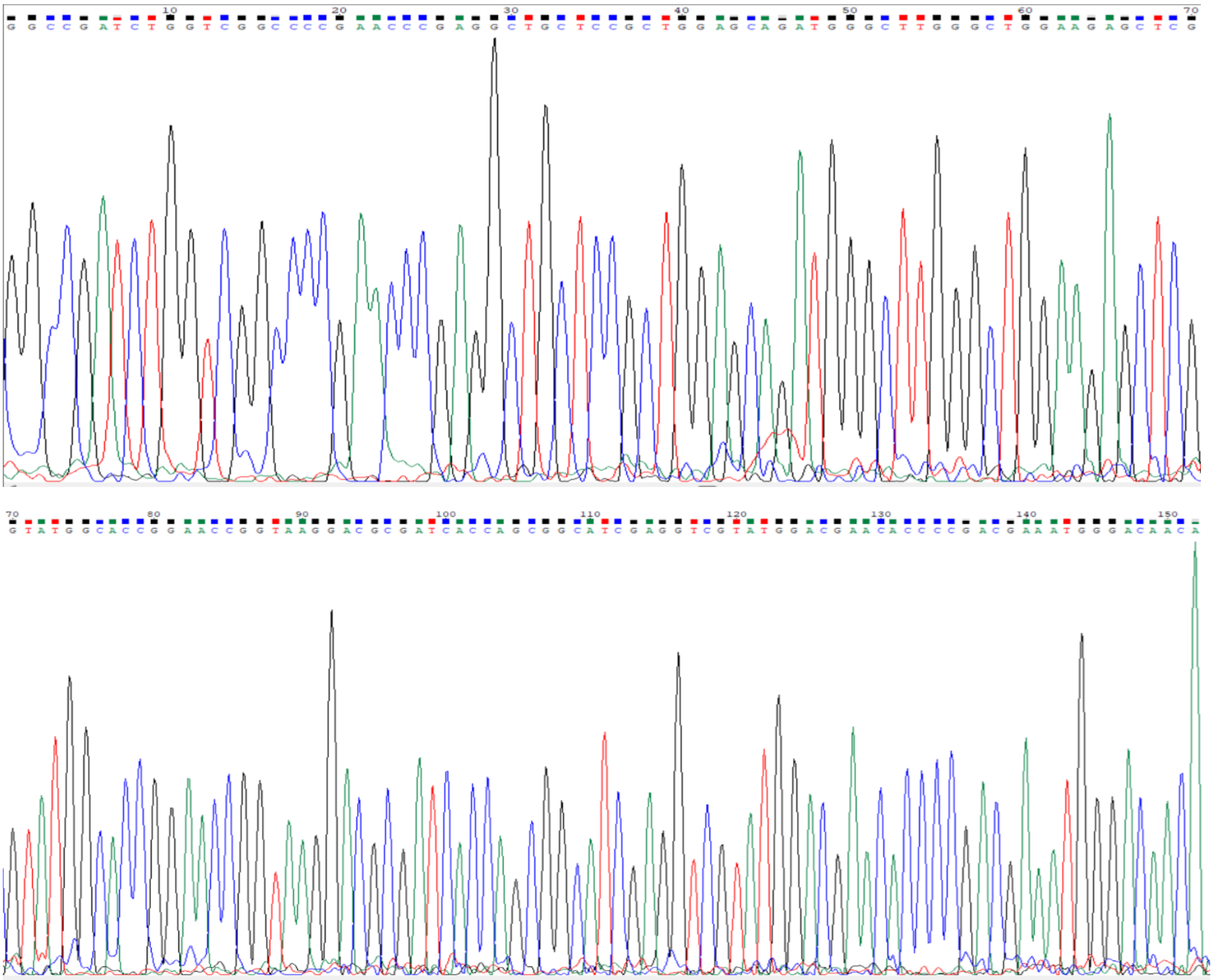

B12. Sequencing chromatograms of the *katG* gene, covering nucleotide positions 10–150, show a region with no detected mutations.

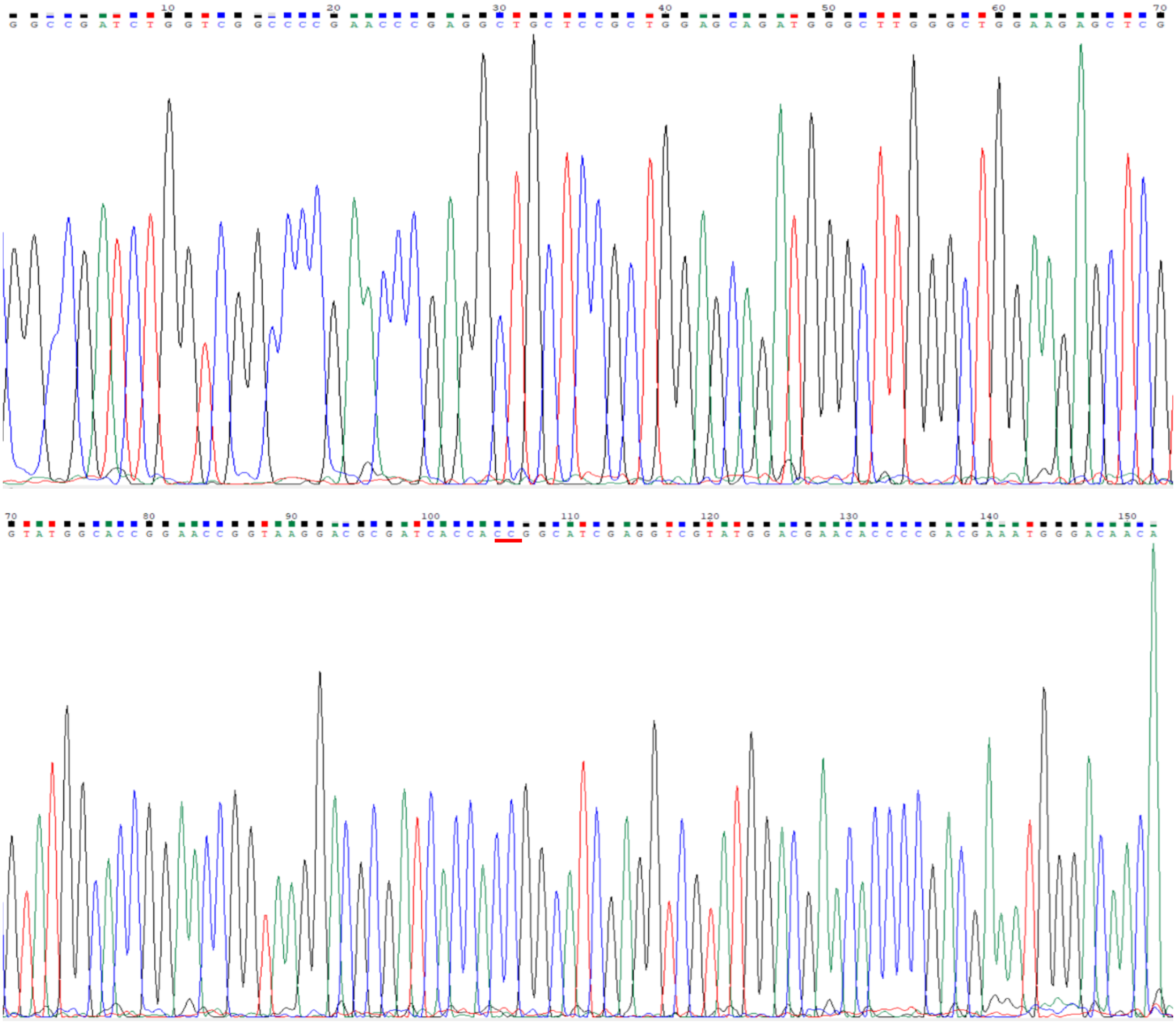

B13. Sequencing chromatograms of the *katG* gene, covering nucleotide positions 10–150, show an **AGC→ACC** substitution between nucleotide positions 100–110.

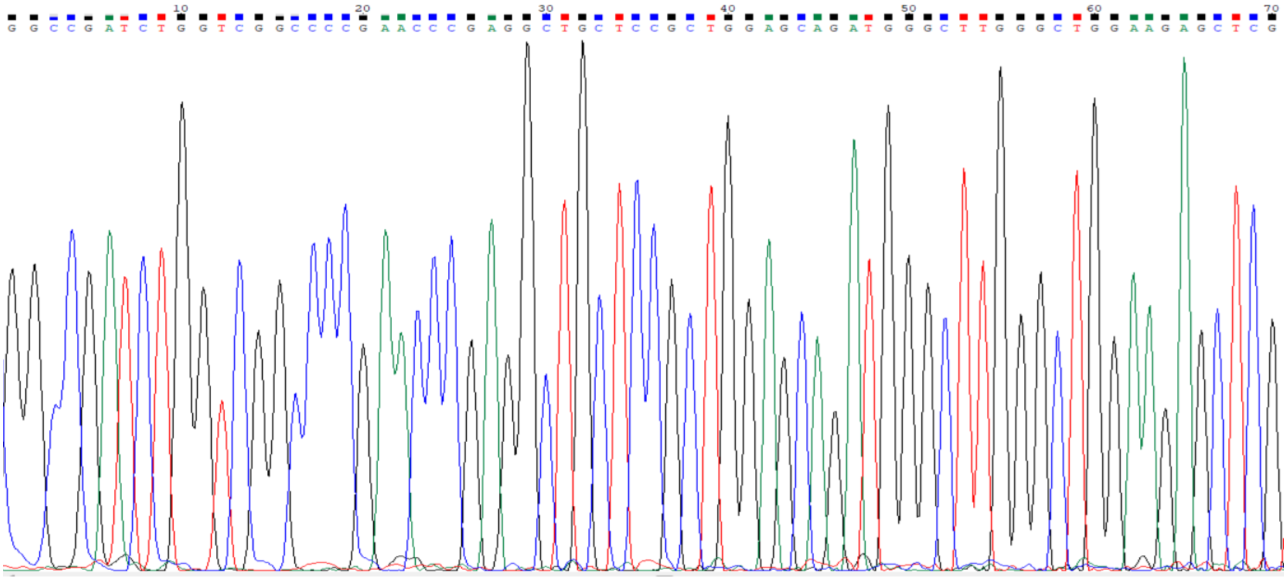

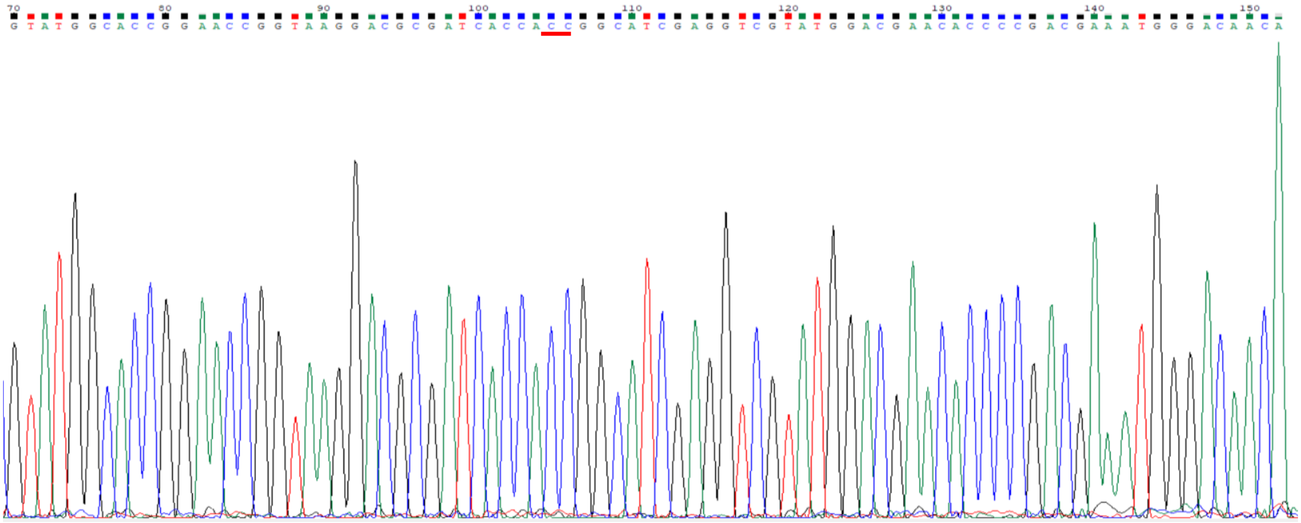

B14. Sequencing chromatograms of the *katG* gene, covering nucleotide positions 10–150, show an **AGC→ACC** substitution between nucleotide positions 100–110.

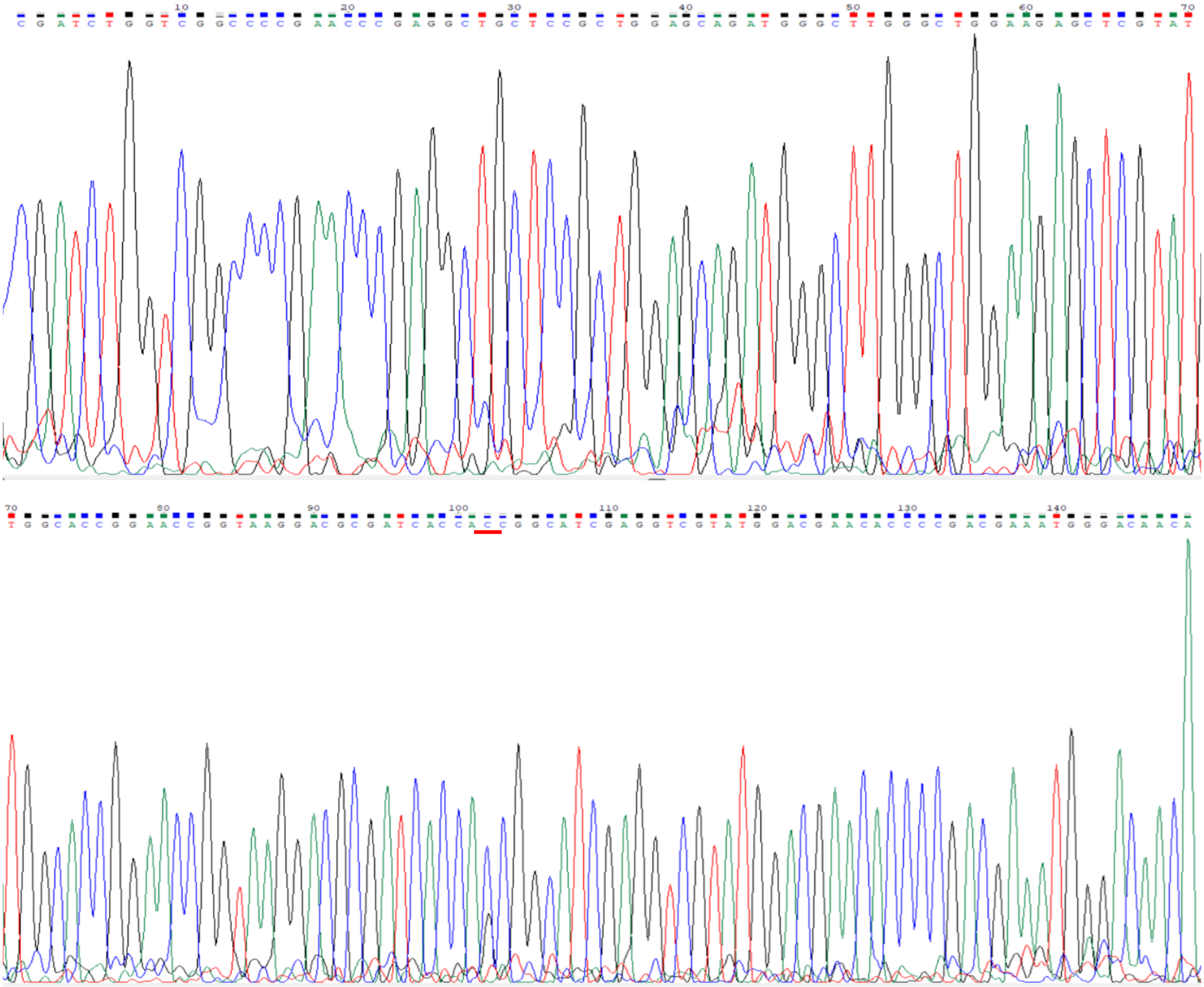

B15. Sequencing chromatograms of the *katG* gene, covering nucleotide positions 10–140, show an **AGC→ACC** substitution between nucleotide positions 100–110.

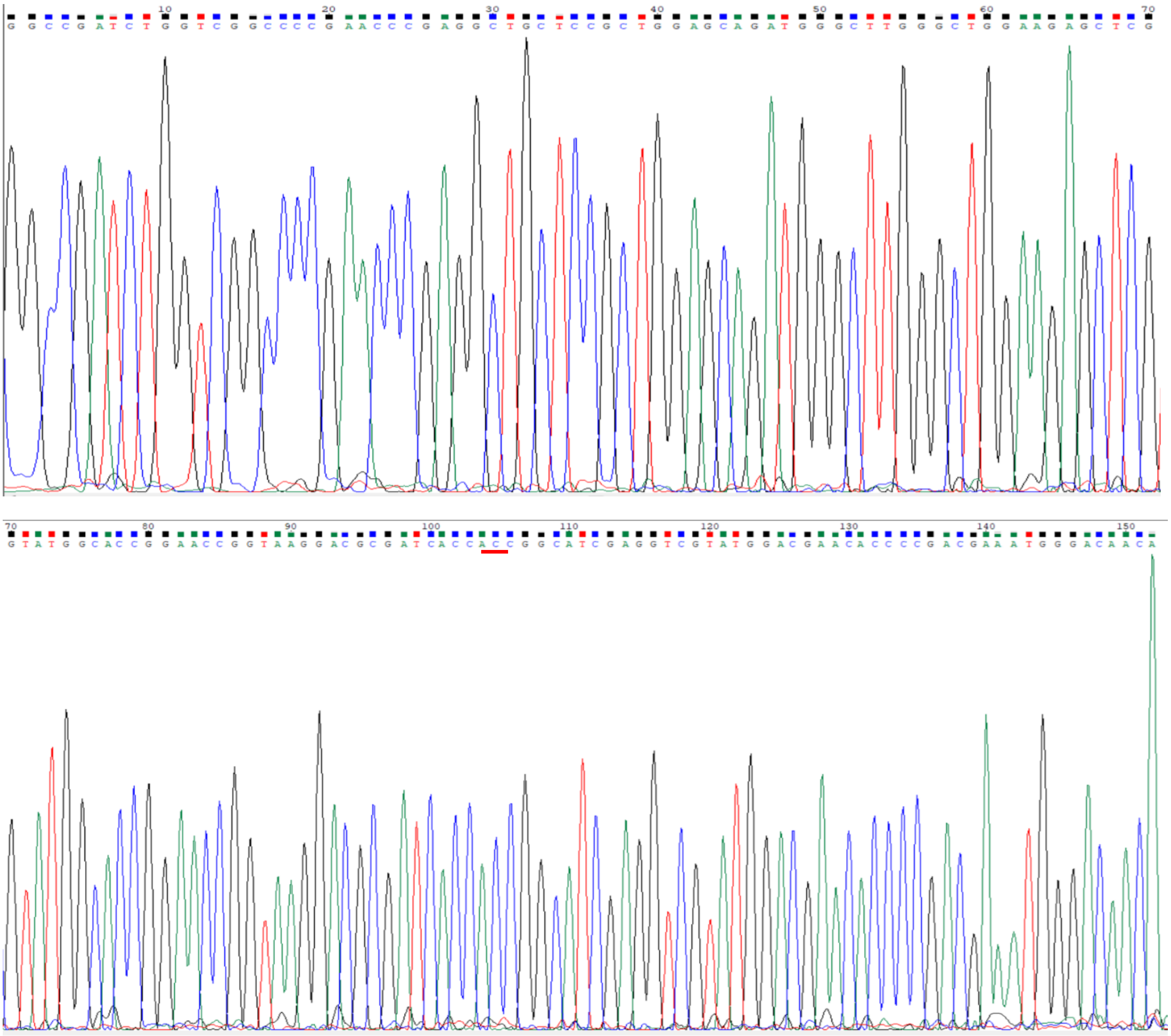

B16. Sequencing chromatograms of the *katG* gene, covering nucleotide positions 10–150, show an **AGC→ACC** substitution between nucleotide positions 100–110.

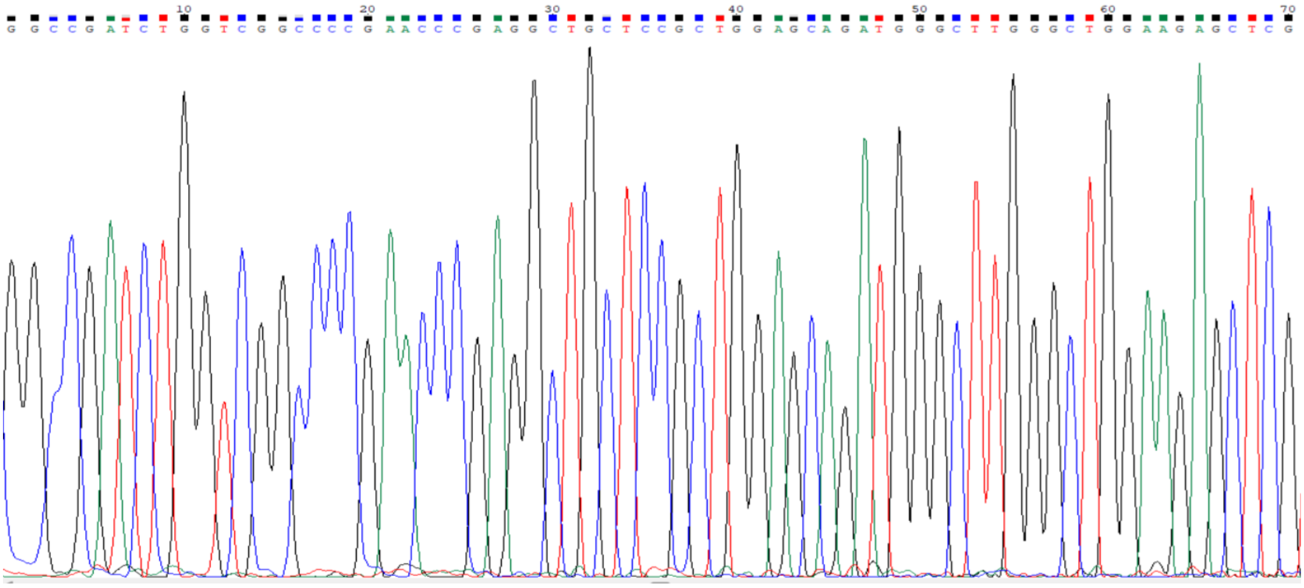

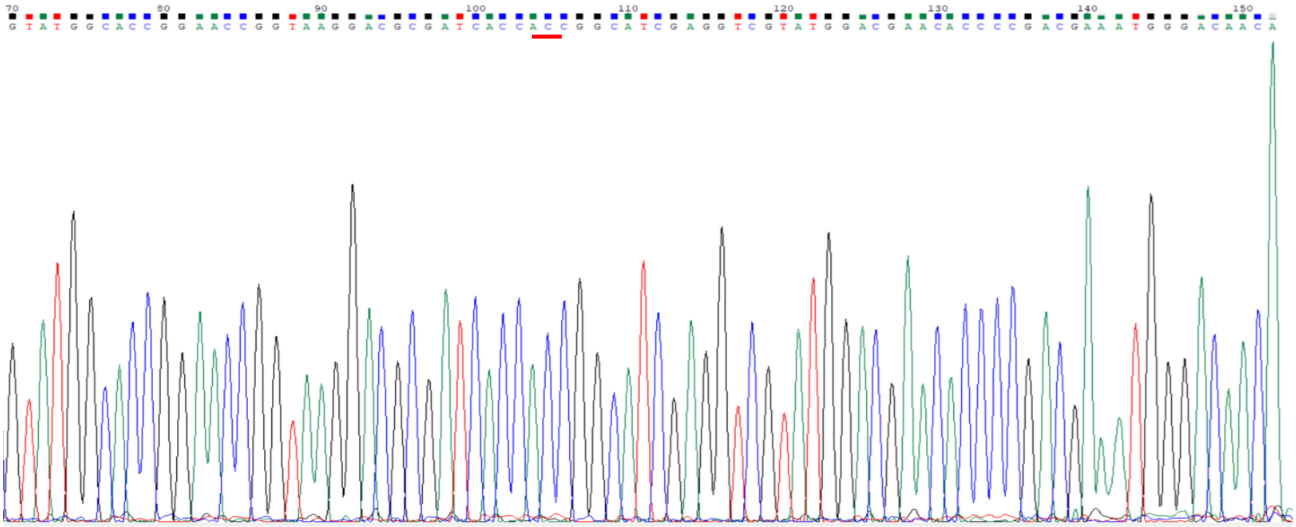

B17. Sequencing chromatograms of the *katG* gene, covering nucleotide positions 10–150, show an **AGC→ACC** substitution between nucleotide positions 100–110.

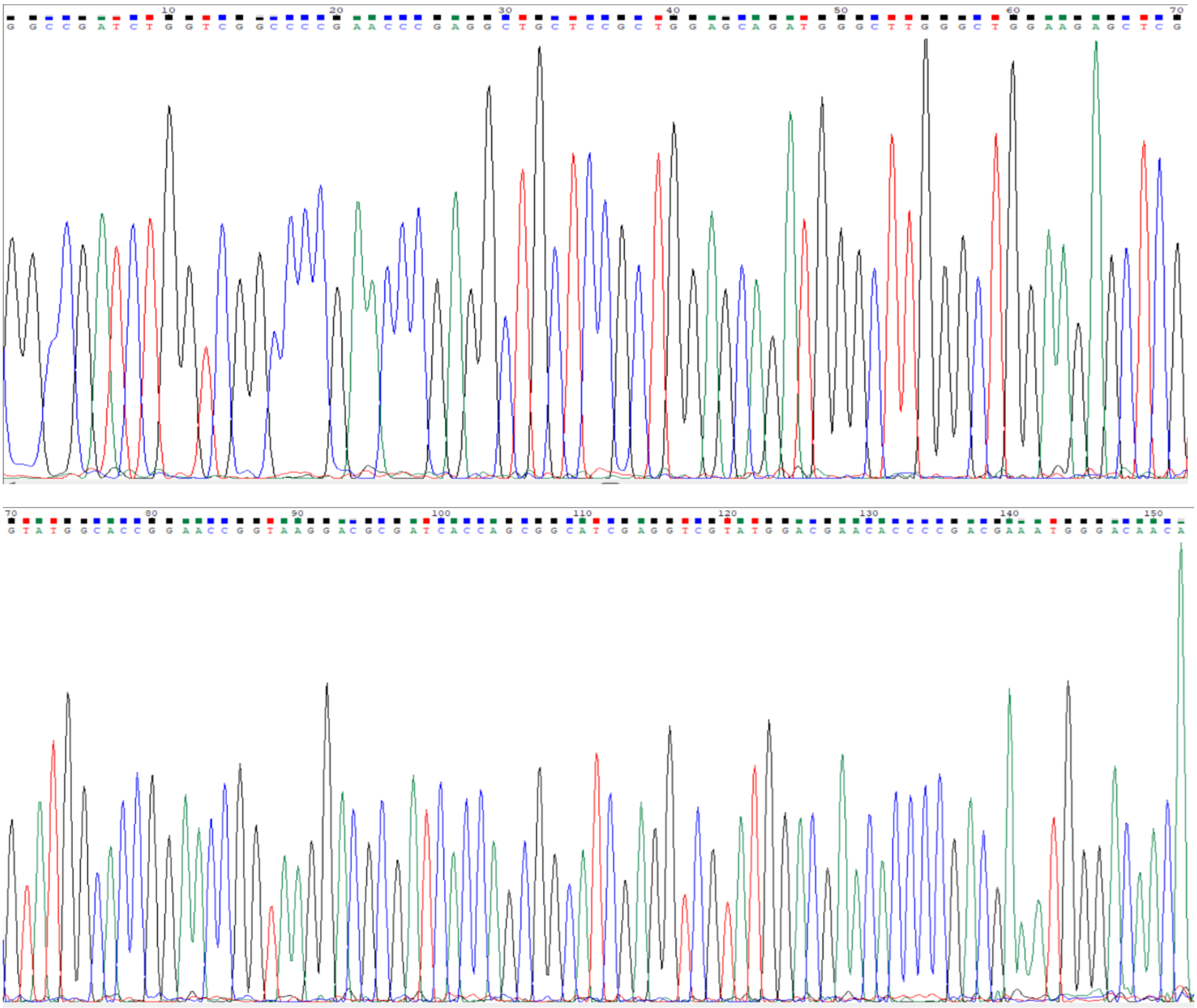

B18. Sequencing chromatograms of the *katG* gene, covering nucleotide positions 10–150, show a region with no detected mutations.

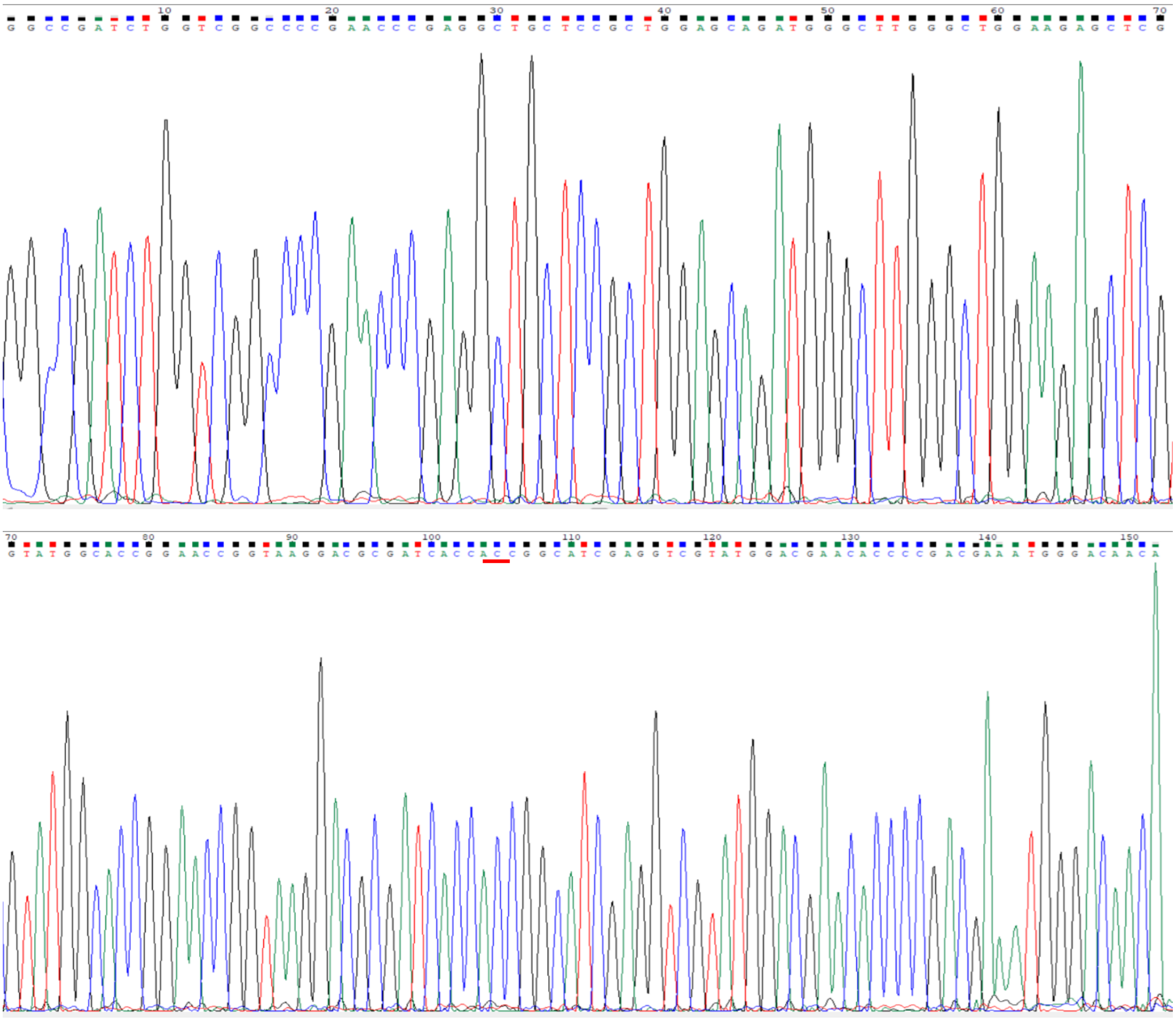

B19. Sequencing chromatograms of the *katG* gene, covering nucleotide positions 10–150, show an **AGC→ACC** substitution between nucleotide positions 100–110.

Chromatogram images of the *inhA* gene region from 19 isolates (C1-C19).

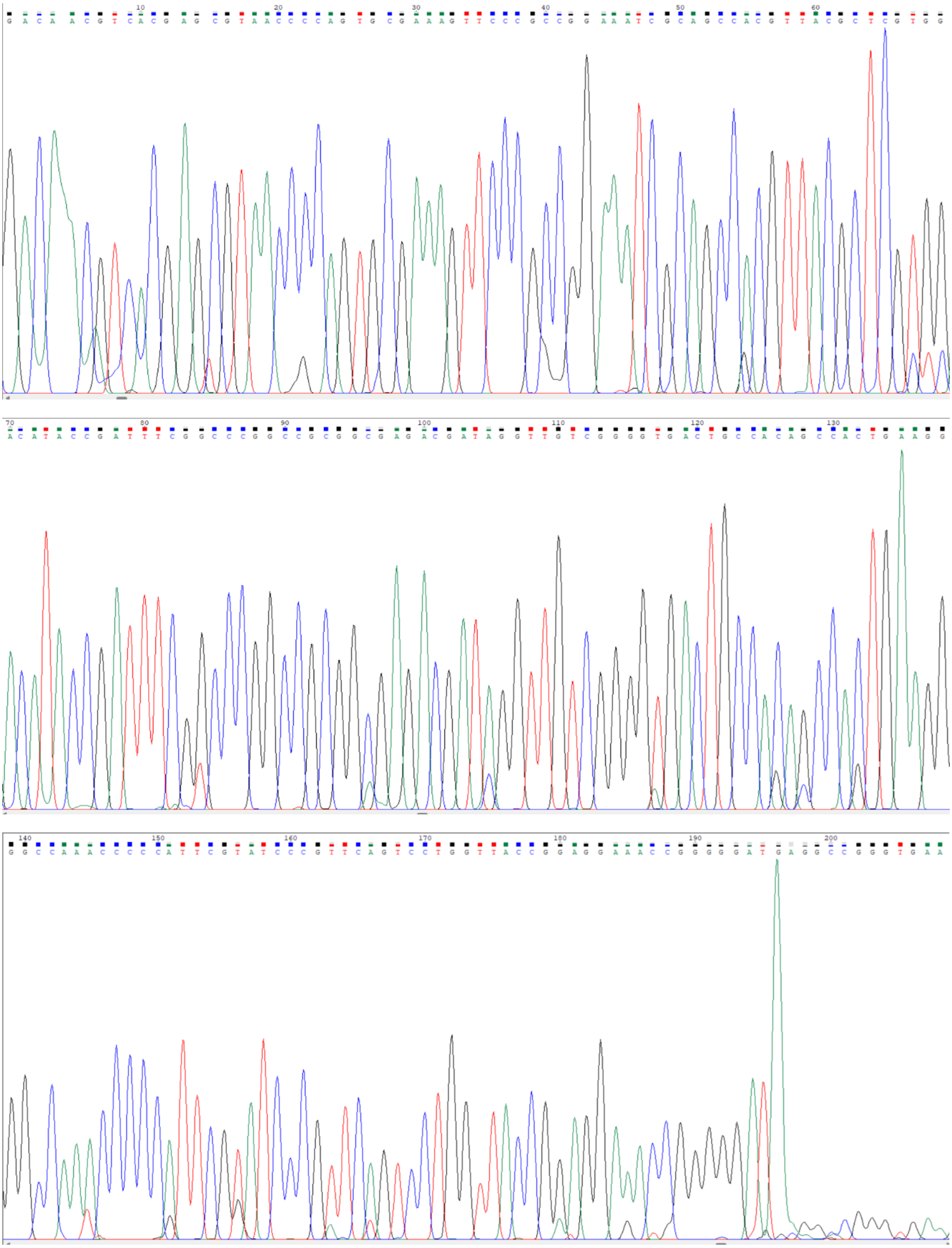

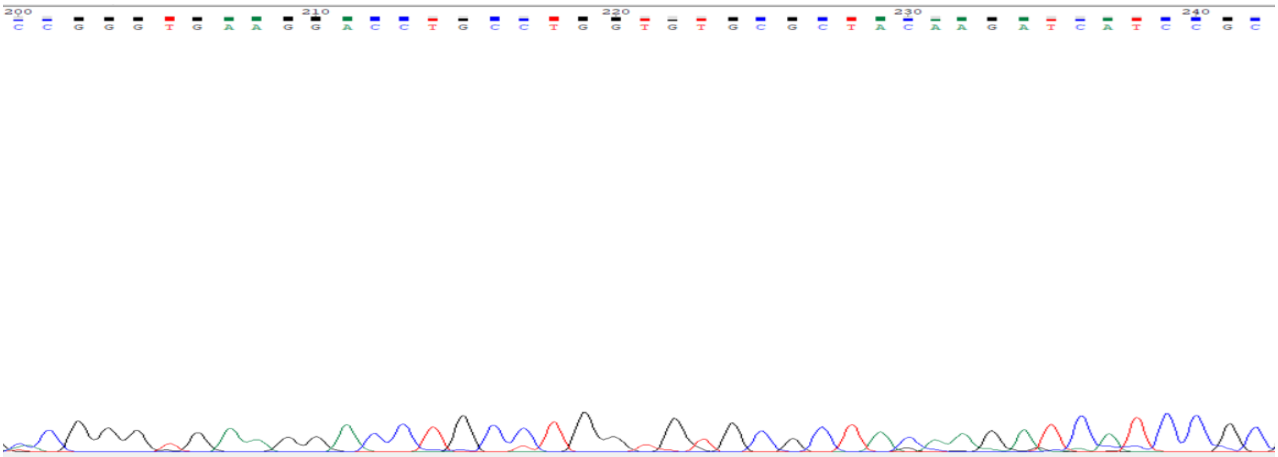

C1. Sequencing chromatograms of the *inhA* gene, covering nucleotide positions 10–240, show a region with no detected mutations.

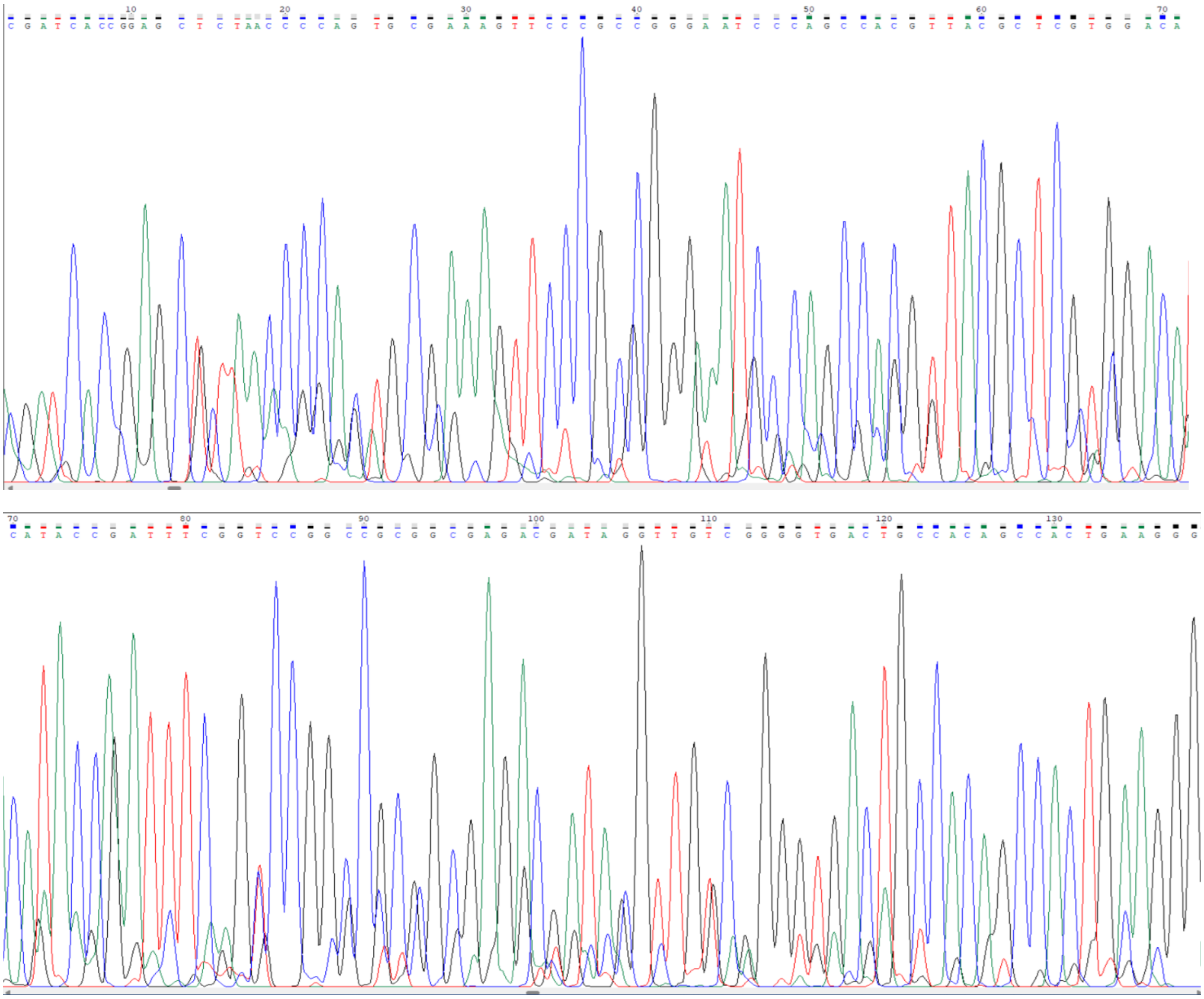

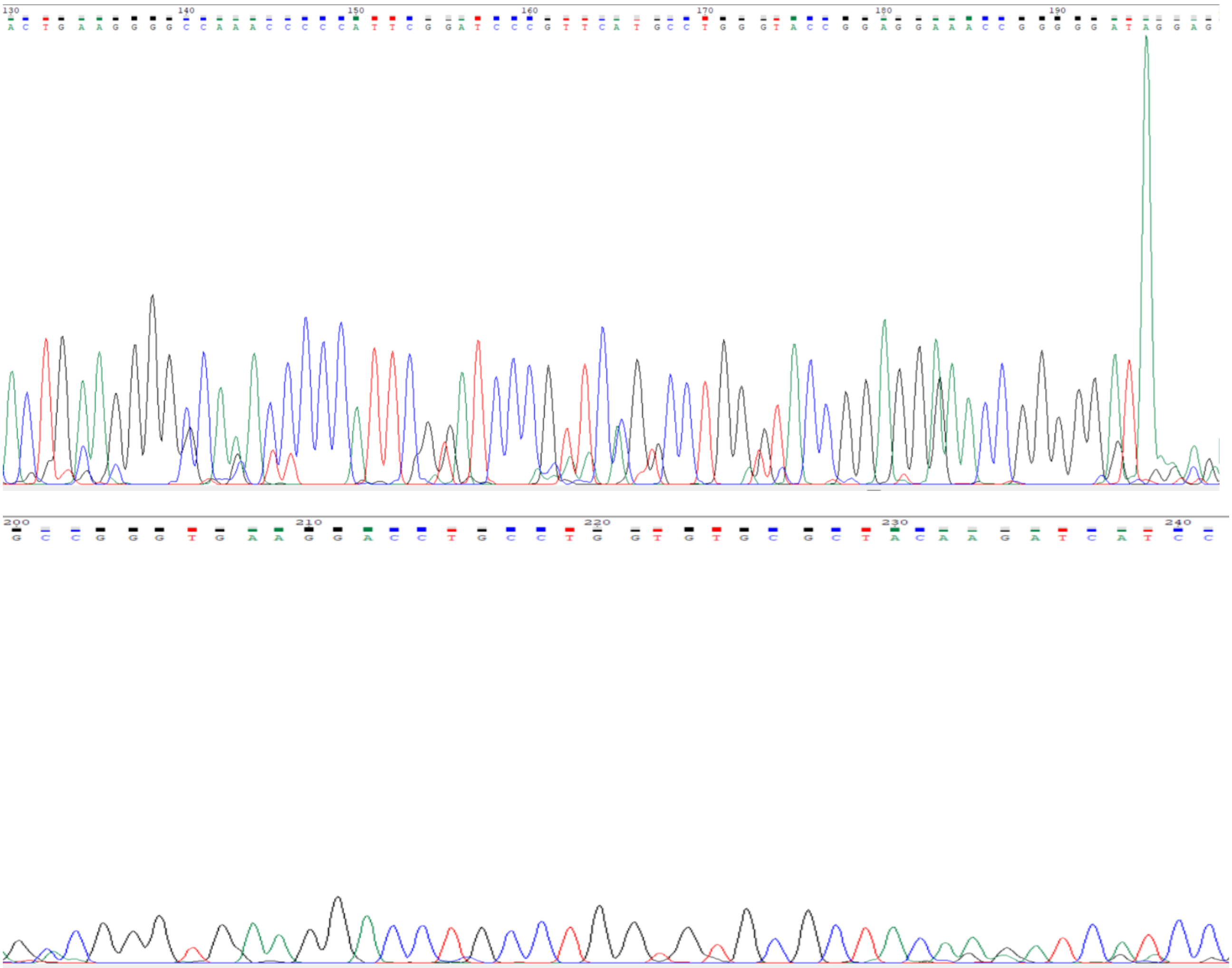

C2. Sequencing chromatograms of the *inhA* gene, covering nucleotide positions 10–240, show a region with no detected mutations.

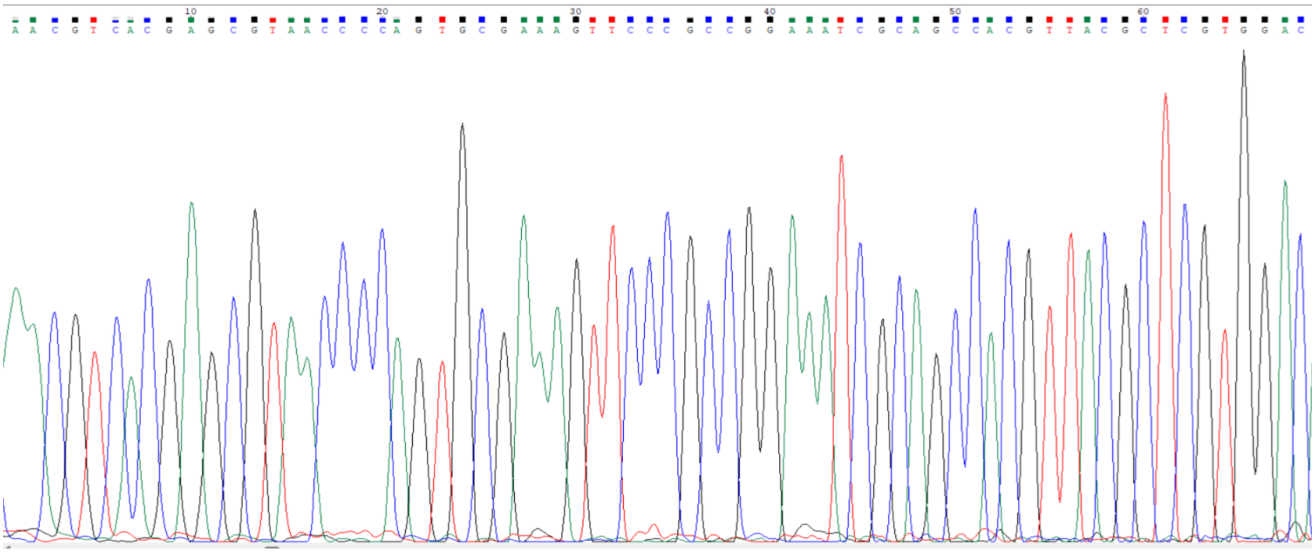

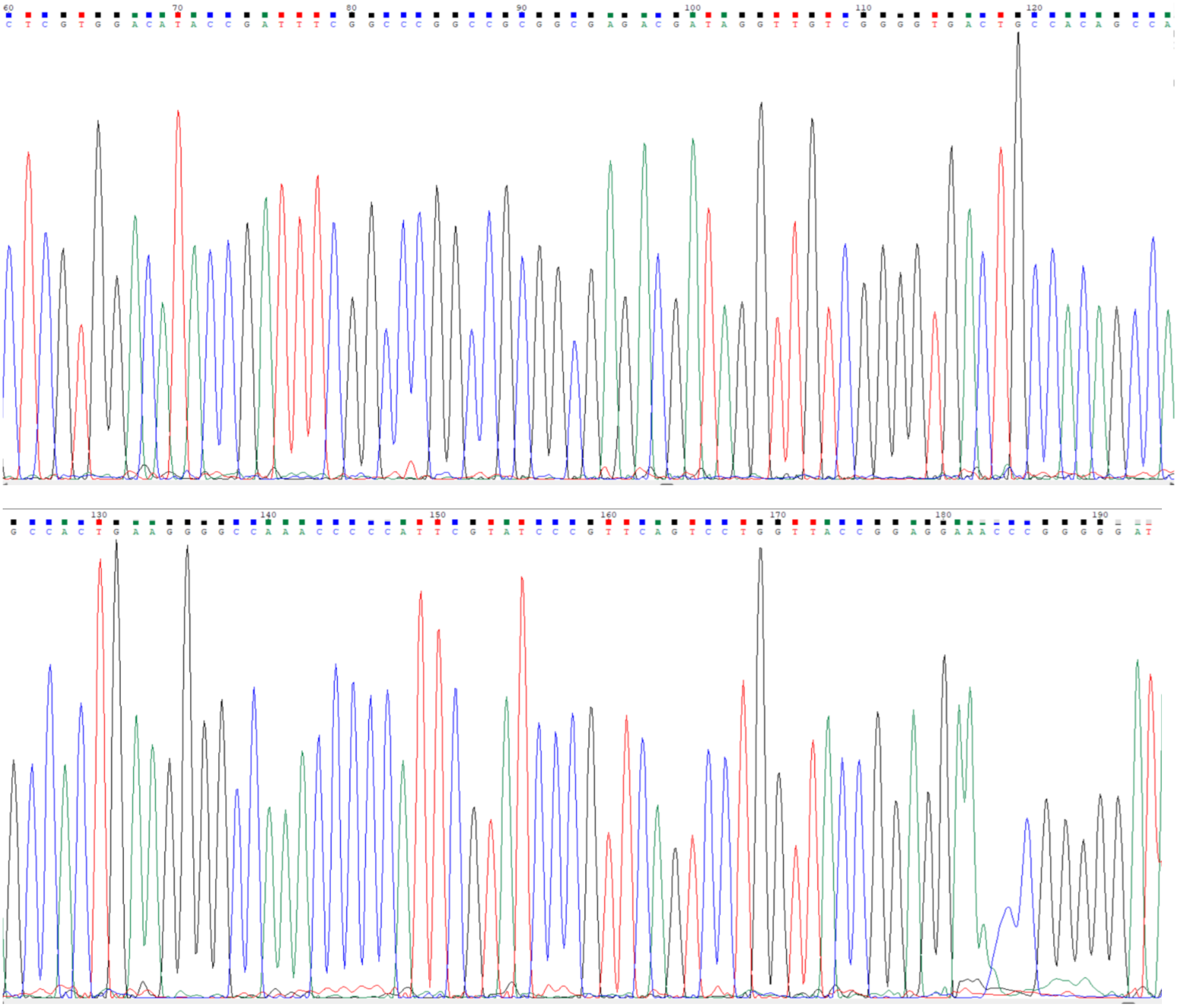

C3. Sequencing chromatograms of the *inhA* gene, covering nucleotide positions 10–190, show a region with no detected mutations.

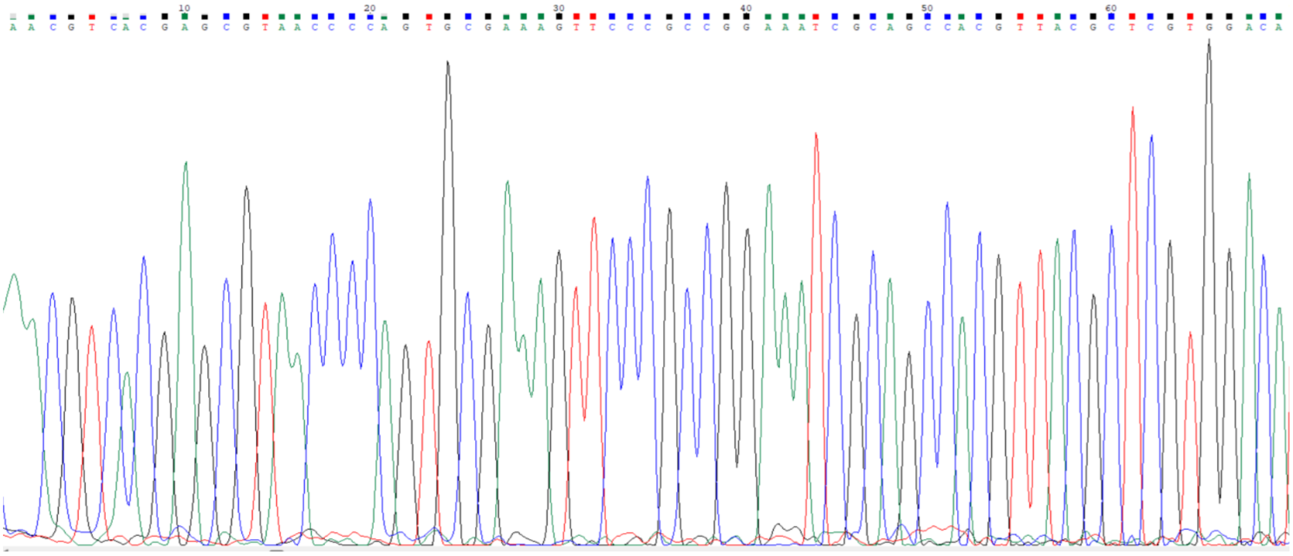

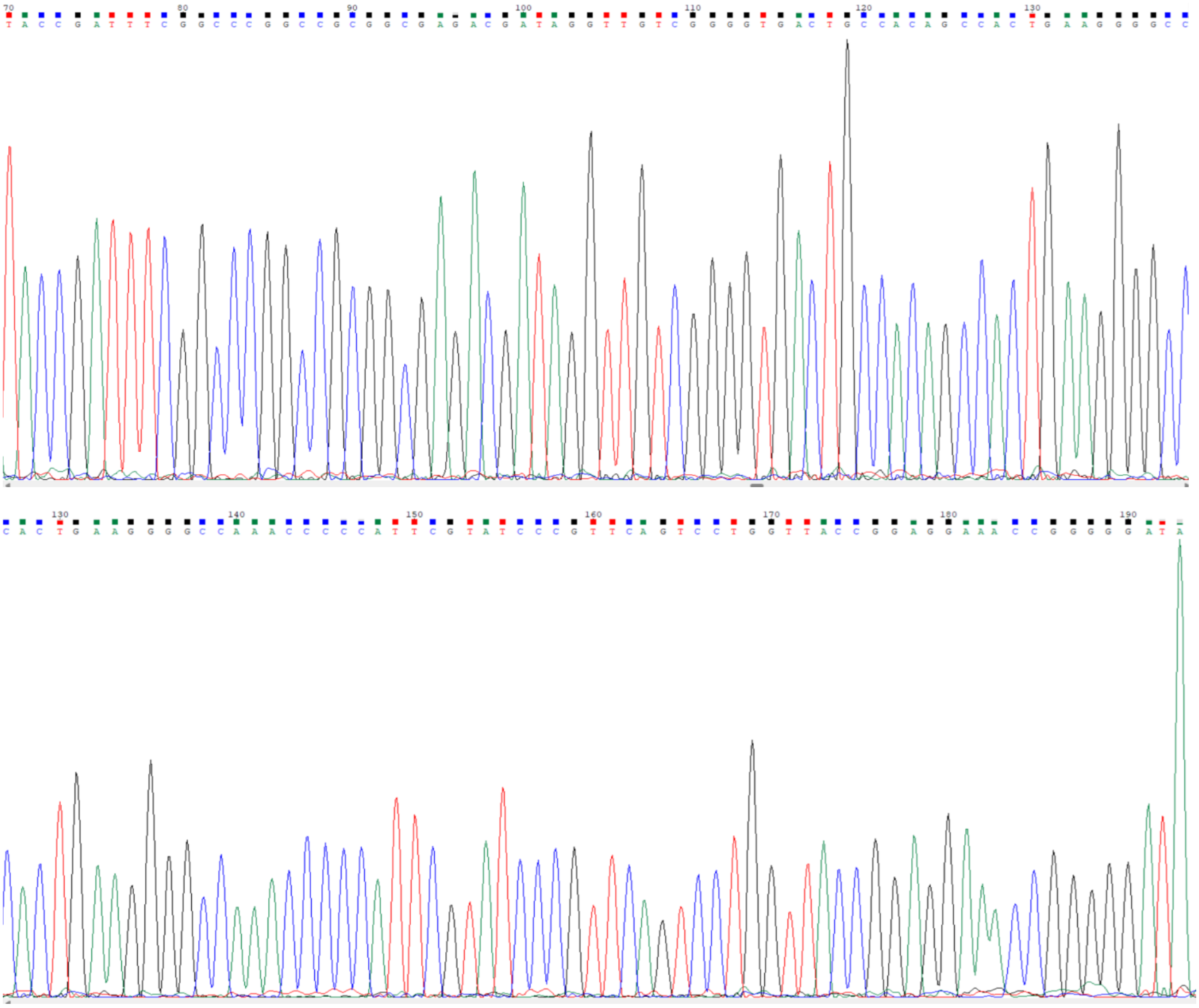

C4. Sequencing chromatograms of the *inhA* gene, covering nucleotide positions 10–190, show a region with no detected mutations.

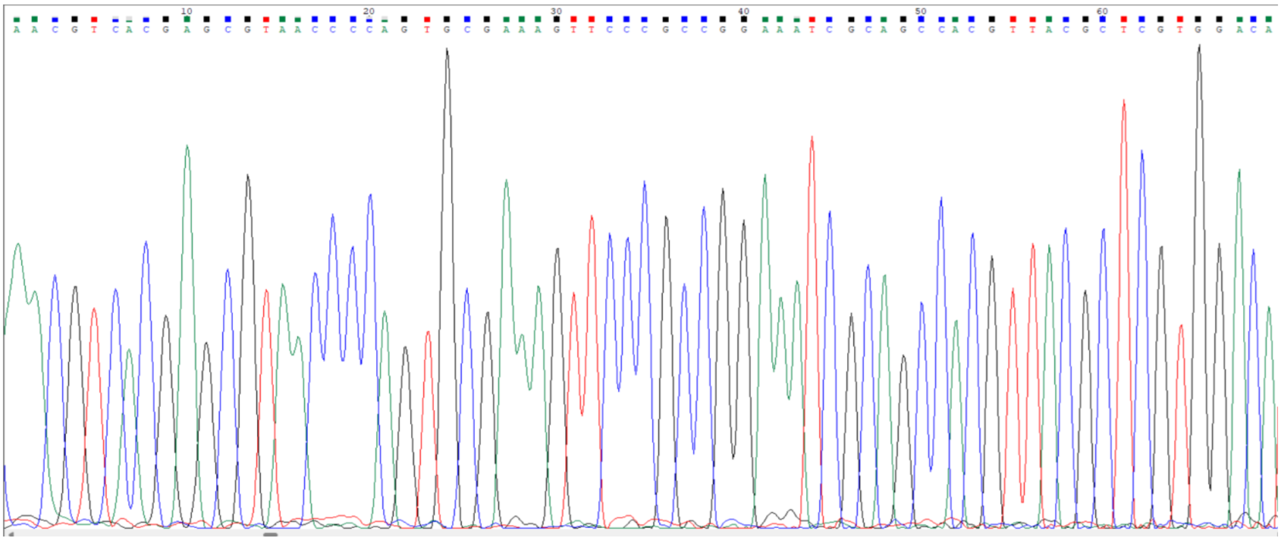

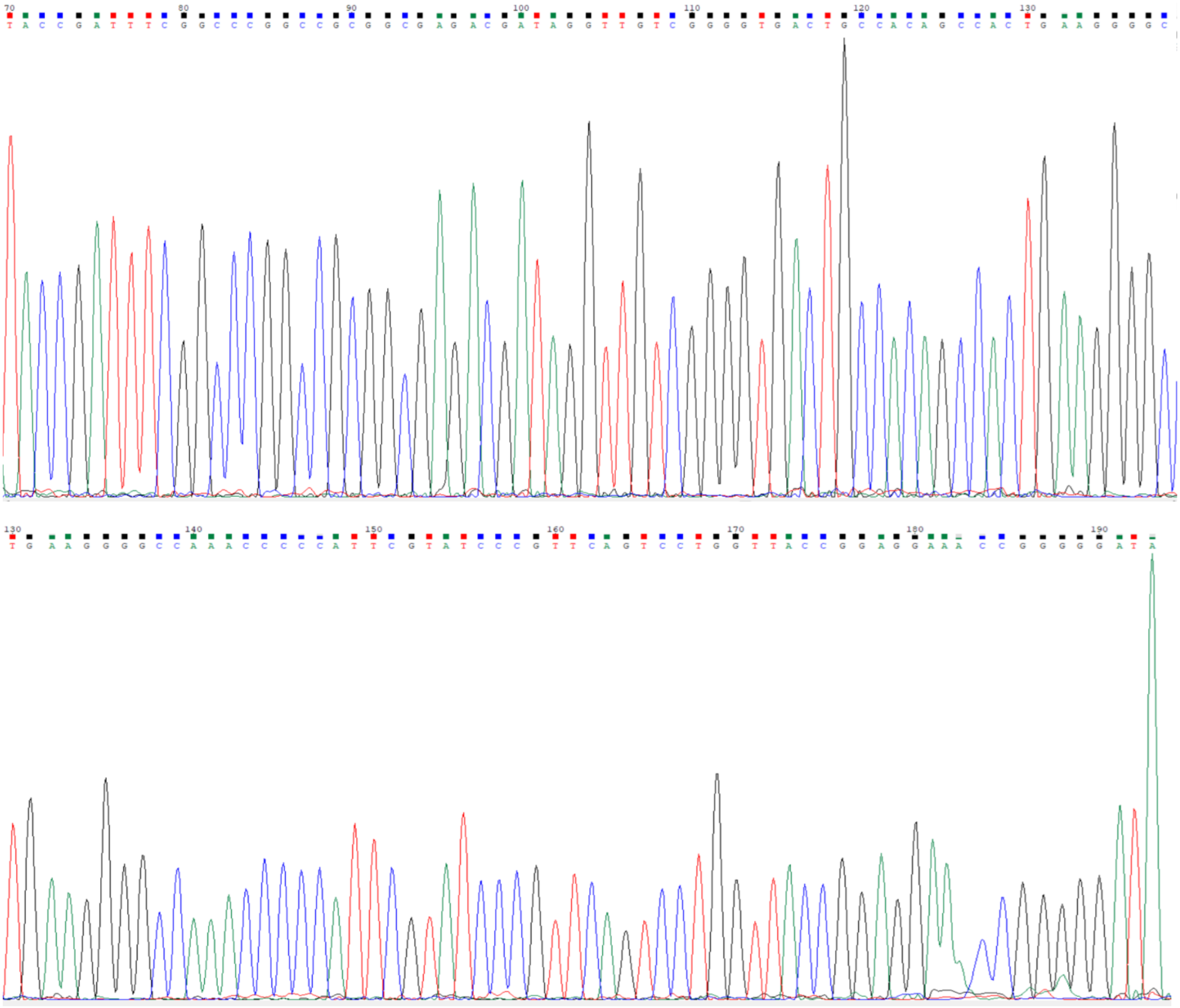

C5. Sequencing chromatograms of the *inhA* gene, covering nucleotide positions 10–190, show a region with no detected mutations.

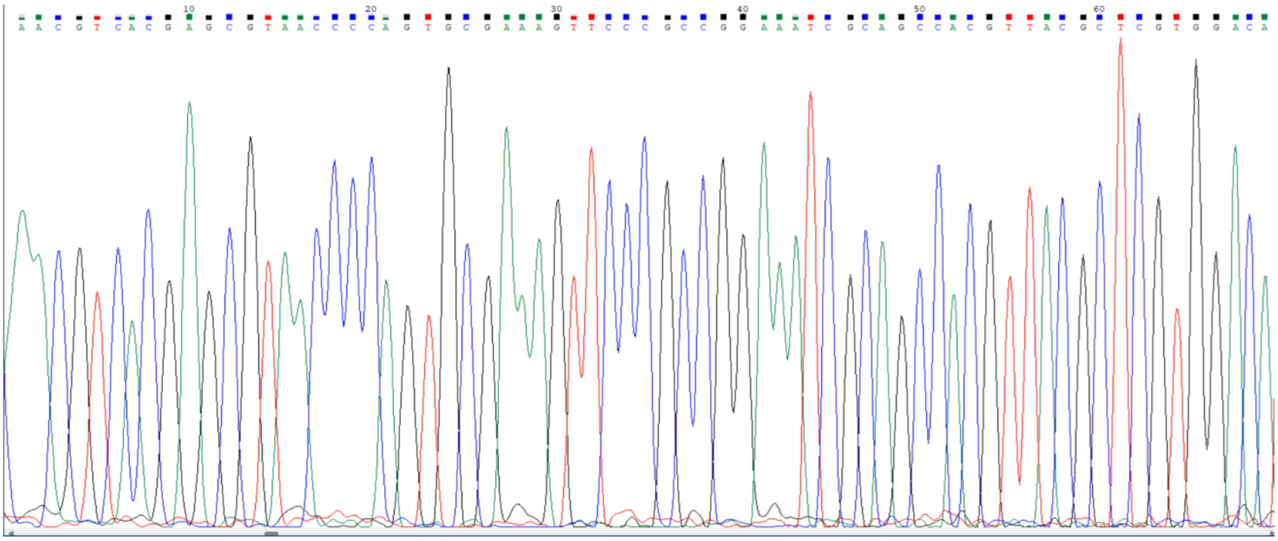

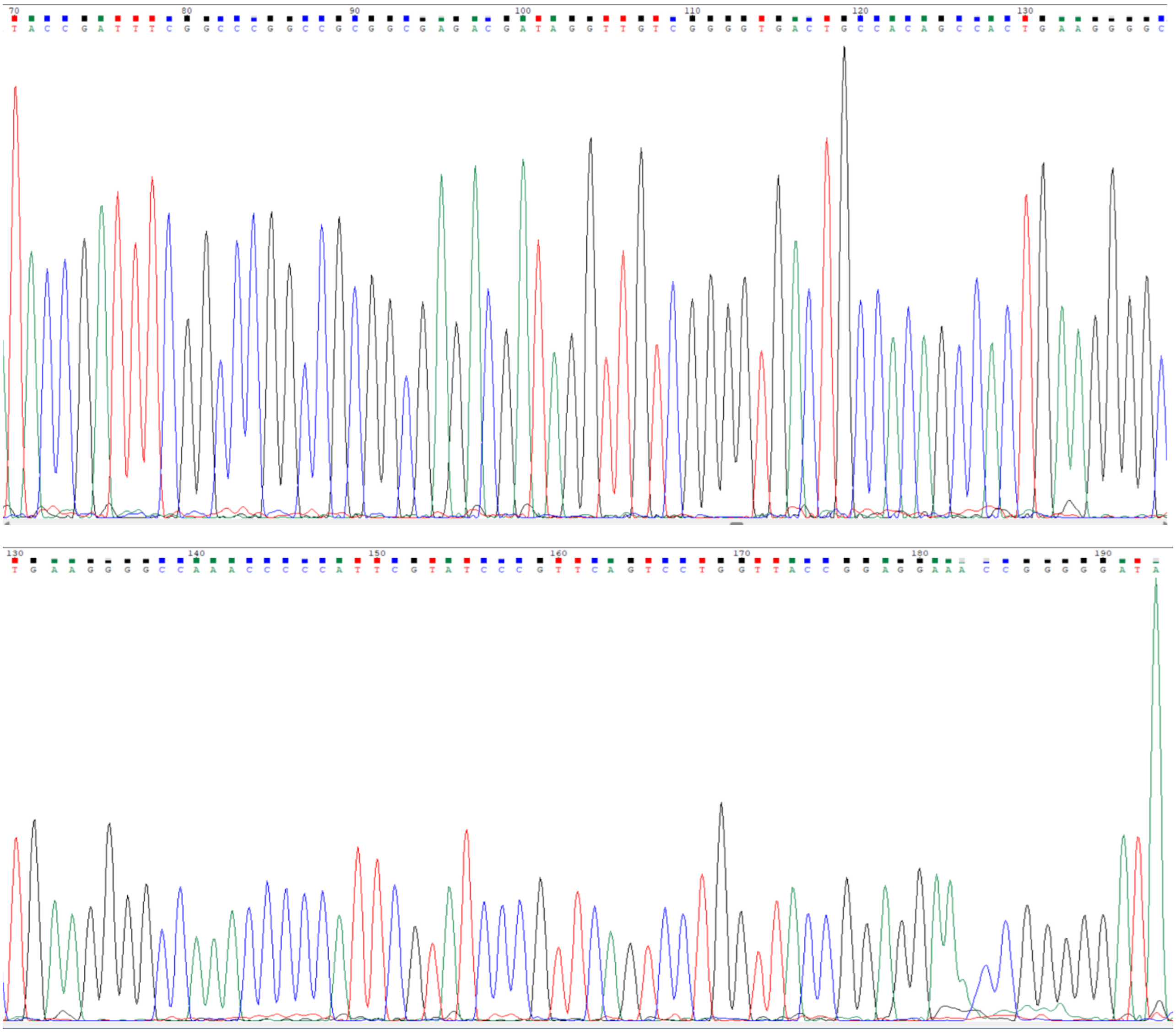

C6. Sequencing chromatograms of the *inhA* gene, covering nucleotide positions 10–190, show a region with no detected mutations.

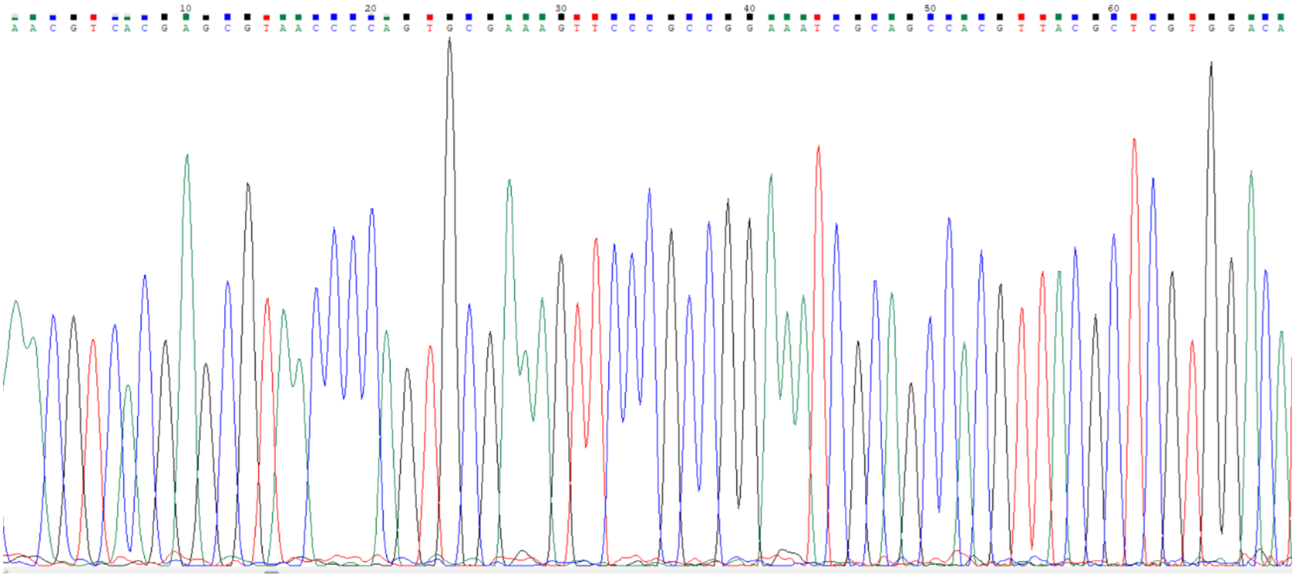

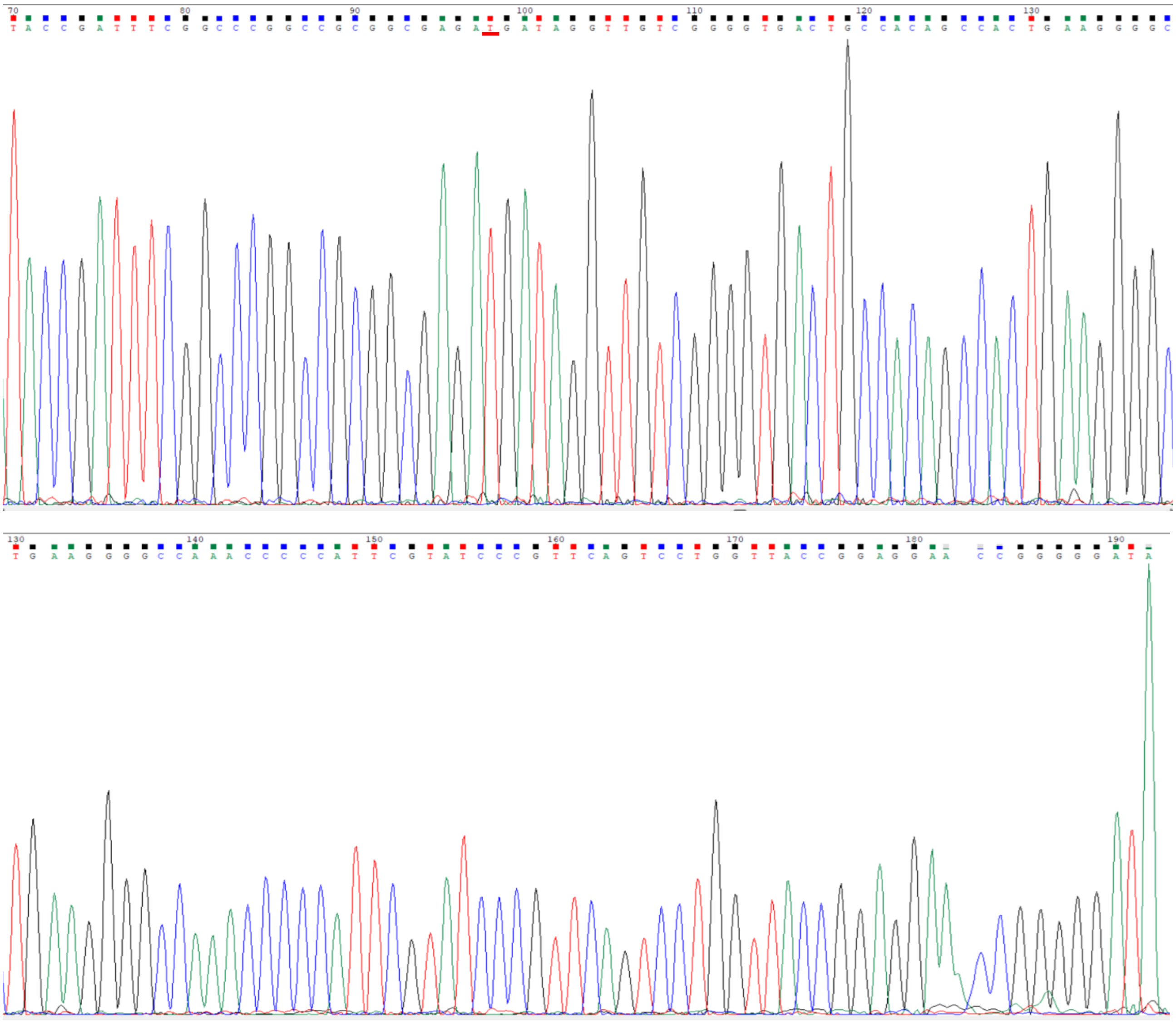

C7. Sequencing chromatograms of the *inhA* gene, covering nucleotide positions 10 –190, show an **C→T** substitution between nucleotide positions 90–100.

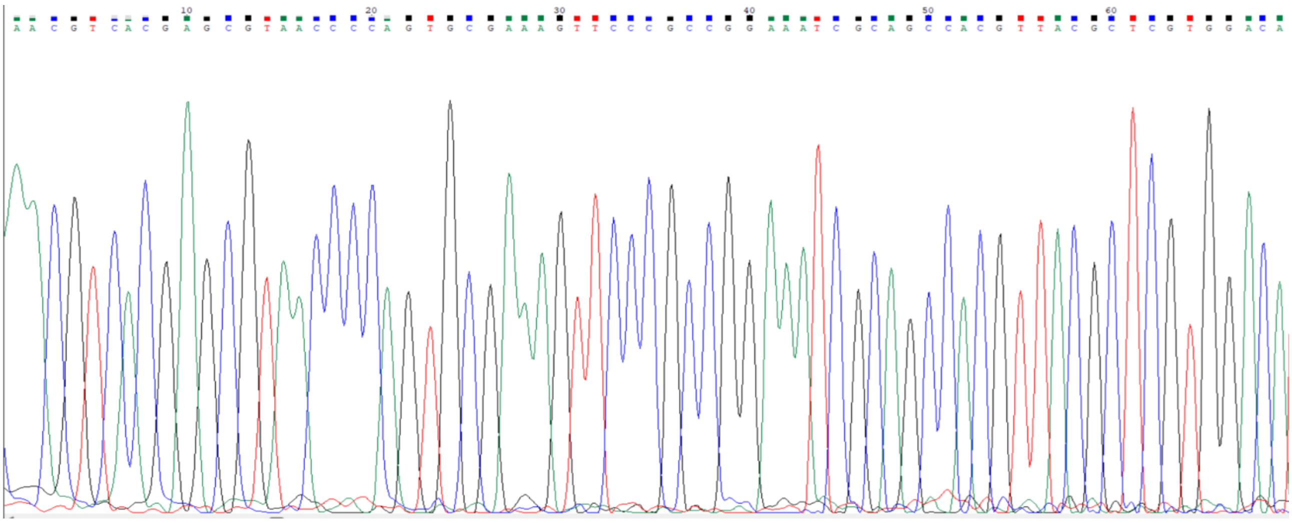

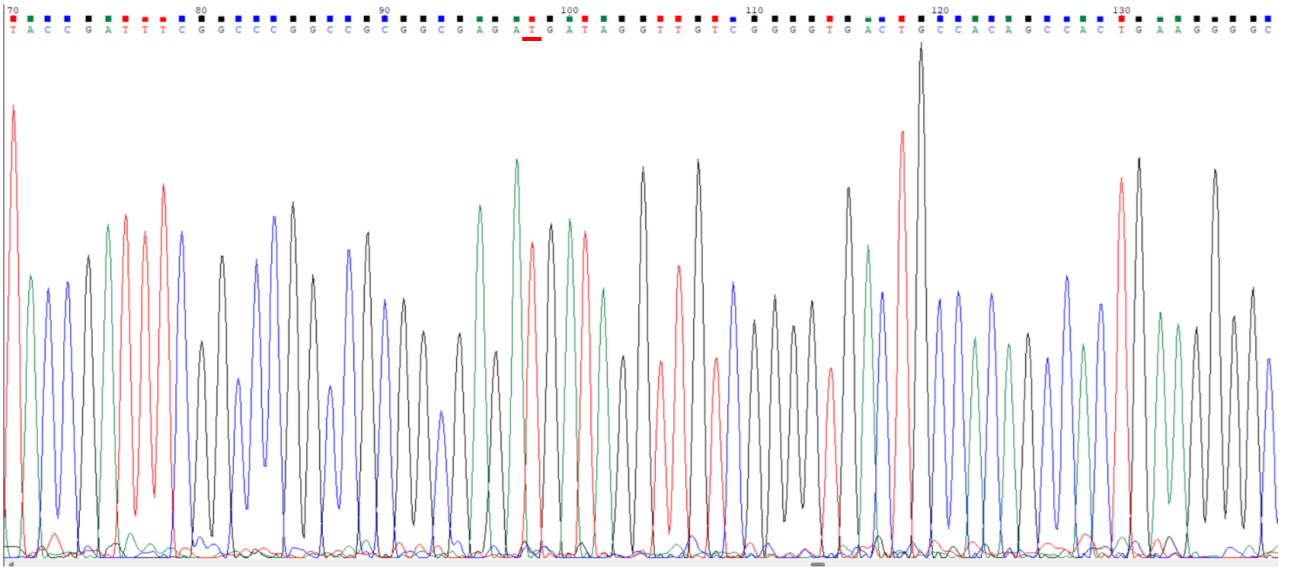

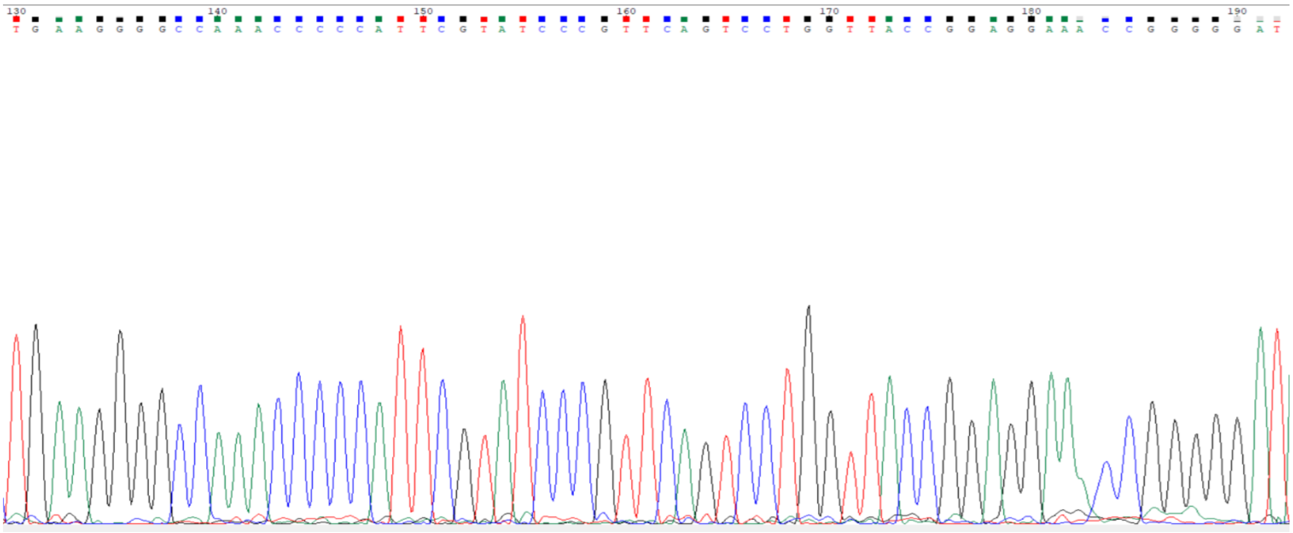

C8. Sequencing chromatograms of the *inhA* gene, covering nucleotide positions 10 –190, show an **C→T** substitution between nucleotide positions 90–100.

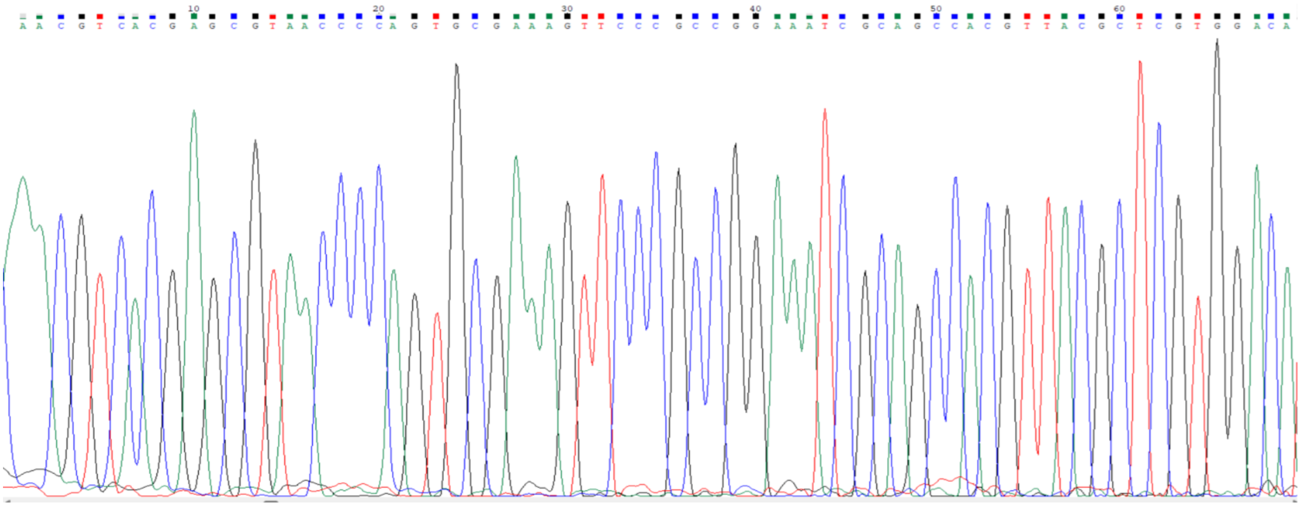

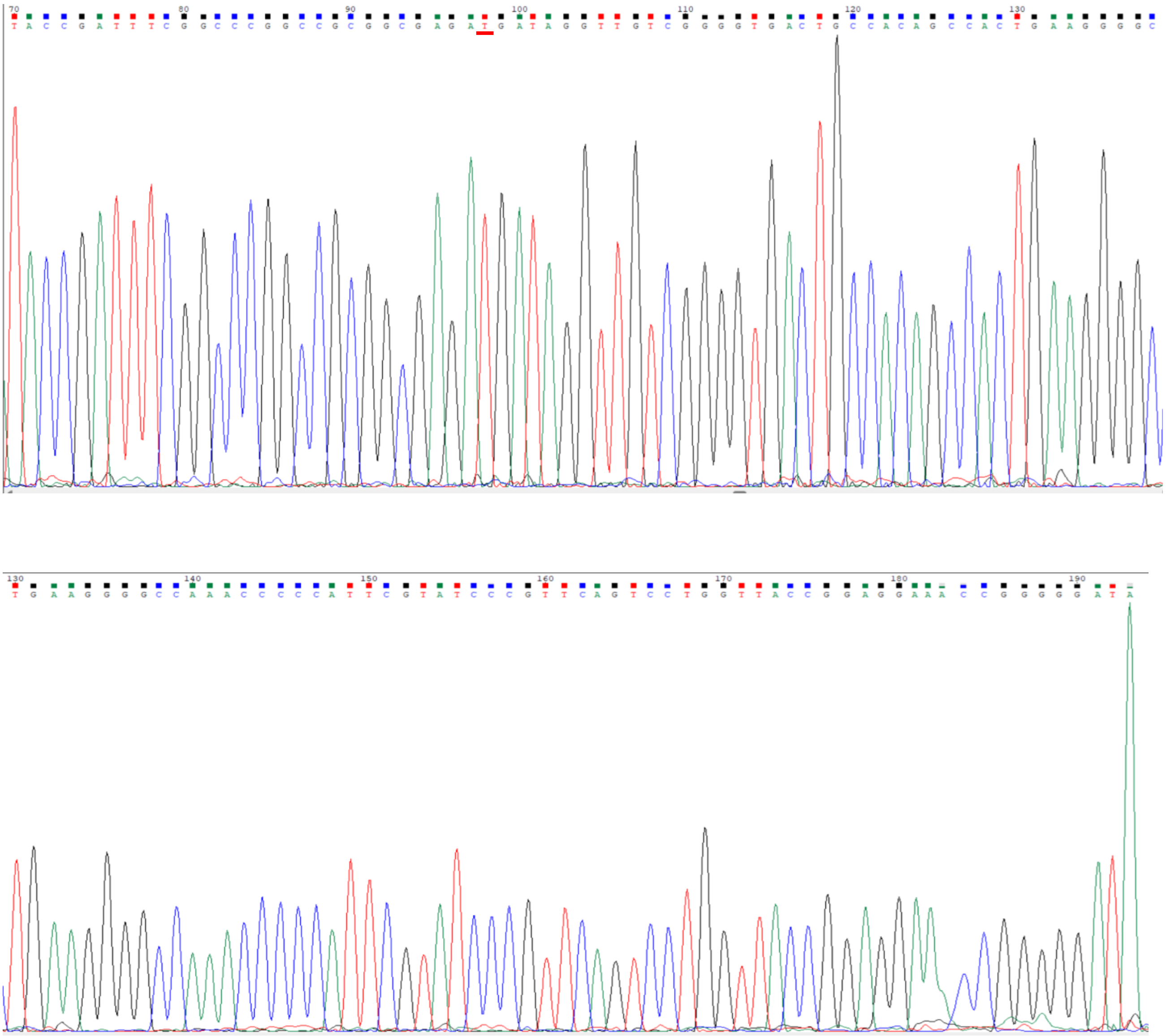

C9. Sequencing chromatograms of the *inhA* gene, covering nucleotide positions 10 –190, show an **C→T** substitution between nucleotide positions 90–100.

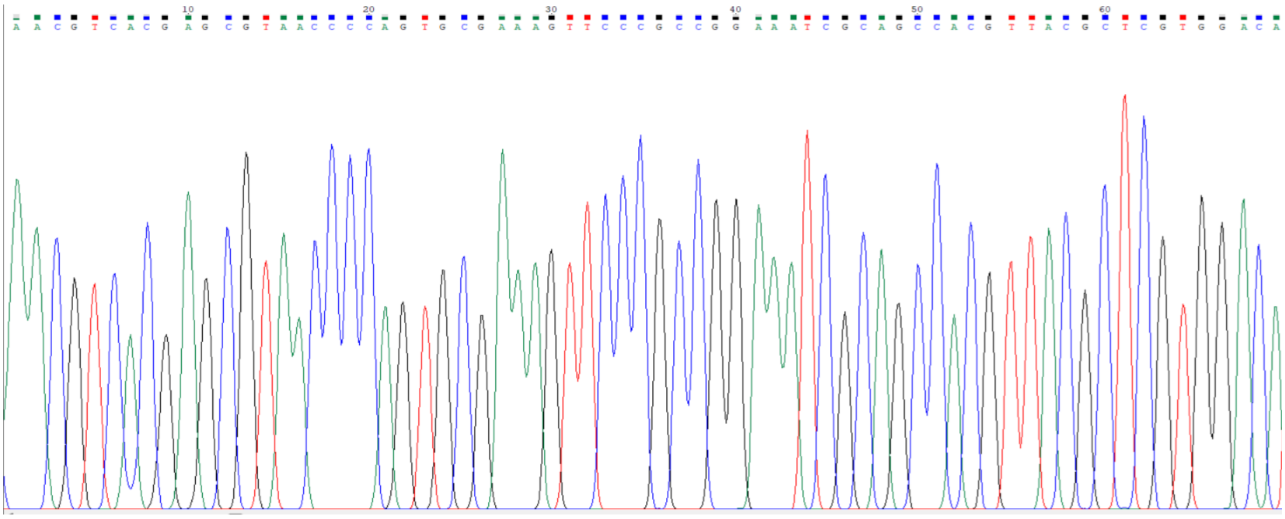

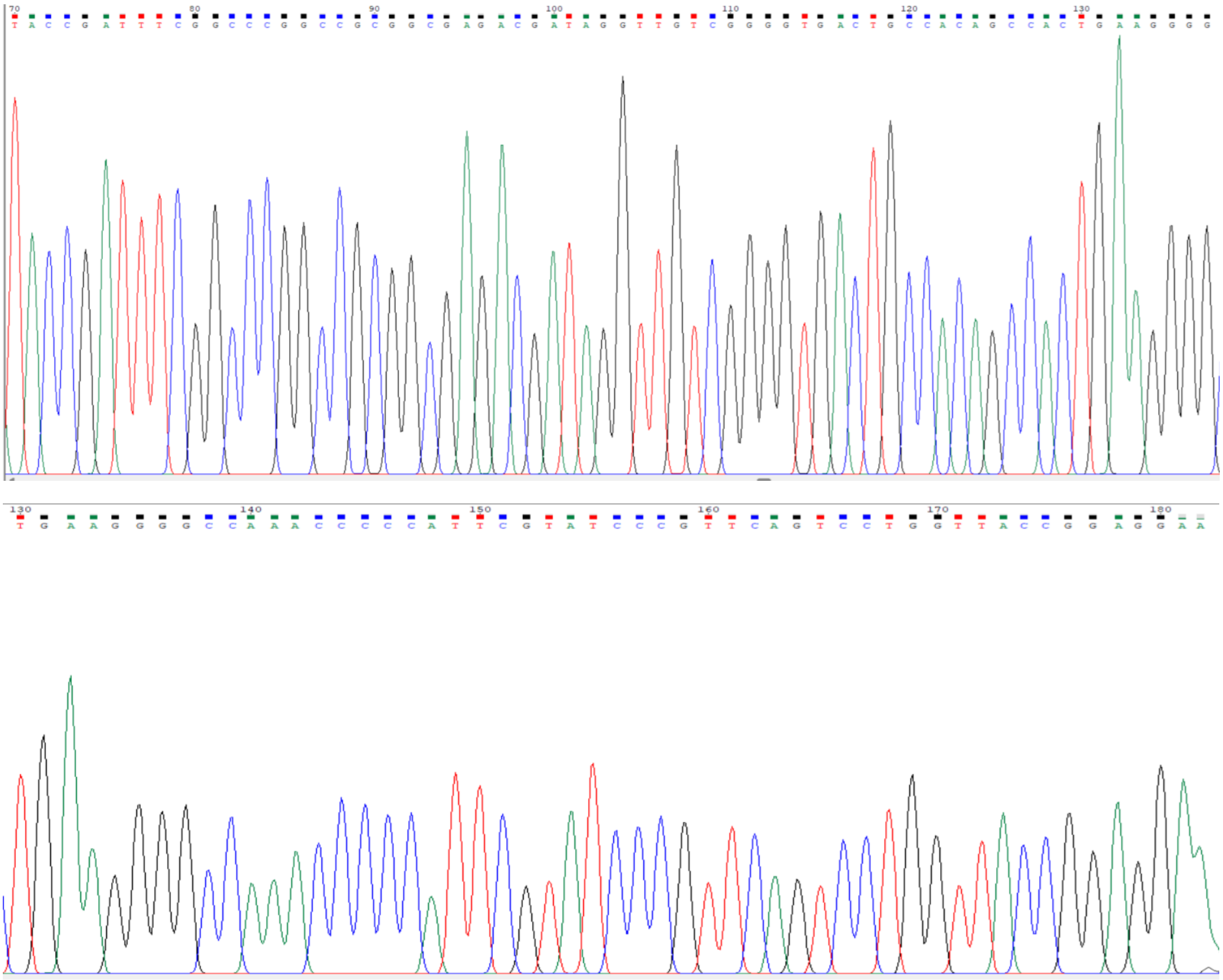

C10. Sequencing chromatograms of the *inhA* gene, covering nucleotide positions 10–180, show a region with no detected mutations.

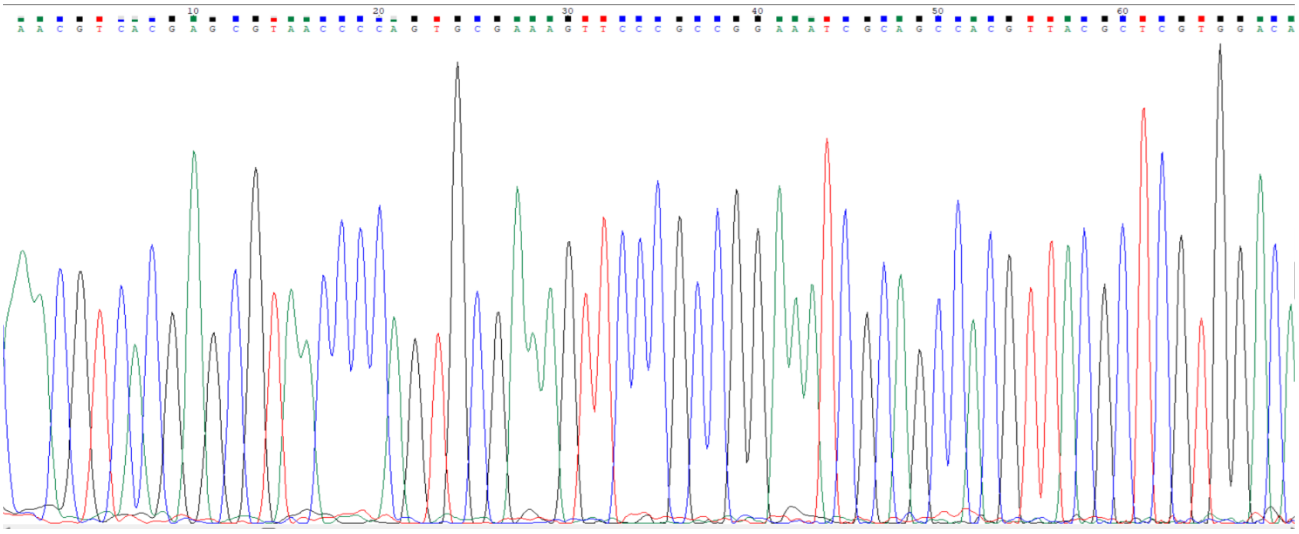

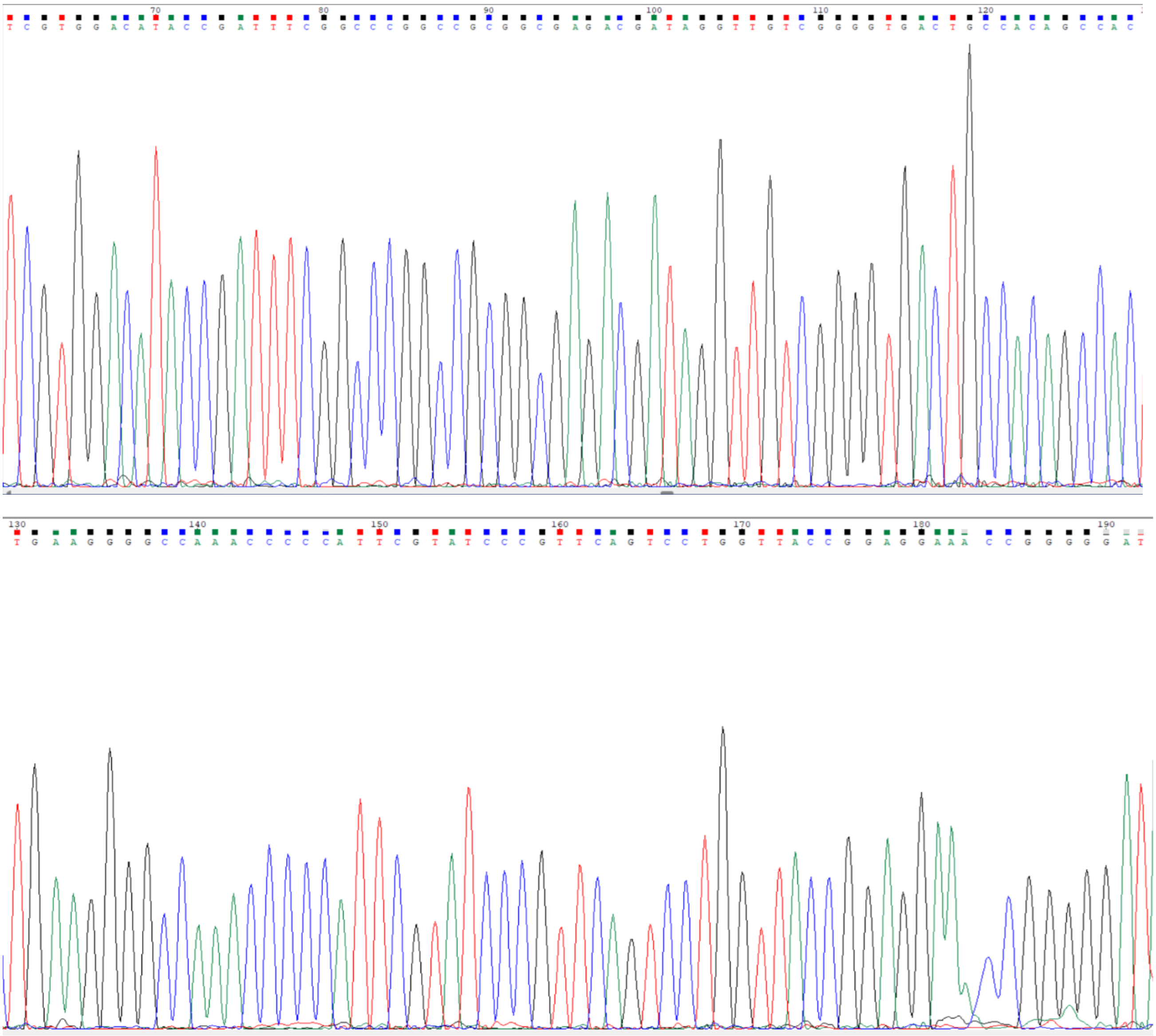

C11. Sequencing chromatograms of the *inhA* gene, covering nucleotide positions 10–190, show a region with no detected mutations.

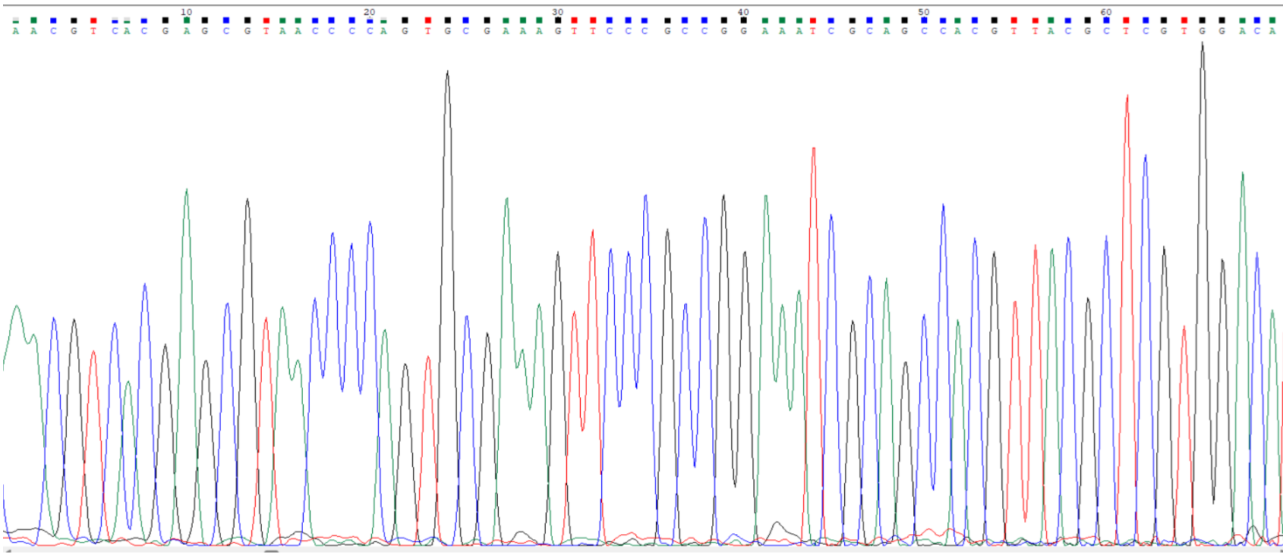

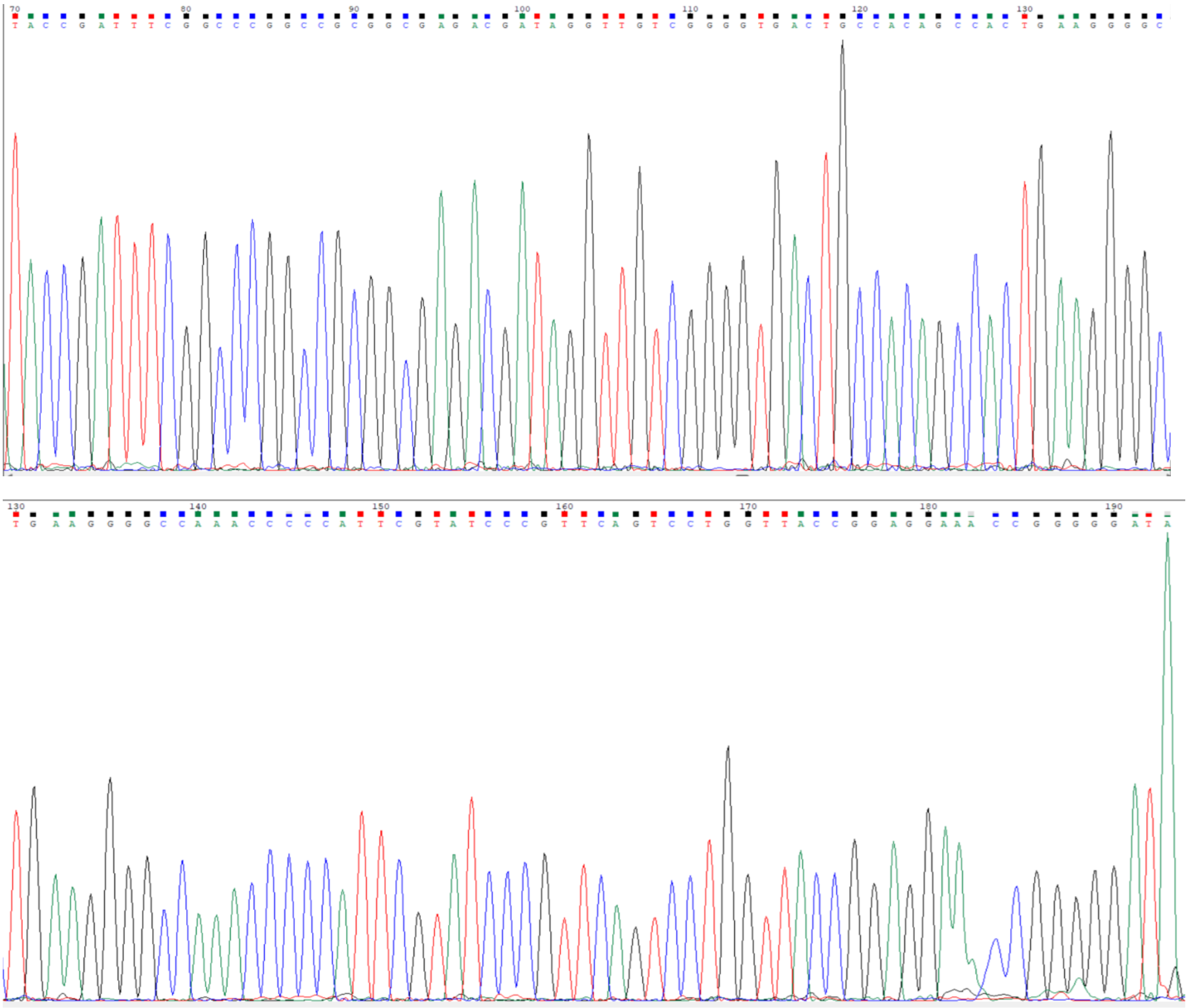

C12. Sequencing chromatograms of the *inhA* gene, covering nucleotide positions 10–190, show a region with no detected mutations.

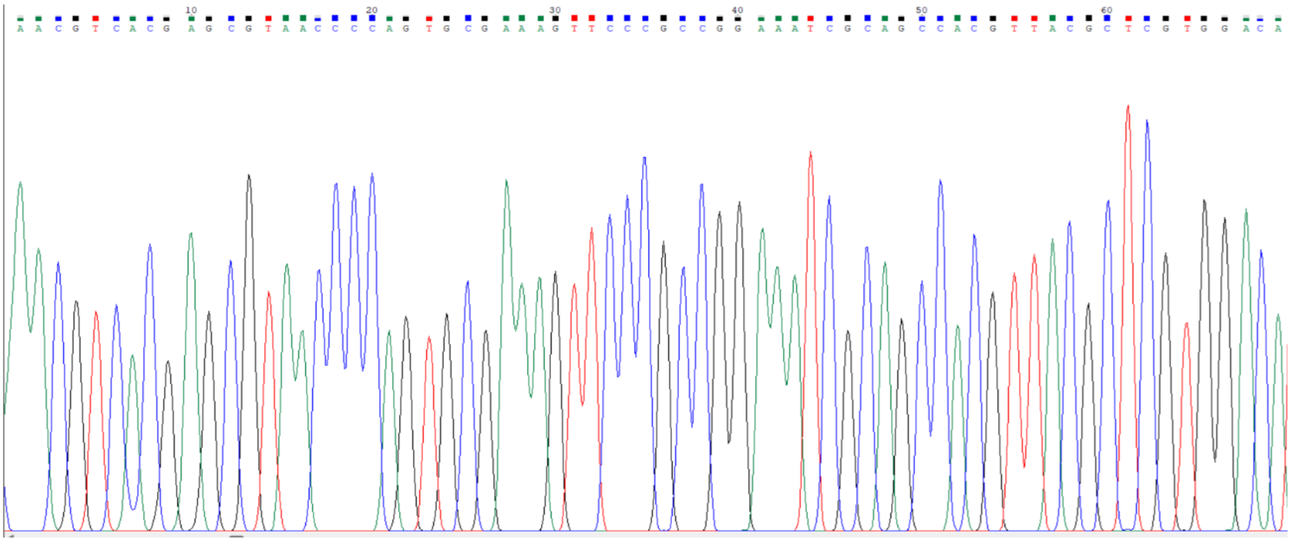

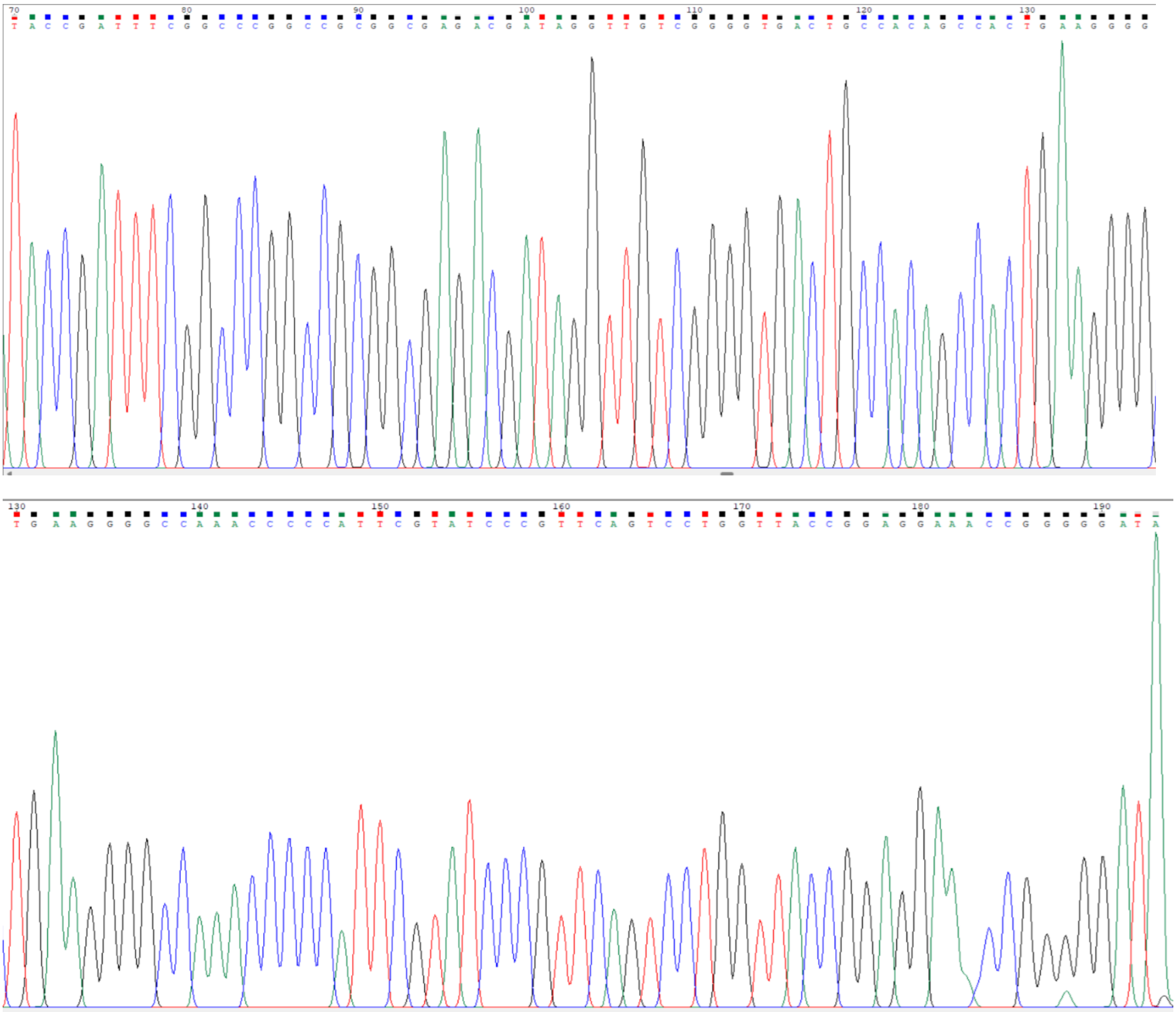

C13. Sequencing chromatograms of the *inhA* gene, covering nucleotide positions 10–190, show a region with no detected mutations.

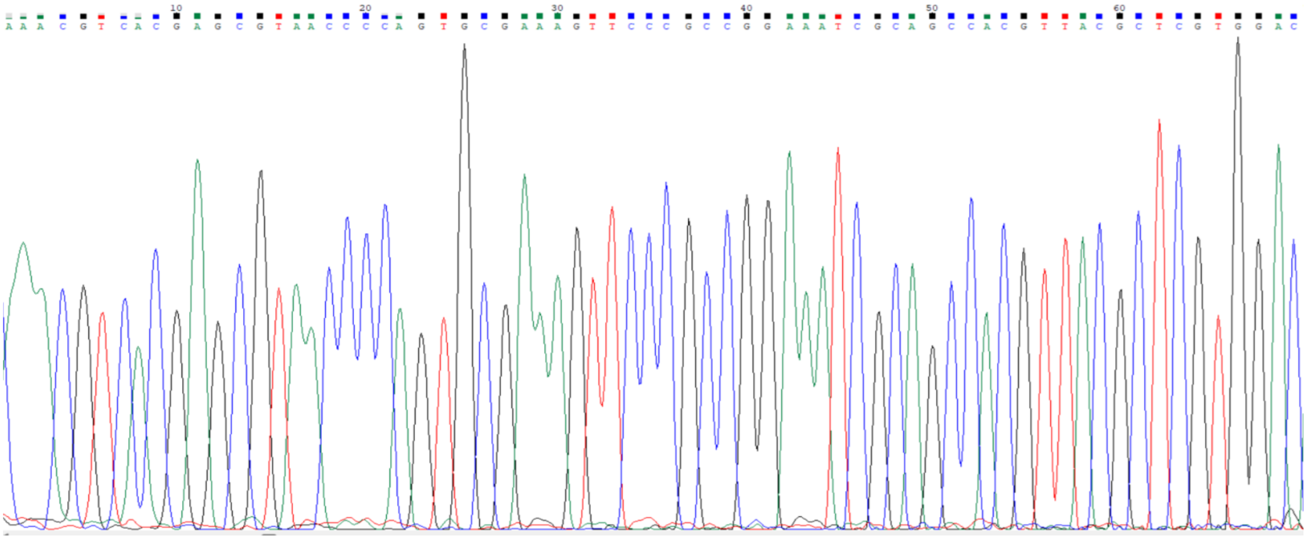

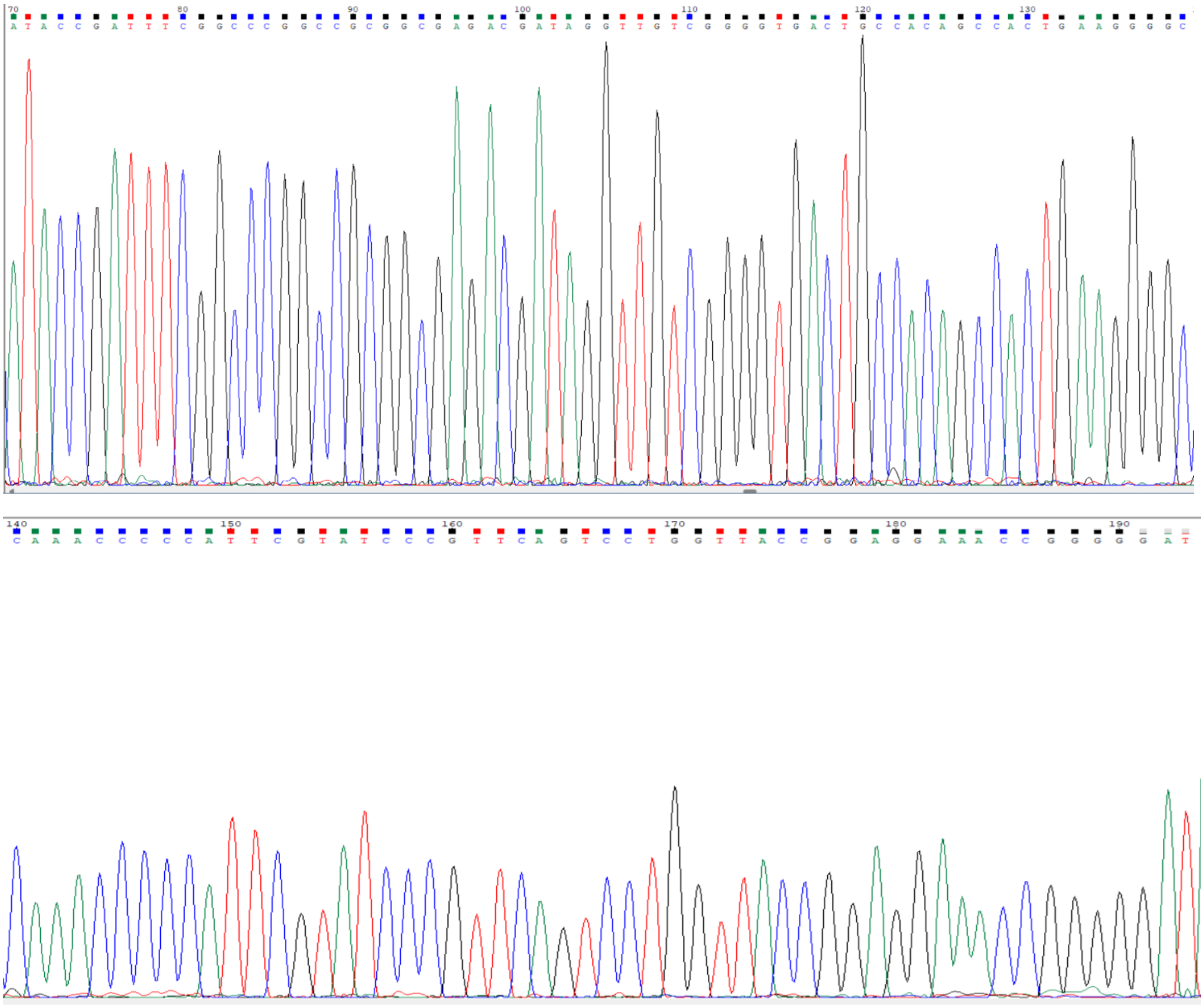

C14. Sequencing chromatograms of the *inhA* gene, covering nucleotide positions 10–190, show a region with no detected mutations.

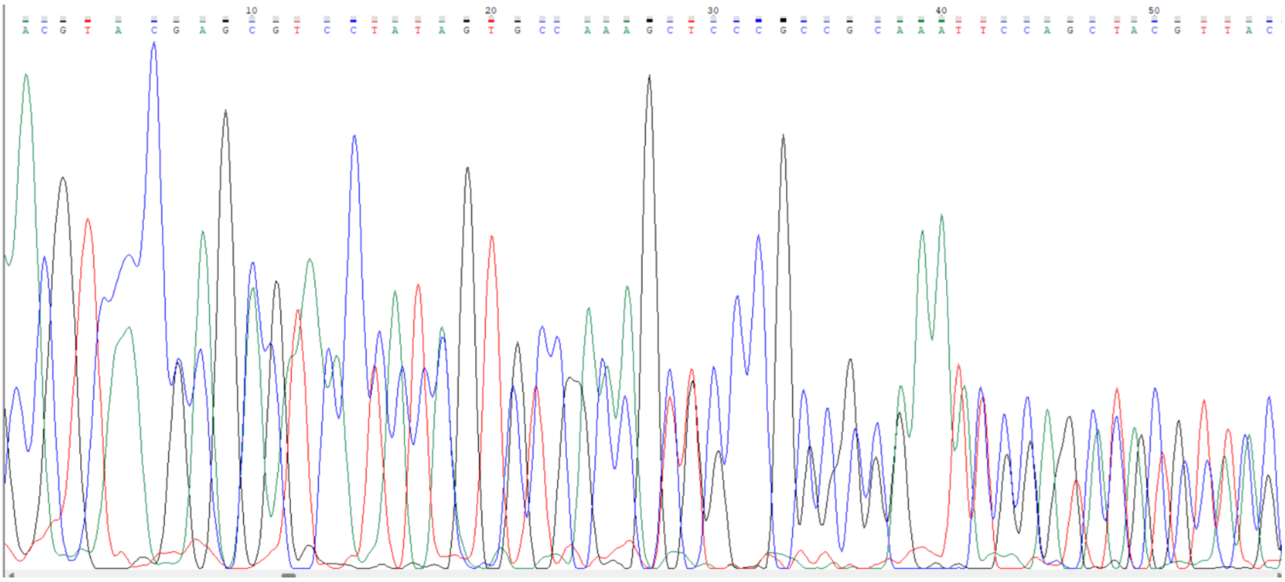

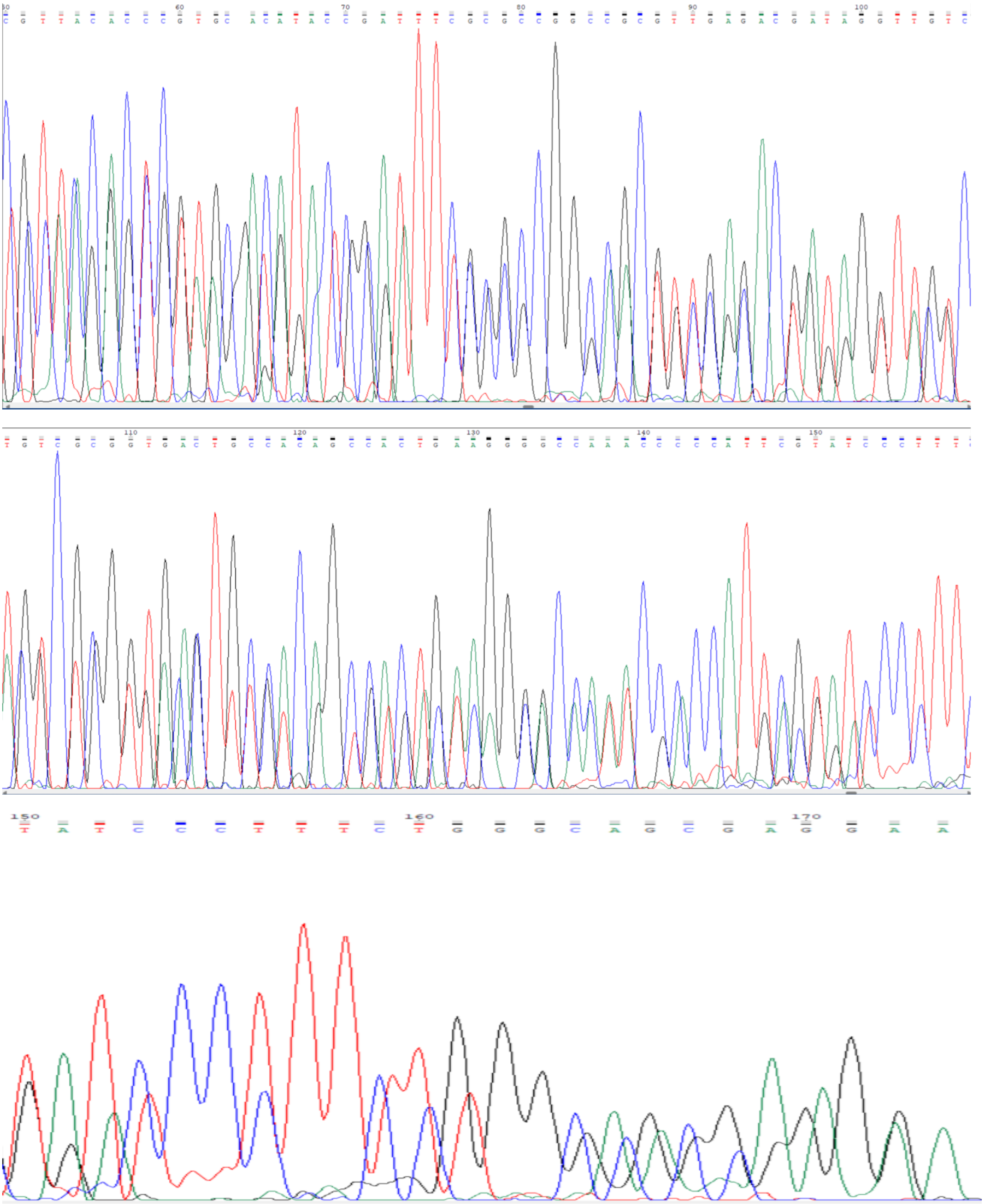

C15. Sequencing chromatograms of the *inhA* gene, covering nucleotide positions 10–170, show a region with no detected mutations.

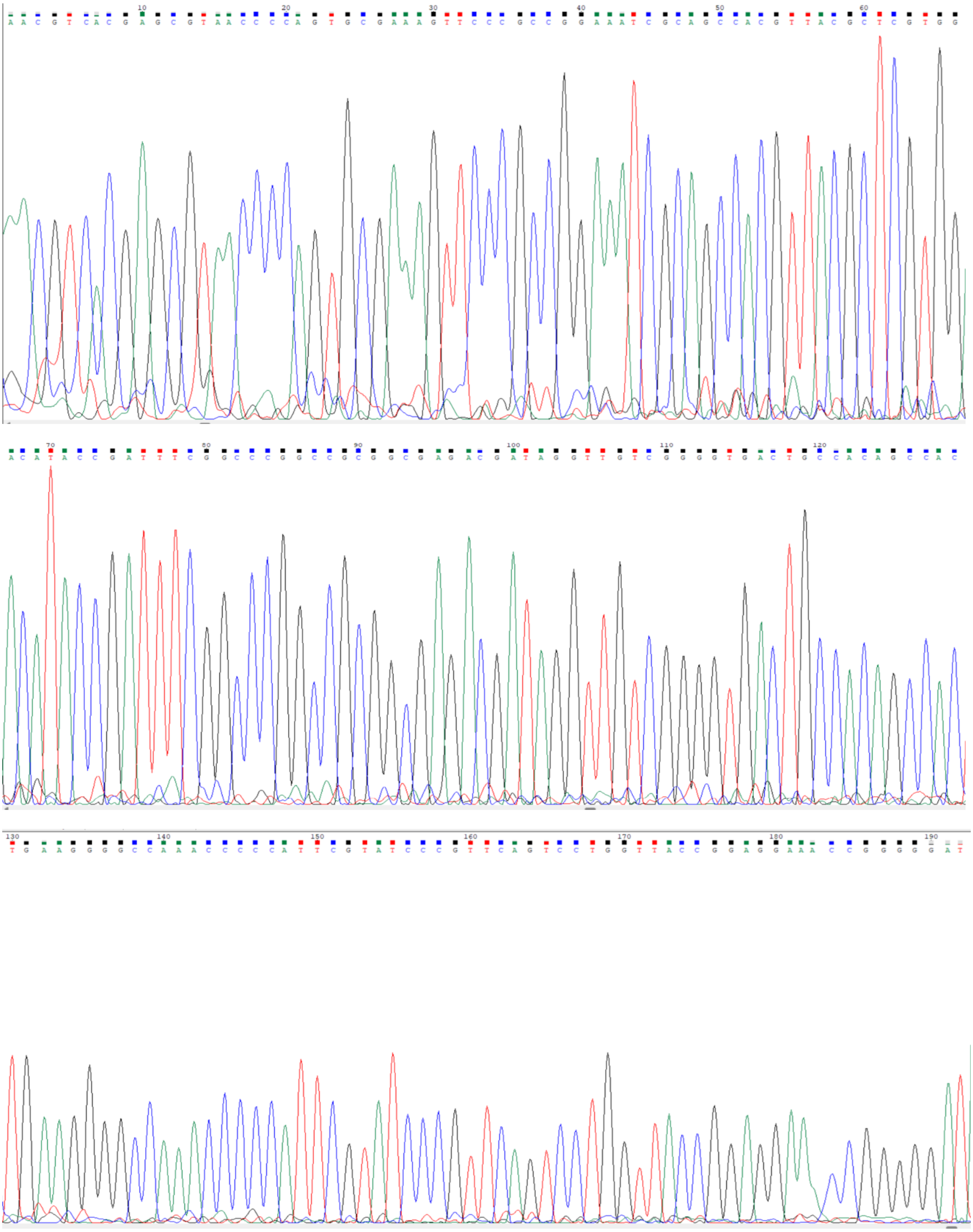

C16. Sequencing chromatograms of the *inhA* gene, covering nucleotide positions 10–190, show a region with no detected mutations.

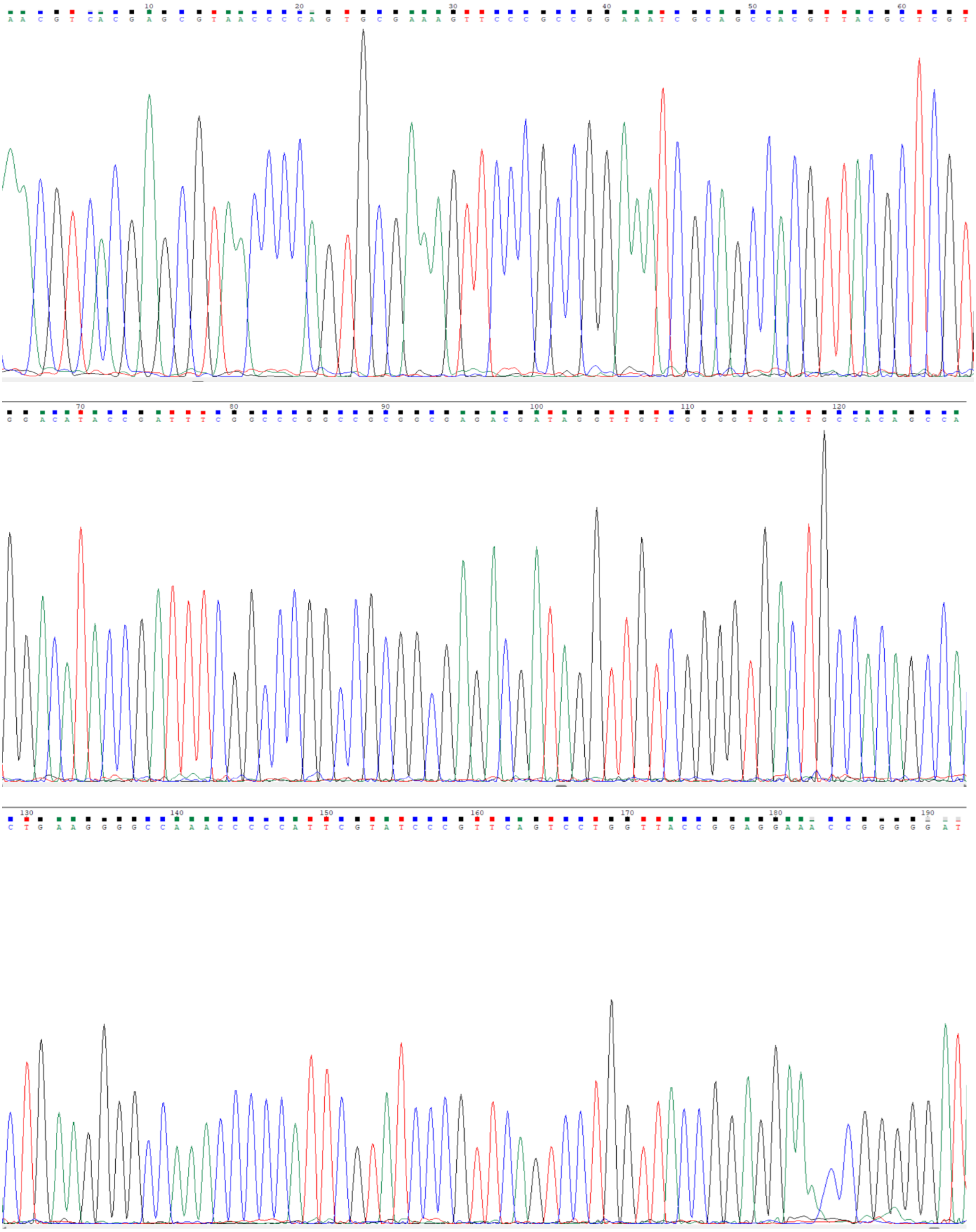

C17. Sequencing chromatograms of the *inhA* gene, covering nucleotide positions 10–190, show a region with no detected mutations.

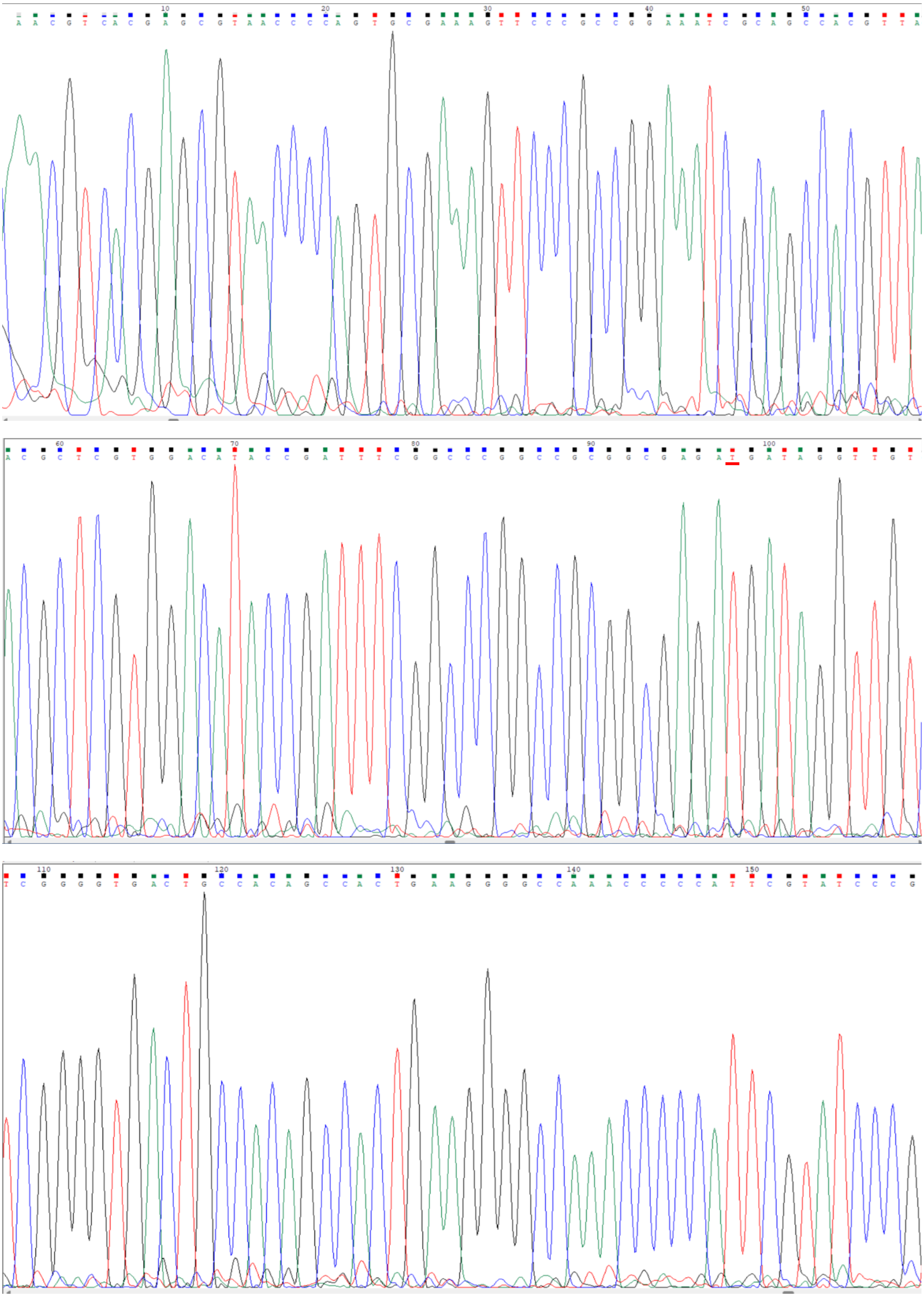

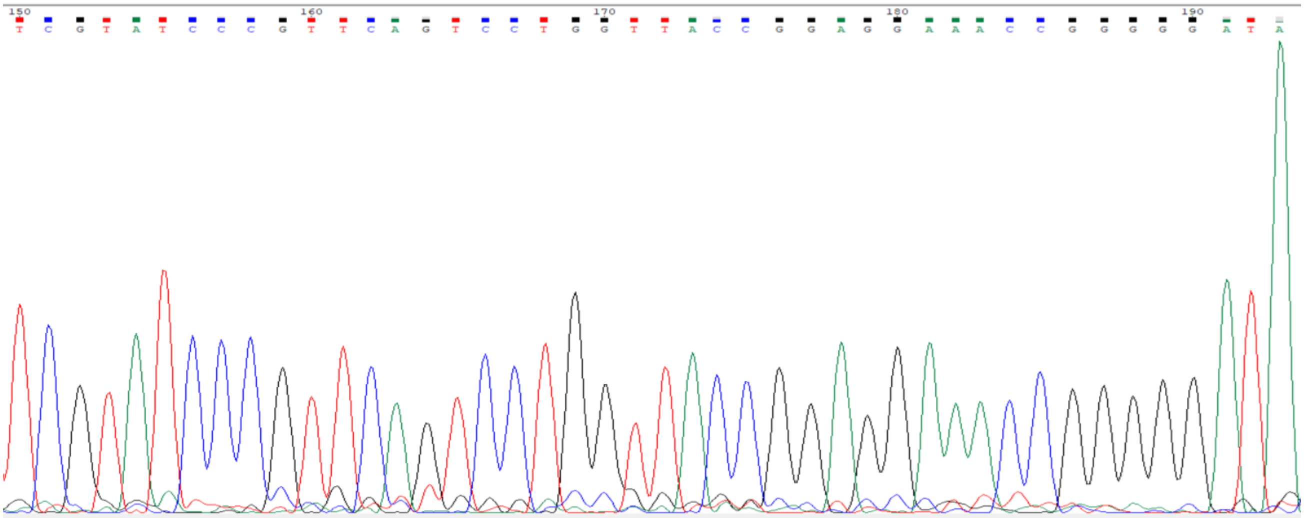

C18. Sequencing chromatograms of the *inhA* gene, covering nucleotide positions 10–190, show an **C→T** substitution between nucleotide positions 90–100.

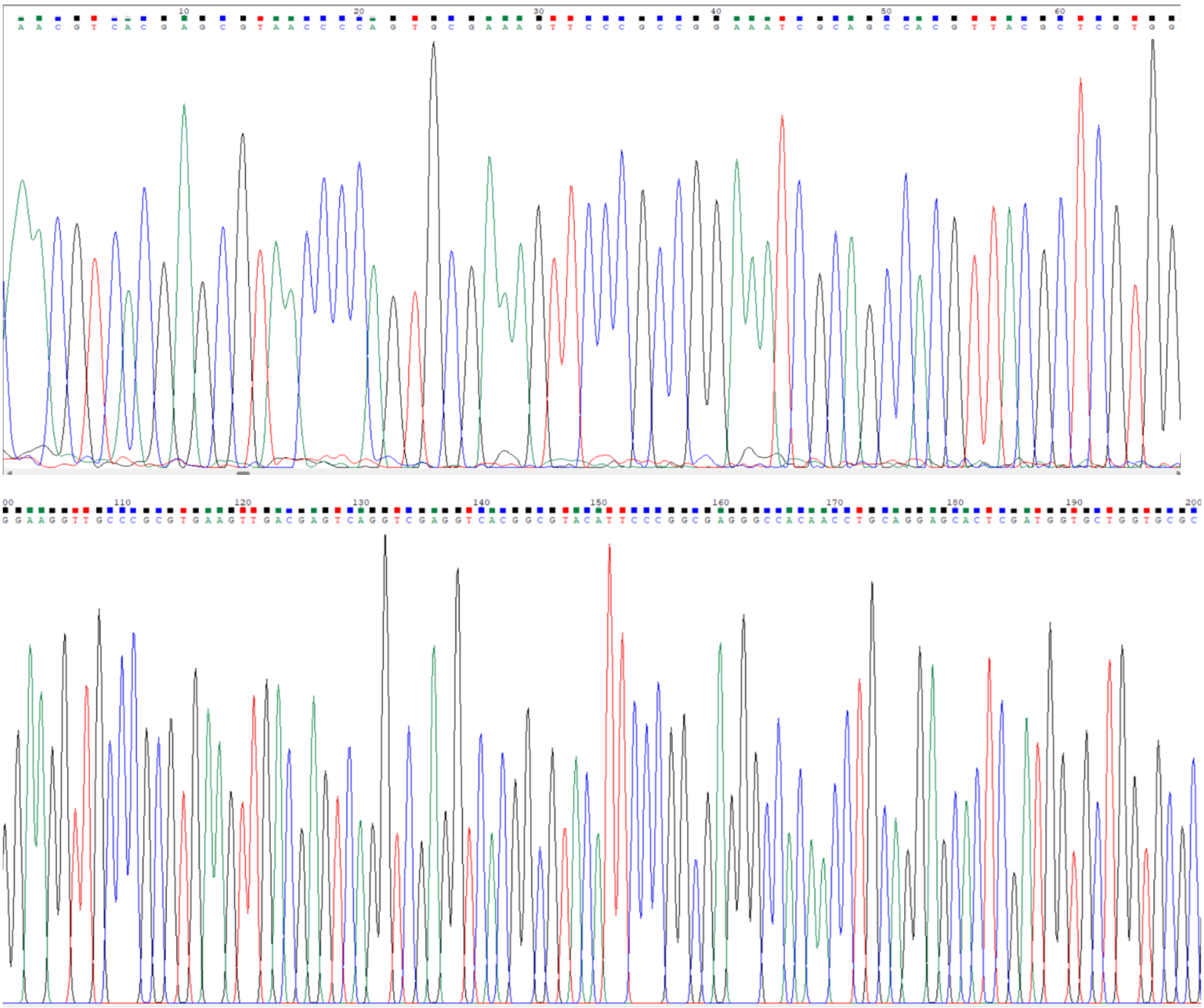

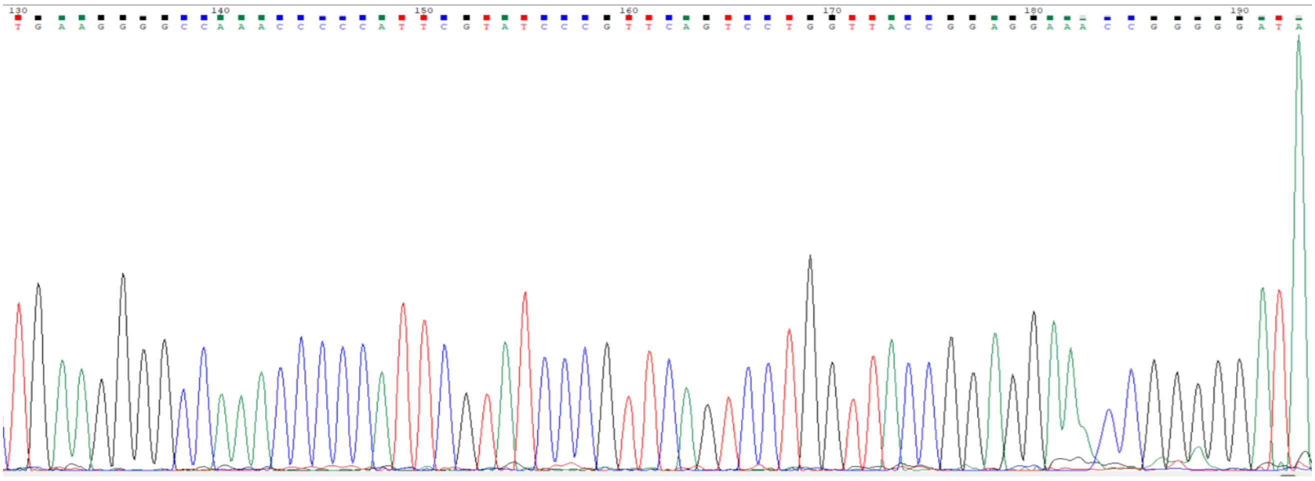

C19. Sequencing chromatograms of the *inhA* gene, covering nucleotide positions 10–190, show a region with no detected mutations.

Chromatogram images of the *rpoB* gene region from 6 isolates (D1-D6).

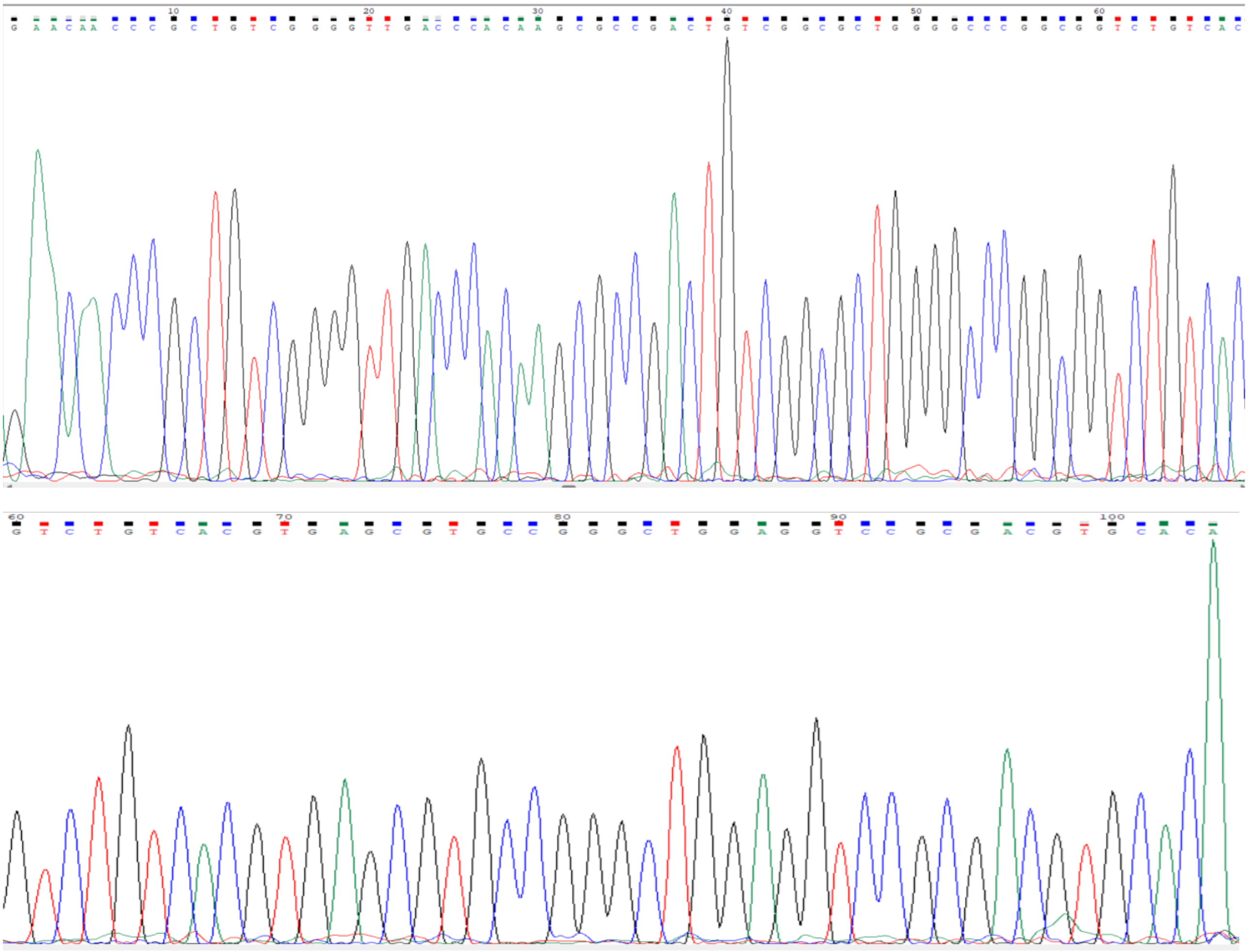

D1. Sequencing chromatograms of the *rpoB* gene, covering nucleotide positions 10–100, show a region with no detected mutations.

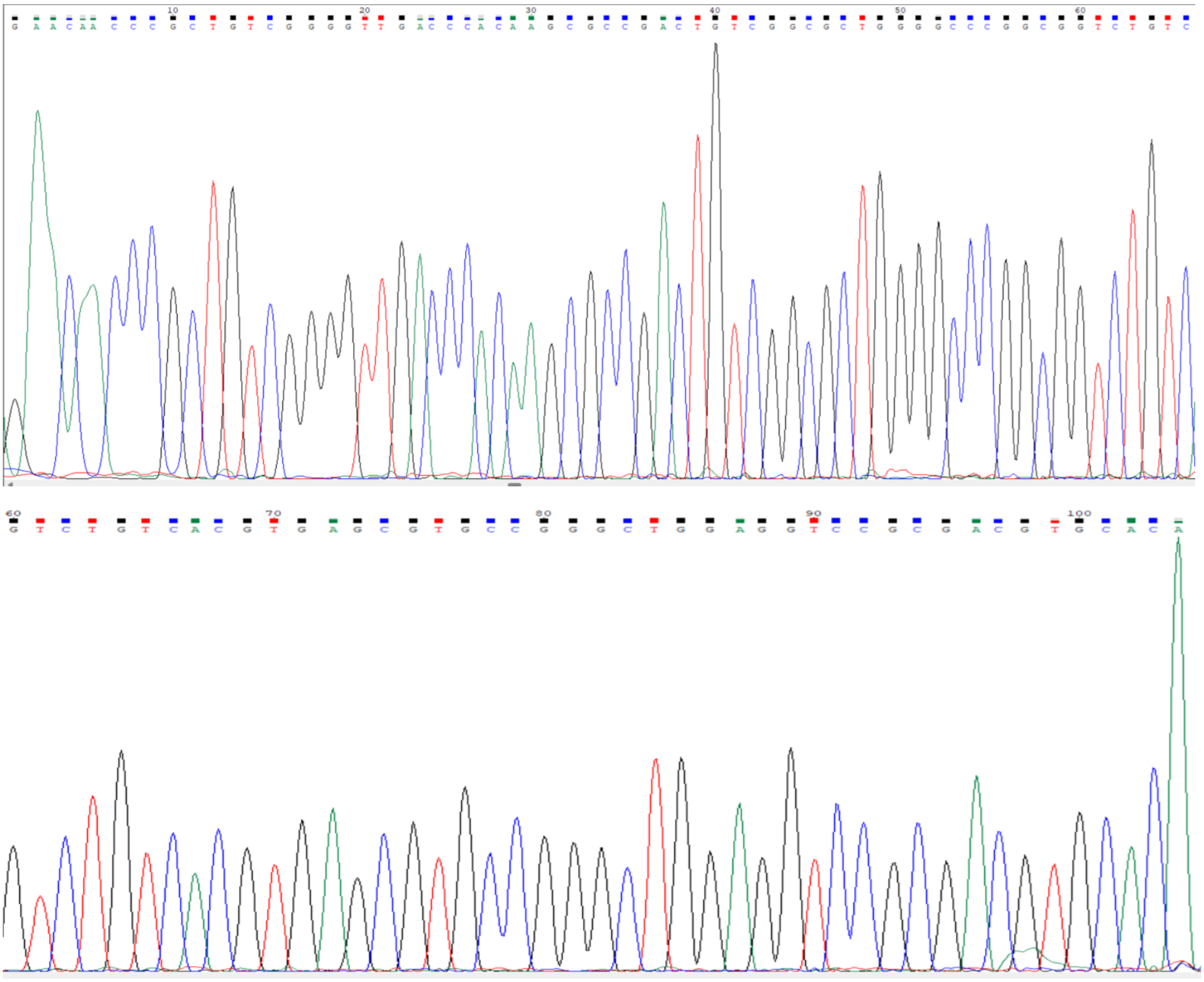

D2. Sequencing chromatograms of the *rpoB* gene, covering nucleotide positions 10–100, show a region with no detected mutations.

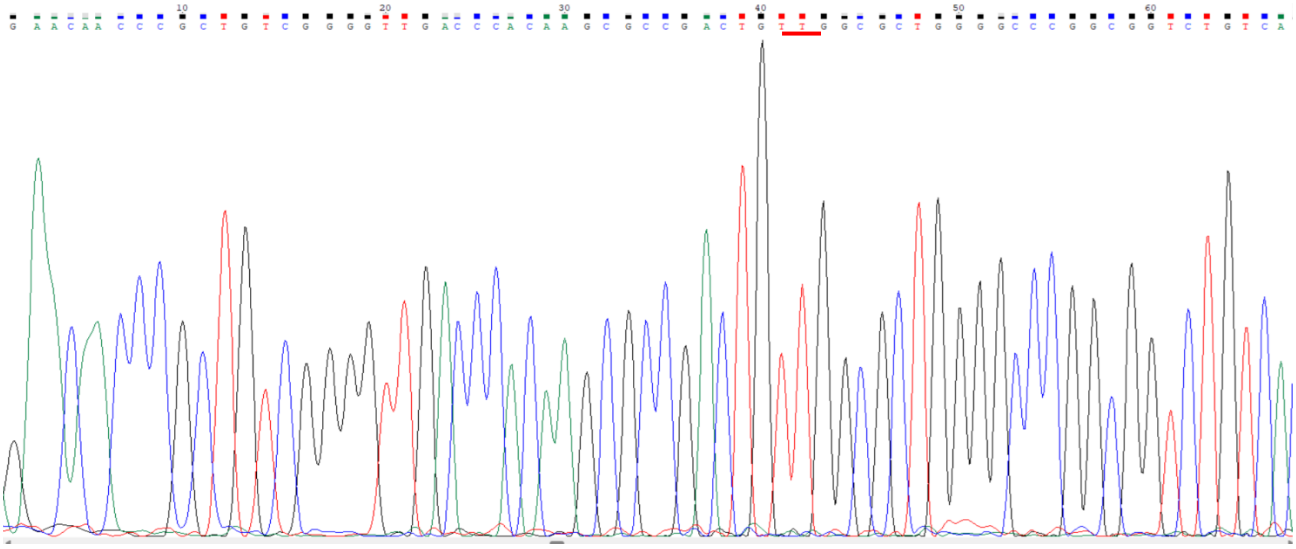

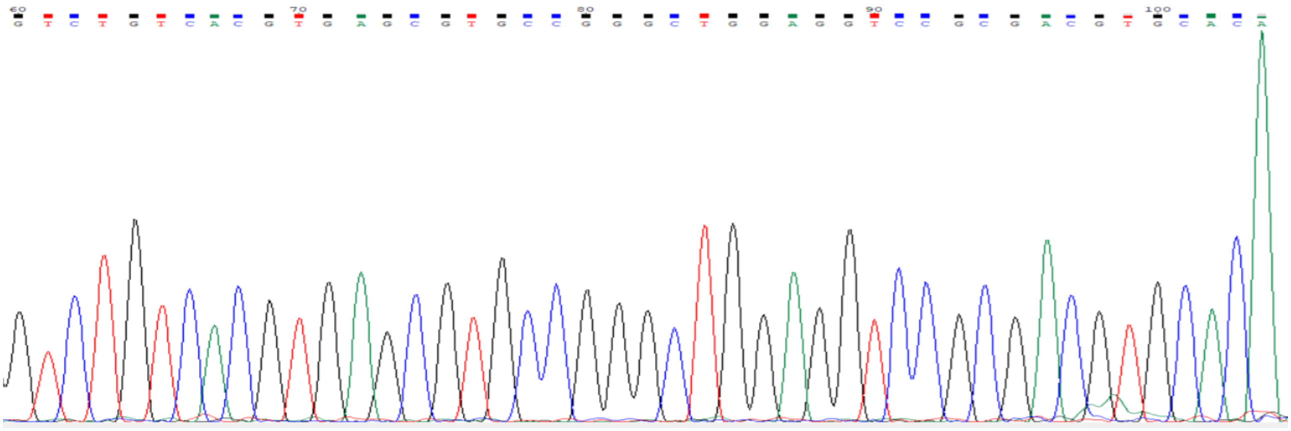

D3. Sequencing chromatograms of the *rpoB* gene, covering nucleotide positions 10 –100, show an **TCG→TTG** substitution between nucleotide positions 40–50.

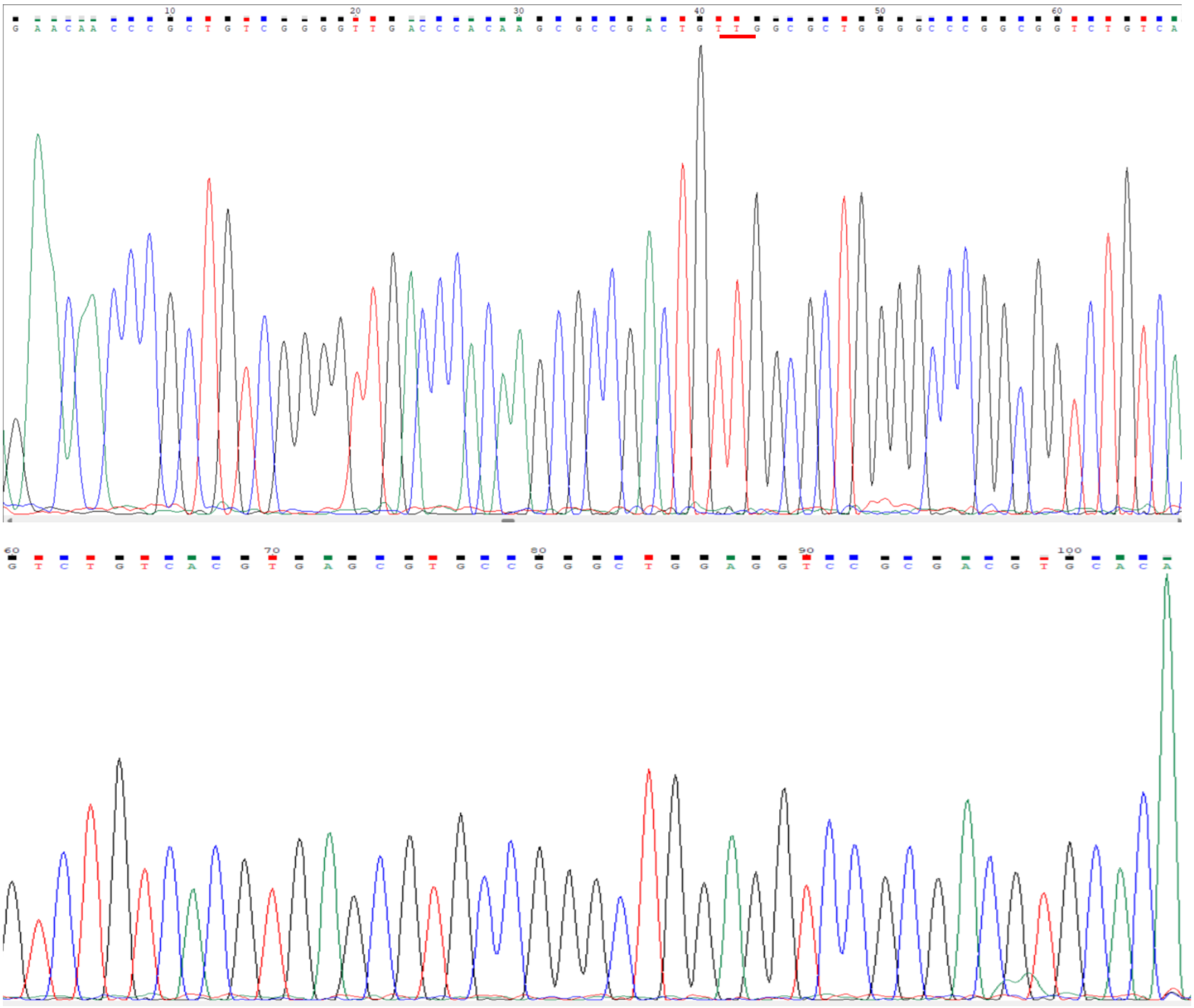

D4. Sequencing chromatograms of the *rpoB* gene, covering nucleotide positions 10 –100, show an **TCG→TTG** substitution between nucleotide positions 40–50.

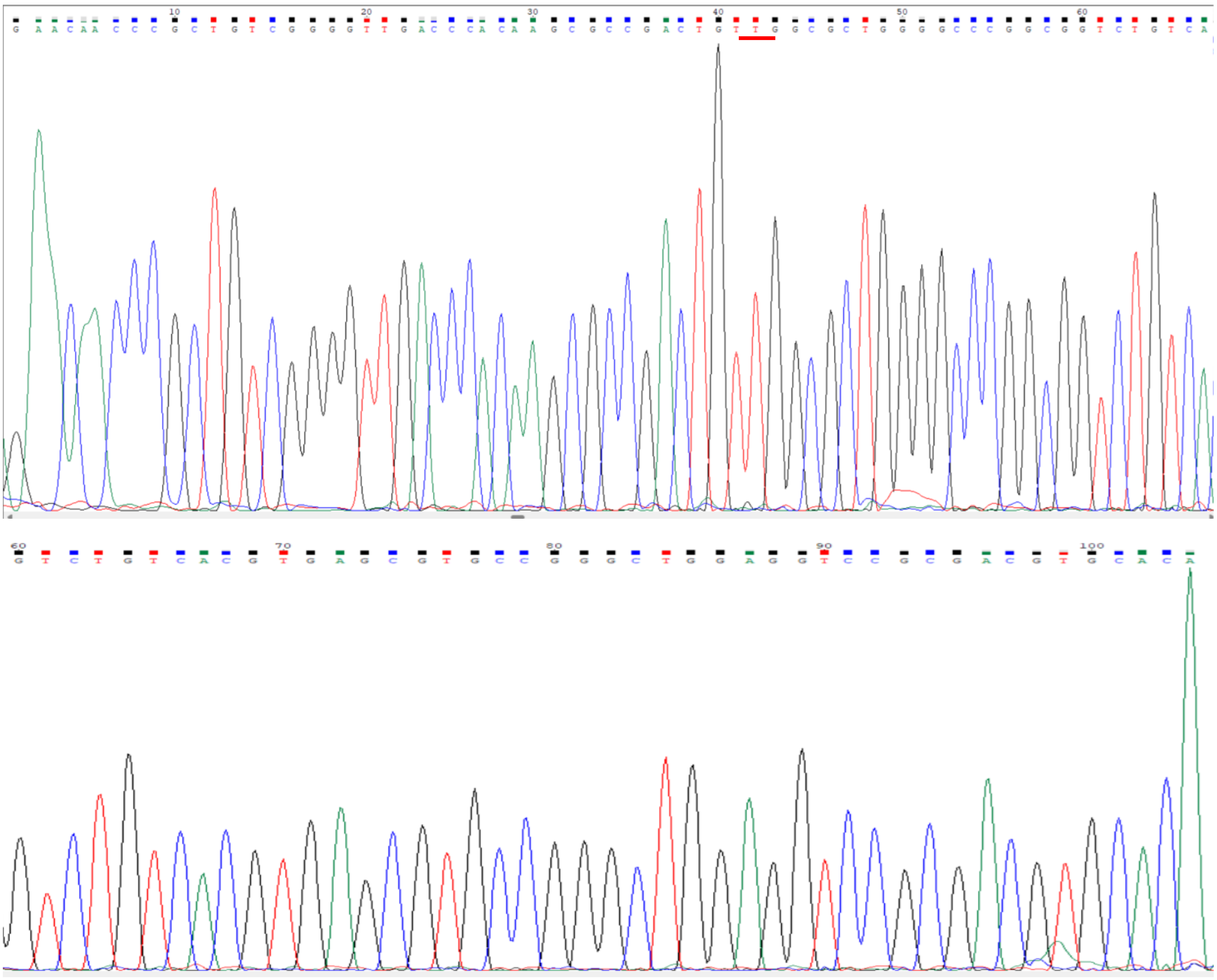

D5. Sequencing chromatograms of the *rpoB* gene, covering nucleotide positions 10 –100, show an **TCG→TTG** substitution between nucleotide positions 40–50.

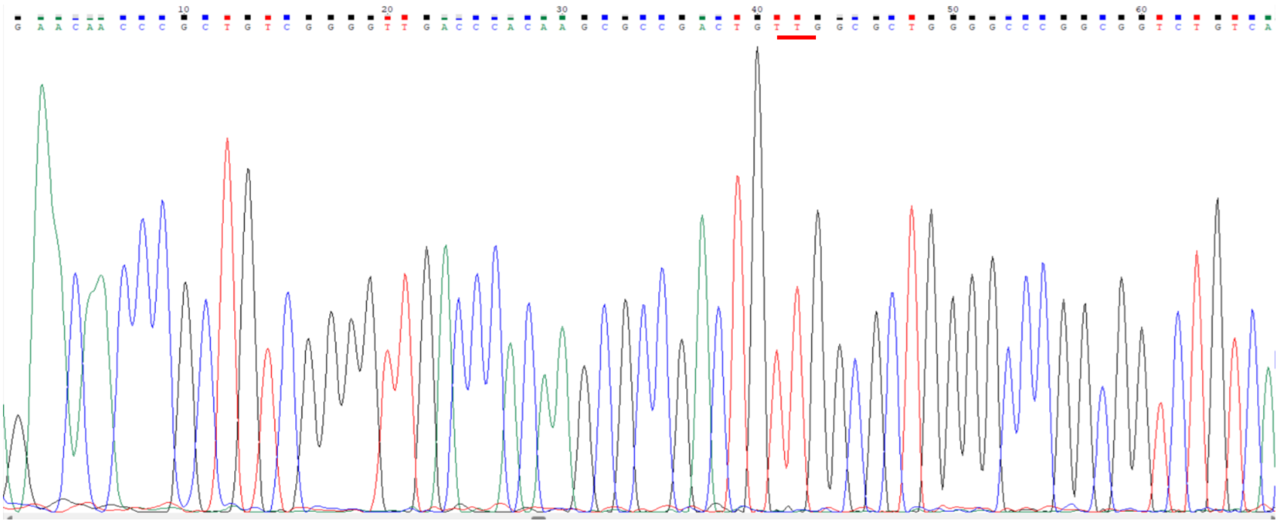

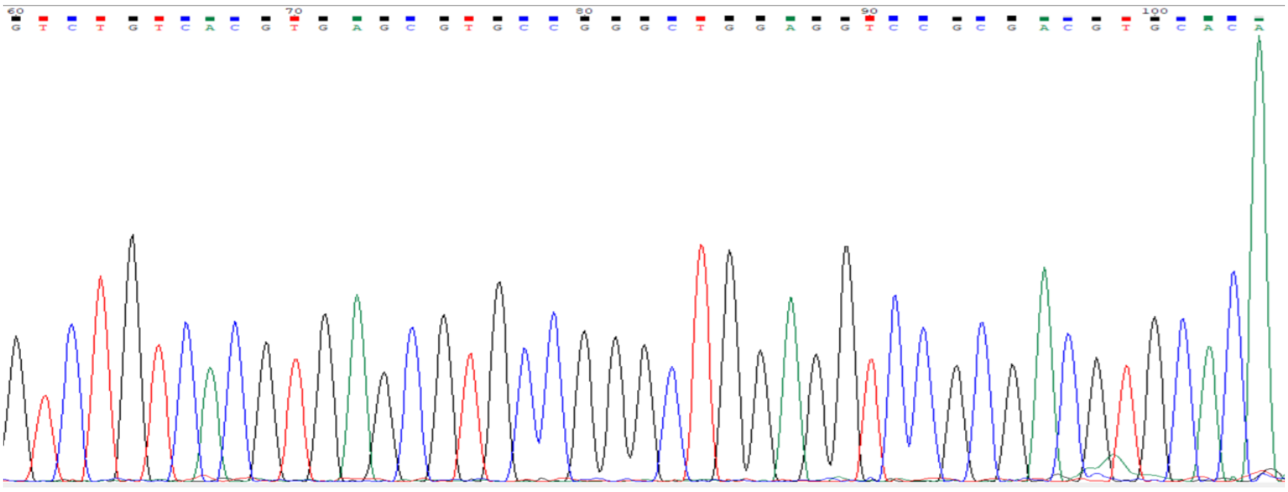

D6. Sequencing chromatograms of the *rpoB* gene, covering nucleotide positions 10 –100, show an **TCG→TTG** substitution between nucleotide positions 40–50.

Chromatogram images of the *embB* gene region from 4 isolates (E1-E4).

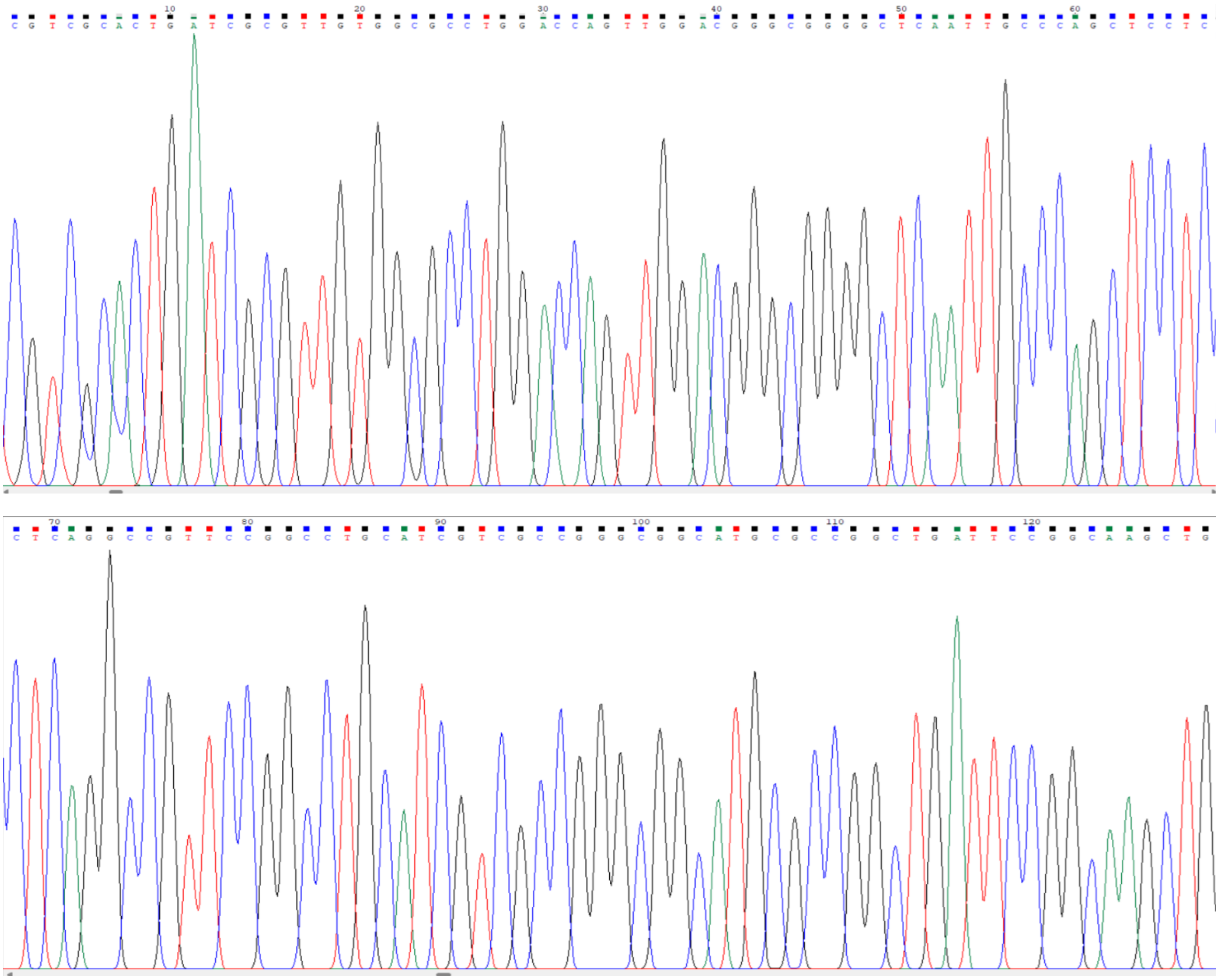

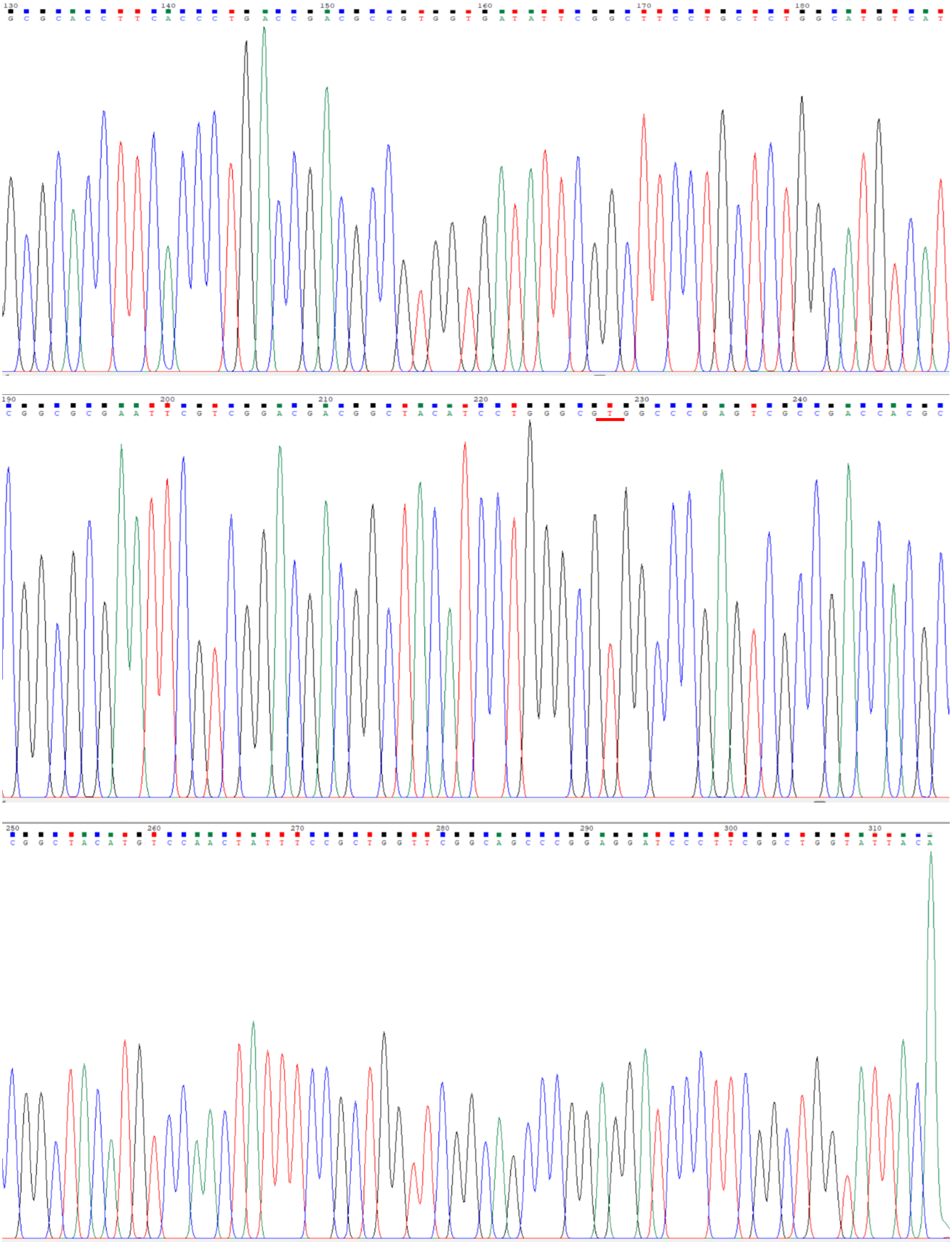

E1. Sequencing chromatograms of the *embB* gene, covering nucleotide positions 10 –310, show an ATG→GTG substitution between nucleotide positions 220–230.

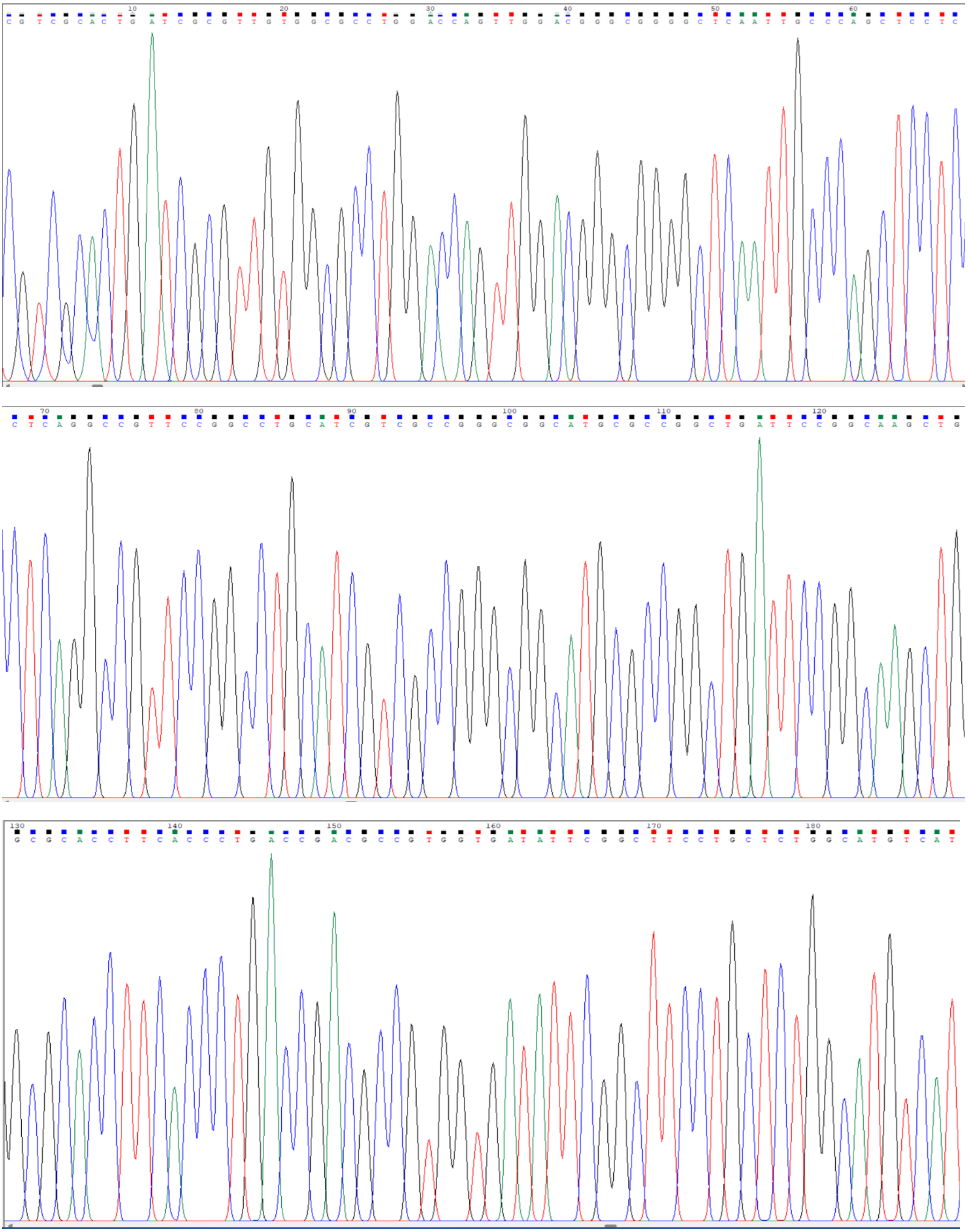

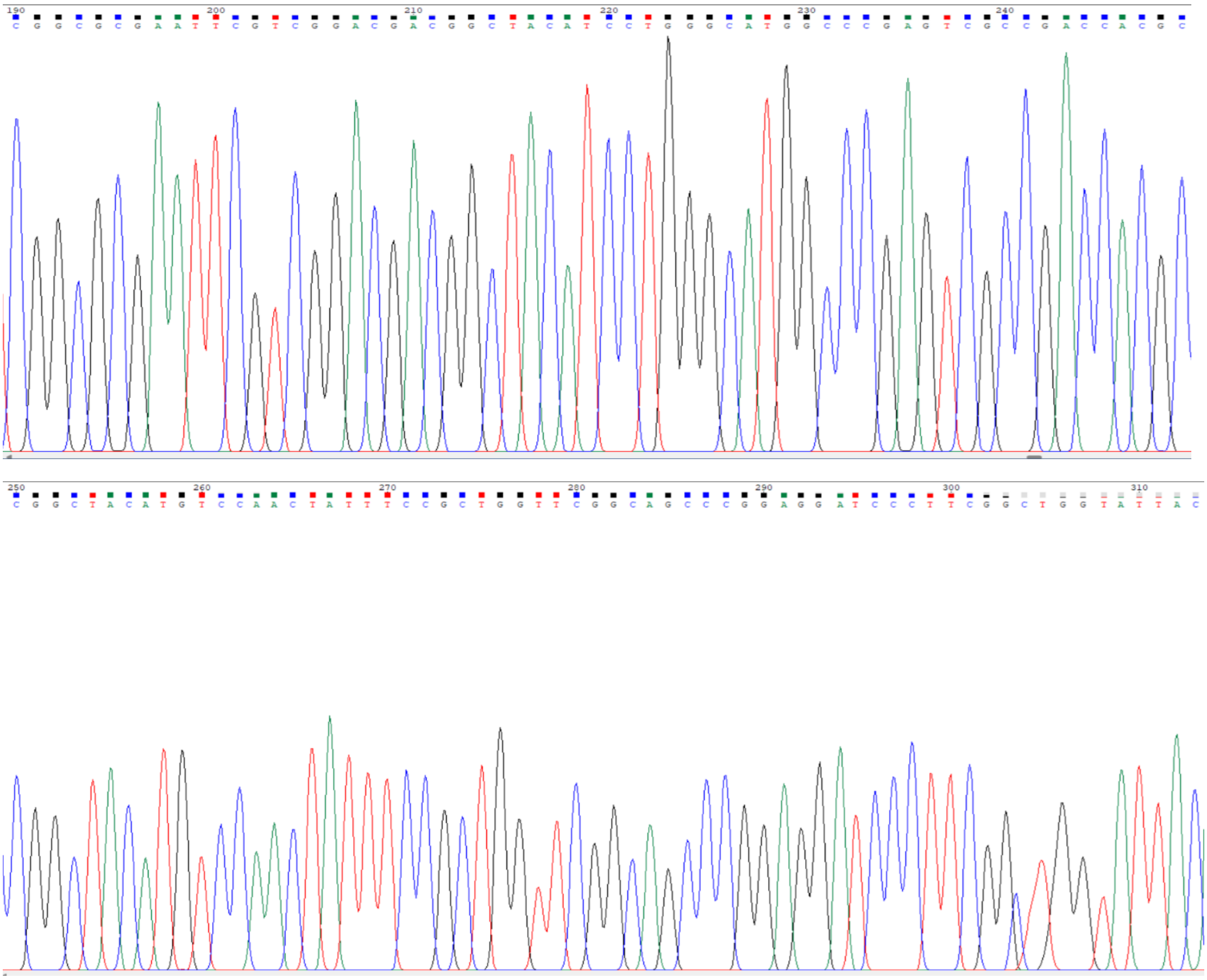

E2. Sequencing chromatograms of the *embB* gene, covering nucleotide positions 10–310, show a region with no detected mutations.

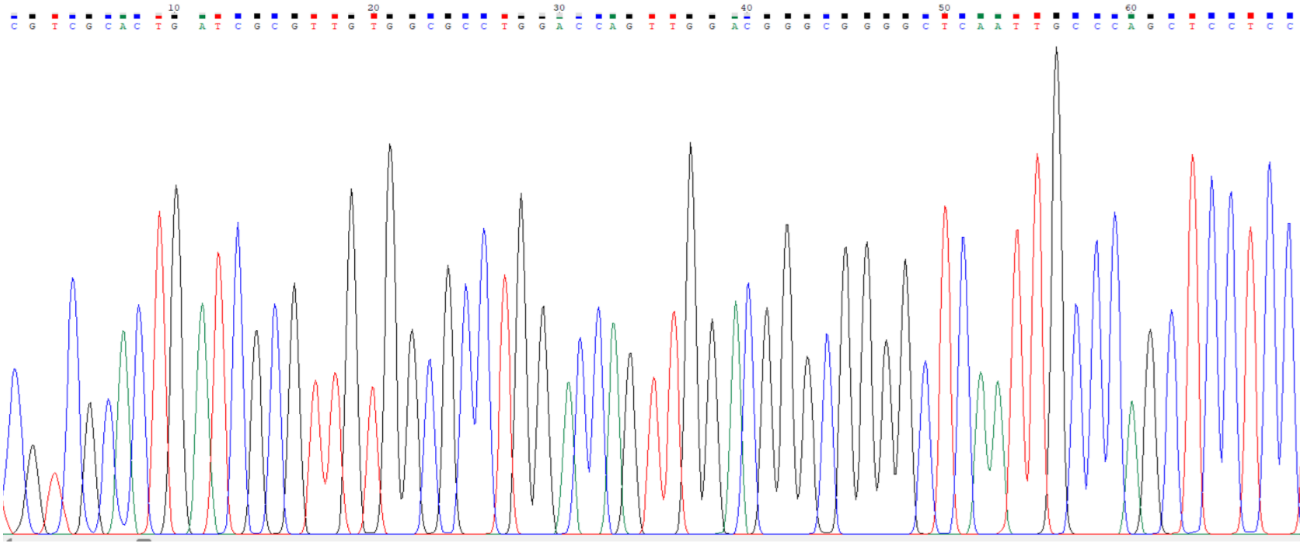

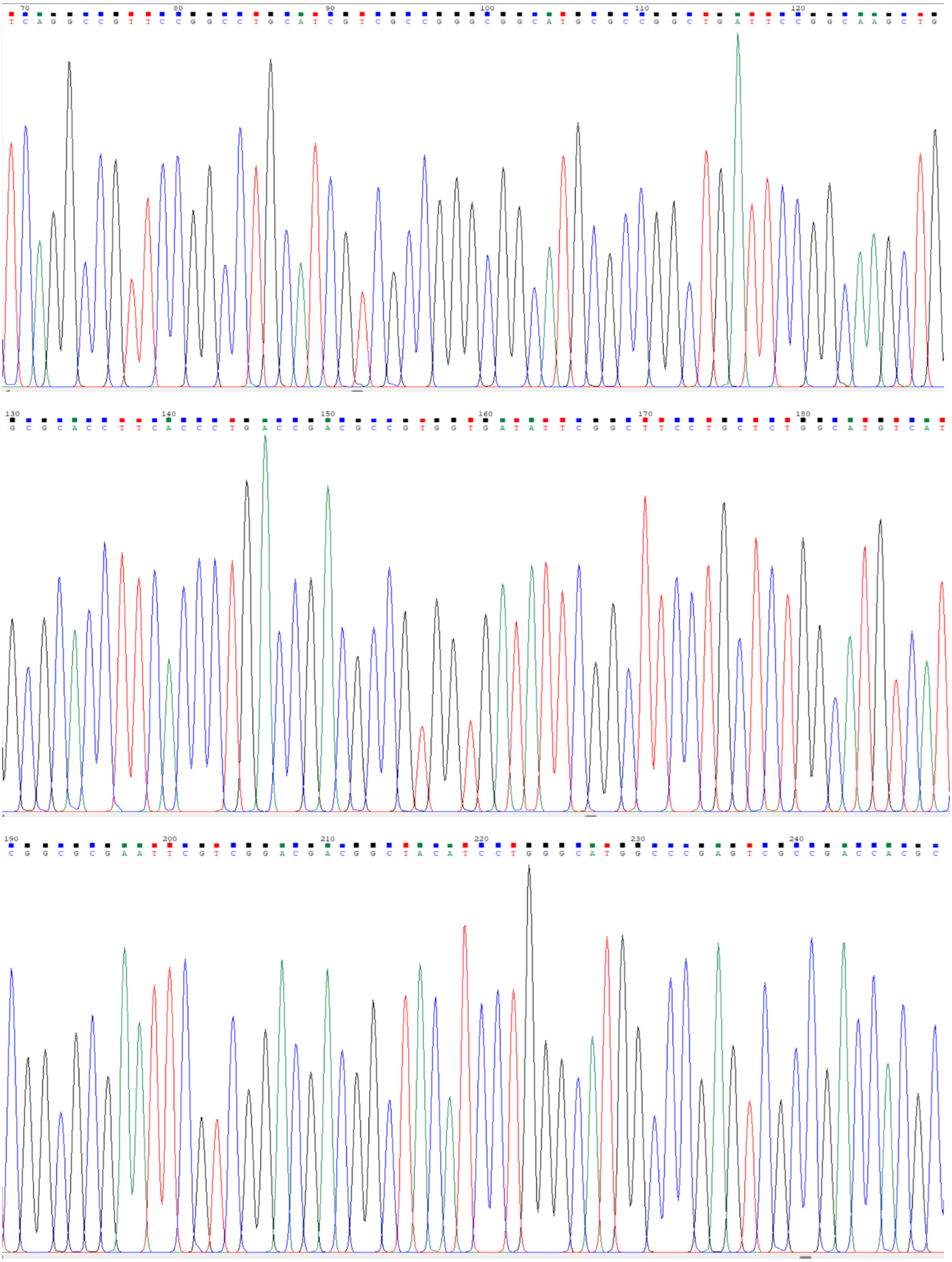

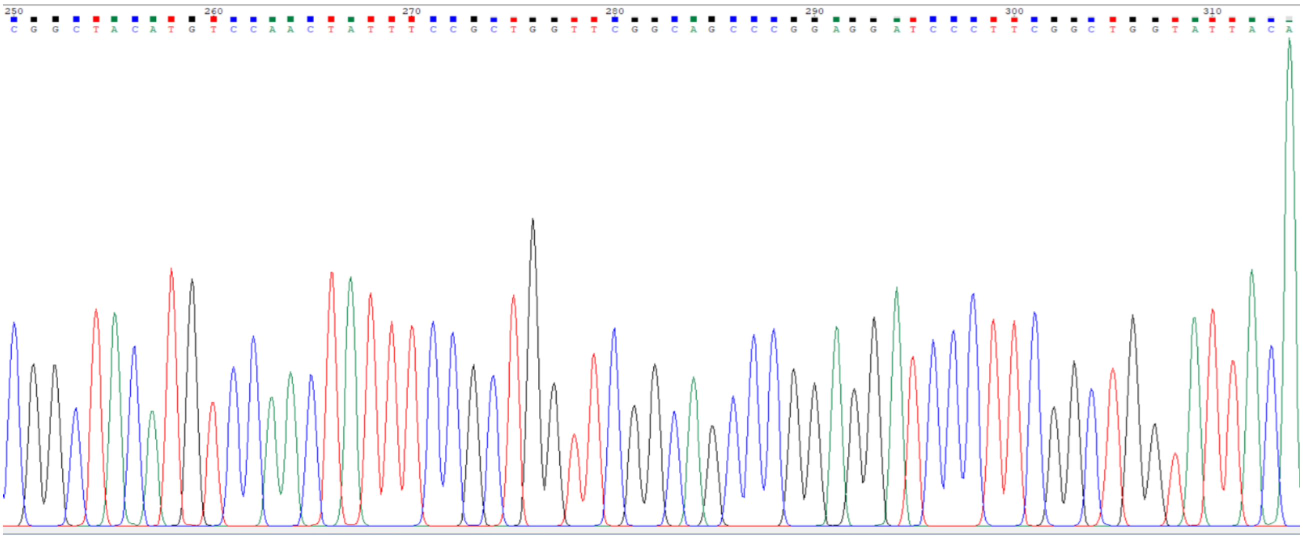

E3. Sequencing chromatograms of the *embB* gene, covering nucleotide positions 10–310, show a region with no detected mutations.

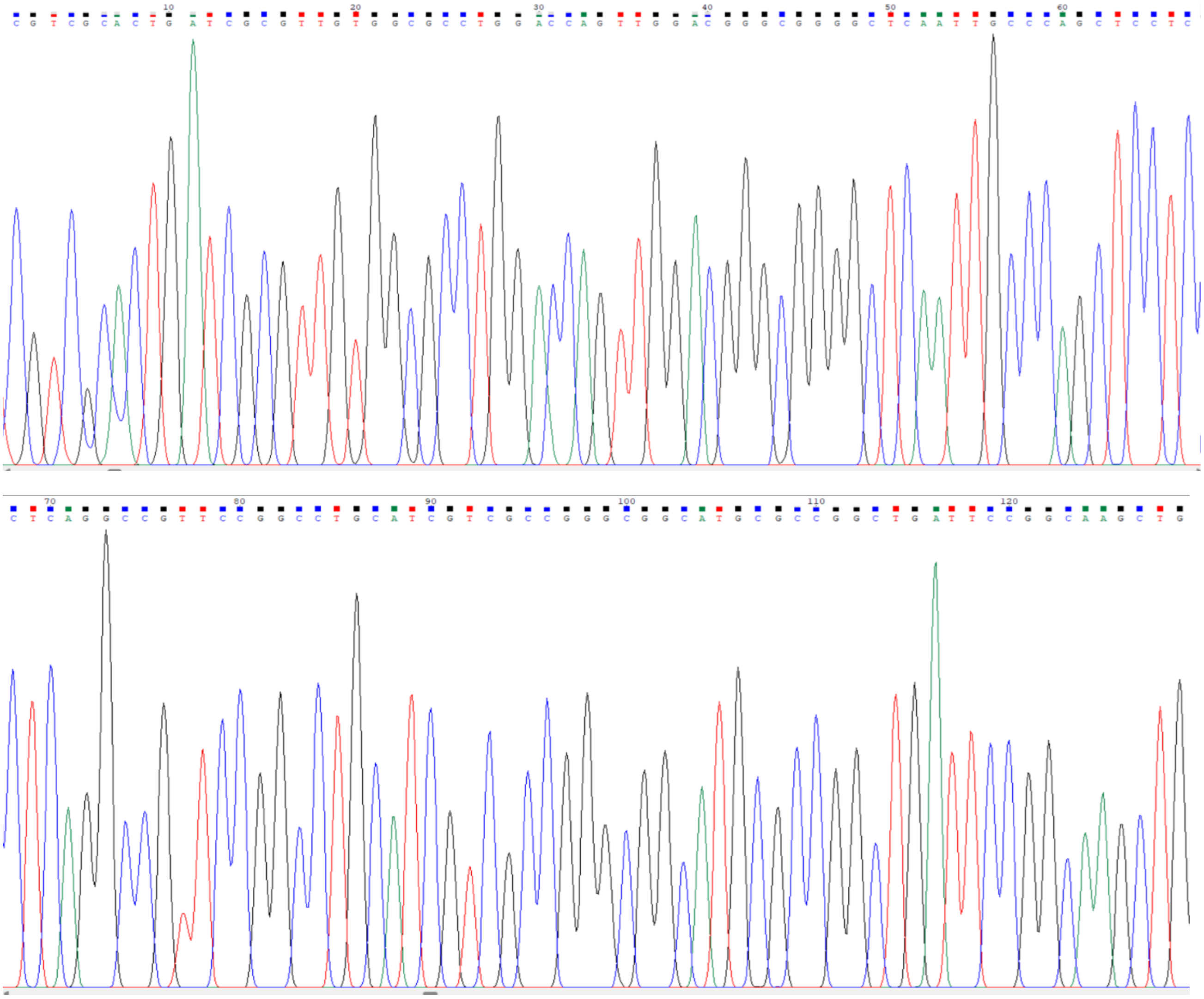

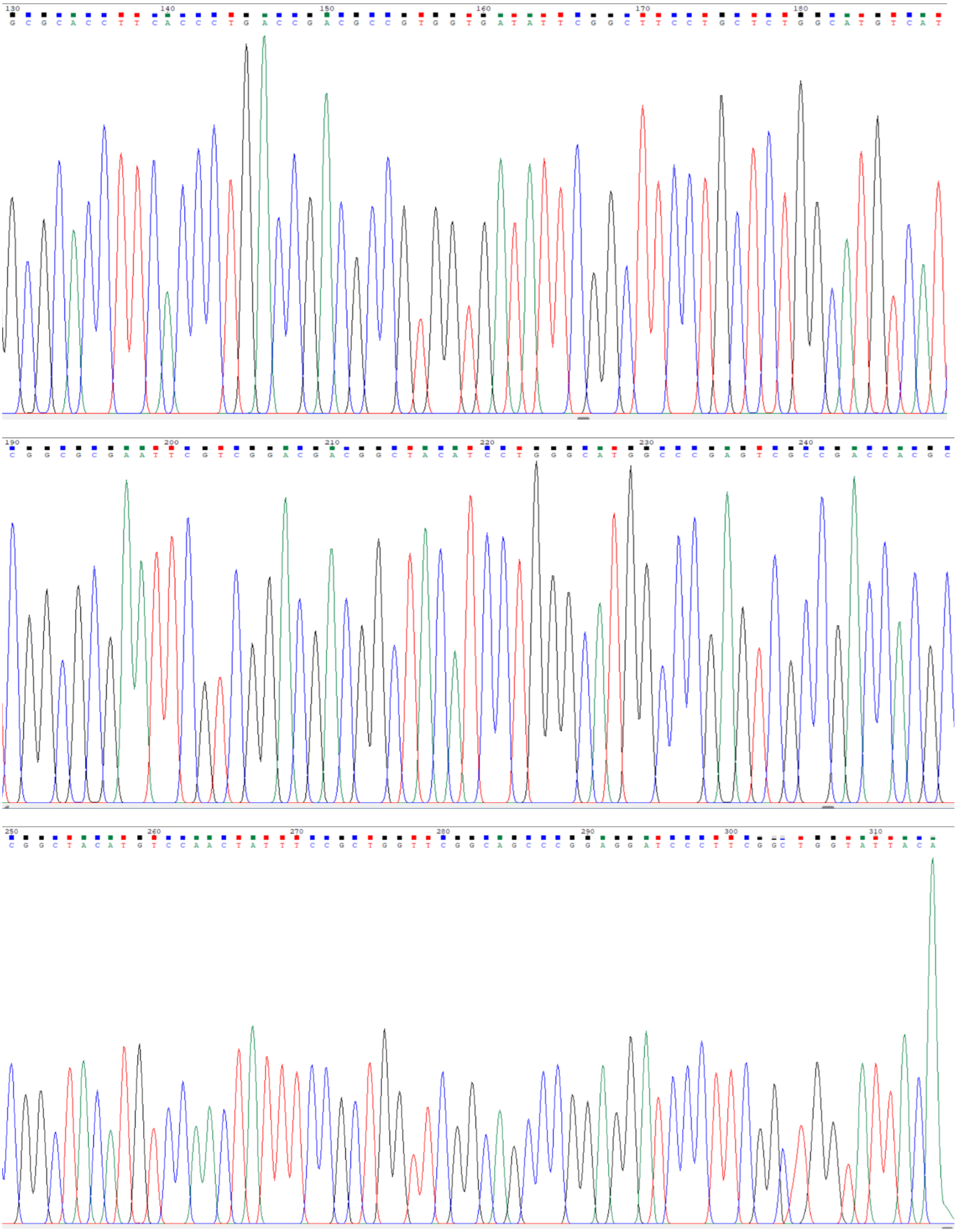

E4. Sequencing chromatograms of the *embB* gene, covering nucleotide positions 10–310, show a region with no detected mutations.
